# High-Throughput Antigen Microarray Identifies Longitudinal Prognostic Autoantibody for Chemoimmunotherapy in Advanced Non-Small Cell Lung Cancer

**DOI:** 10.1016/j.mcpro.2024.100749

**Published:** 2024-03-20

**Authors:** Liyuan Dai, Qiaoyun Tan, Lin Li, Ning Lou, Cuiling Zheng, Jianliang Yang, Liling Huang, Shasha Wang, Rongrong Luo, Guangyu Fan, Tongji Xie, Jiarui Yao, Zhishang Zhang, Le Tang, Yuankai Shi, Xiaohong Han

**Affiliations:** 1 Department of Clinical Laboratory, National Cancer Center/National Clinical Research Center for Cancer/Cancer Hospital, Chinese Academy of Medical Sciences & Peking Union Medical College, Beijing Key Laboratory of Clinical Study on Anticancer Molecular Targeted Drugs, Beijing, China; 2 Department of Medical Oncology, National Cancer Center/National Clinical Research Center for Cancer/Cancer Hospital, Chinese Academy of Medical Sciences & Peking Union Medical College, Beijing Key Laboratory of Clinical Study on Anticancer Molecular Targeted Drugs, Beijing, China; 3 Department of Pathology, National Cancer Center/National Clinical Research Center for Cancer/Cancer Hospital, Chinese Academy of Medical Sciences & Peking Union Medical College, Beijing, China; 4 Clinical Pharmacology Research Center, Peking Union Medical College Hospital, State Key Laboratory of Complex Severe and Rare Diseases, NMPA Key Laboratory for Clinical Research and Evaluation of Drug, Beijing Key Laboratory of Clinical PK & PD Investigation for Innovative Drugs, Chinese Academy of Medical Sciences & Peking Union Medical College, Beijing, China

**Keywords:** autoantibody, chemoimmunotherapy, prognostic biomarker, advanced non-small cell lung cancer, therapeutic monitoring

## Abstract

Chemoimmunotherapy has evolved as a standard treatment for advanced non-small cell lung cancer (aNSCLC). However, inevitable drug resistance has limited its efficacy, highlighting the urgent need for biomarkers of chemoimmunotherapy. A three-phase strategy to discover, verify, and validate longitudinal predictive autoantibodies (AAbs) for aNSCLC before and after chemoimmunotherapy was employed. A total of 528 plasma samples from 267 aNSCLC patients before and after anti-PD1 immunotherapy were collected, plus 30 independent formalin-fixed paraffin-embedded samples. Candidate AAbs were firstly selected using a HuProt high-density microarray containing 21,000 proteins in the discovery phase, followed by validation using an aNSCLC-focused microarray. Longitudinal predictive AAbs were chosen for ELISA based on responders *versus* non-responders comparison and progression-free survival (PFS) survival analysis. Prognostic markers were also validated using immunohistochemistry and publicly available immunotherapy datasets. We identified and validated a panel of two AAbs (MAX and DHX29) as pre-treatment biomarkers and another panel of two AAbs (MAX and TAPBP) as on-treatment predictive markers in aNSCLC patients undergoing chemoimmunotherapy. All three AAbs exhibited a positive correlation with early responses and PFS (*p* < 0.05). The kinetics of MAX AAb showed an increasing trend in responders (*p* < 0.05) and a tendency to initially increase and then decrease in non-responders (*p* < 0.05). Importantly, MAX protein and mRNA levels effectively discriminated PFS (*p* < 0.05) in aNSCLC patients treated with immunotherapy. Our results present a longitudinal analysis of changes in prognostic AAbs in aNSCLC patients undergoing chemoimmunotherapy.

## Highlights

• Autoantibodies can monitor responses in aNSCLC undergoing chemoimmunotherapy
• MAX AAb showed longitudinal changes discrepancies in responders and non-responders.
• MAX was associated with superior outcomes in aNSCLC receiving immunotherapy.

## Graphical Abstract

**Figure:**
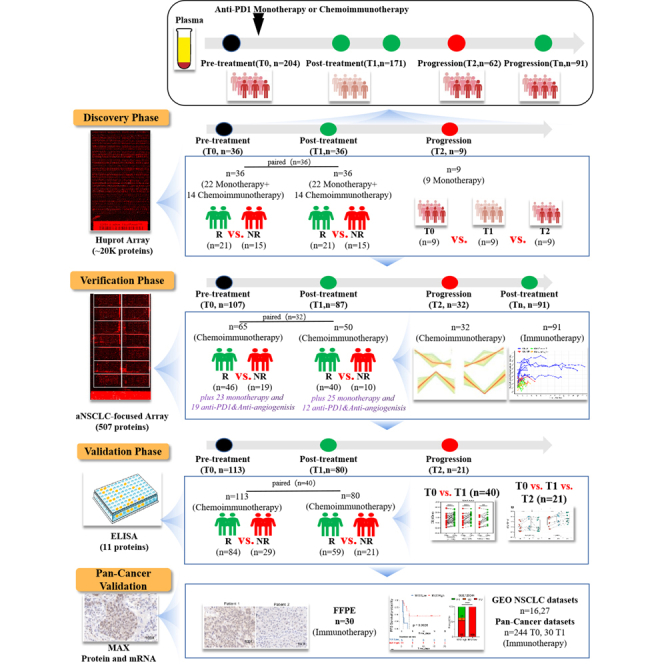

## In Brief

This study screens 21,000 autoantibodies in aNSCLC patients undergoing ICIs monotherapy and chemoimmunotherapy, identifying prognostic markers—MAX, DHX29, and TAPBP AAbs—for early progression and progression-free survival. This systematic approach involves *de novo* screening, identification, and validation, providing clinical potential to monitor chemoimmunotherapy efficacy and detect disease progression.

According to the 2020 GLOBOCAN report, lung cancer ranks the second in global incidence (11.4%) and has the highest mortality rate (18%) among all cancers (1). Non-small cell lung cancer (NSCLC) accounts for the majority (85%) of these cases. Due to the absence of effective screening methods and the presence of atypical early clinical symptoms, most NSCLC patients are diagnosed at advanced stages, leading to poor prognosis (2). The treatment options for advanced non-small cell lung cancer (aNSCLC) have shifted from surgery, radiotherapy, and chemotherapy to targeted therapy and immunotherapy. Immune checkpoint inhibitors (ICIs) targeting programmed death-1/ligand-1 (PD-1/L1) have demonstrated remarkable success in activating patients' immune systems and significantly improving survival rates in aNSCLC. However, with non-responders constituting three-quarters of NSCLC patients under immunotherapy (3, 4), there is an urgent need for more effective drugs or combination therapies (5, 6). Combining ICIs with chemotherapy has demonstrated synergistic effects, resulting in substantial improvements in overall response rates (ranging from 28.4% to 63.5%) and survival (7). However, currently available prognostic biomarkers such as PD-L1 expression, tumor mutational burden (TMB), and microsatellite instability-high/deficient mismatch repair (MSI-H/dMMR) show modest predictive power (8, 9). Moreover, variations in PD-L1 expression thresholds, the incidence range of MSI-H/dMMR in cancers (1% to 25%) (10), the dependence of TMB tests reliability on tissue specimens quality, and the limited accessibility of biopsy specimens for aNSCLC patients underscore the urgency in identifying novel, tailored prognostic markers to predict the efficacy of ICIs therapy.

Dynamic monitoring of biomarker changes during tumor-immune system interactions using liquid biopsies provides valuable insights into tumor heterogeneity and helps mitigate inter-individual variability. Previous studies have identified various biomarkers for dynamic monitoring, including peripheral blood circulating tumor DNA (11, 12), neutrophil-to-lymphocyte ratio (13), C-reactive protein (14), interleukin-6 (15), interleukin-8 (16, 17), CXCL8, and CXCL10 (18). In recent years, the advantages of utilizing peripheral blood autoantibodies (AAbs) have gained recognition due to their accessibility, high specificity, and non-invasiveness. AAbs have demonstrated substantial potential in the early diagnosis of lung cancer, exemplified by the EarlyCDT Lung test (19), as well as their predictive value in assessing the efficacy of ICIs therapy, prognosis, and prediction of immune-related adverse events, thereby generating enormous clinical interest. In NSCLC, AAbs panels associated with autoimmune diseases (antinuclear, thyroglobulin, thyroid peroxidase AAbs) (20) and solely AAb (such as lgM-RF (21), NY-ESO-1, XAGE1 (22), p53, BRCA2, HUD, and tripartite motif-containing-21 (TRIM21) AAbs (23)) have been reported to be associated with ICIs therapy efficacy. However, existing studies have predominantly focused on baseline plasma AAb levels, lacking high-throughput screening, and comprehensive pre-treatment combined with post-treatment dynamic monitoring of AAbs in multistage cohorts, particularly in chemoimmunotherapy. Addressing this gap through extensive pre- and post-treatment dynamic monitoring of AAbs *via* high-throughput screening in aNSCLC patients receiving ICIs therapy is essential, as it promises a deeper comprehension of ICIs therapy response, particularly in conjunction with chemotherapy, and aids in the formulation of personalized treatment strategies for NSCLC patients.

Our previous studies demonstrated the predictive potential of pre-treatment plasma levels of homeobox protein SIX2 (SIX2) AAb (24) and lgG4 AAb targeting programmed cell death protein 1 (PDCD1) (25) for ICIs therapy efficacy in NSCLC. However, longitudinal monitoring of these markers is lacking. The present study aimed to investigate the spectrum characteristics of AAbs in pre-treatment and on-treatment plasma samples from aNSCLC patients undergoing chemoimmunotherapy. We identified predictive and dynamic monitoring signatures for ICIs therapy response in three phases (discovery, verification, and validation). Furthermore, the prognostic value of AAbs’ targeted proteins and mRNA were validated using immunohistochemistry (IHC) and external public databases. In summary, our comprehensive findings contribute to the discovery of predictive AAb biomarkers and provide valuable insights into underlying mechanisms of resistance to chemoimmunotherapy. These findings have the potential to improve patient stratification and guide personalized treatment decisions, ultimately enhancing the outcomes of aNSCLC patients.

## Experimental Procedures

### Reagents and Tools Table

**Table:** 

Reagent/Resource	Reference or source	Catalog number
Samples		
Blood plasma samples	This study	N/A
Formalin-fixed paraffin-embedded samples	This study	N/A
RNA-seq samples	GSE135222	Ref. (26)
RNA-seq samples	GSE126044	Ref. (27)
Pan-cancer RNA-seq samples	https://kmplot.com/analysis/	N/A
Reagents		
Bovine serum albumin (BSA)	9048-46-8	Sigma
Alexa fluor 647 goat anti-human IgG	109-605-003	Jackson
TAPBP, PPP6R2, MED22, MAD2L1, SCYL1, LATS1, NDRG1, BCL10, TP53, and DHX29 proteins with glutathione-S-transferase (GST) tag	CDI laboratory (HuProt ID: JHU15270P160B02, JHU11491P120H11, JHU11024P115H09, JHU01303P014D08, JHU16467P173F05, JHU09360P098B08, JHU07452P078G08, JHU06828P072F03, JHU04788P050D05, JHU14294P150C09)	N/A
MAX protein with histidine 6 (His-6) tag	#AP89669	SAB
Anti-GST-tag rabbit antibody	ab184804	Abcam
HRP-labeled goat anti-human lgG antibody	109-035-088	Jackson
Tetramethyl benzidine	abs9178	Absin
Sulfuric acid	abs9191	Absin
Rabbit anti-human MAX IgG antibody	ab101271	Abcam
HRP-labeled goat anti-rabbit IgG antibody	GB23303	Servicebio
Diaminobenzidine (DAB)	G1212	Servicebio
Hematoxylin	G1004	Servicebio
Software		
GenePix Pro v.6.0	Molecular Devices	N/A
SkanIt Software for Microplate Readers DDE, v.5.0.0.42	Thermo Fisher Scientific	N/A
CaseViewer 2.4	3DHISTECH, Hungary	N/A
Gene Expression Omnibus (GEO)	http://www.ncbi.nlm.nih.gov/geo	N/A
Kaplan-Meier Plotter	https://kmplot.com/analysis/	N/A
IBM SPSS Statistics 24	https://www.ibm.com/support/pages/	N/A
GraphPad Prism 9	https://www.graphpad-prism.cn/	N/A
R(4.2.1)	https://www.R-project.org/	N/A
Metascape	https://metascape.org/gp/index.html	N/A
Sanger plot website	http://www.sangerbox.com	N/A
CAMOIP database	http://www.camoip.net/	N/A
GEPIA	http://gepia.cancer-pku.cn/	N/A
Instrument		
Genepix 4300A microarray scanner	Molecular Devices	141095
Multiskan Go microplate reader	Thermo Fisher Scientific	51119200
Microscope	Nikon	E100

### Methods and Protocols

#### Study Populations and Samples Collection

Between 2016 and 2022, a total of 528 plasma samples were obtained, including 204 pre-treatment (T0), 171 post-treatment (T1) within 3 months of ICIs therapy, 62 post-treatment (T2) at progression evaluation, and 91 additional post-treatment (Tn) plasma samples, and 30 formalin-fixed paraffin-embedded (FFPE) samples from 267 aNSCLC patients who received ICIs monotherapy (nivolumab, pembrolizumab, sintilimab, triplimab, camrelizumab, or tirellizumab), ICIs combined with chemotherapy (*cis*-platinum, carboplatin, pemetrexed, or docetaxel), or ICIs combined with an angiogenesis inhibitor (anlotinib or bevacizumab) at the Cancer Hospital, Chinese Academy of Medical Sciences. All plasma samples were collected using ethylenediaminetetraacetic acid, centrifuged at 3000 rpm and 4 °C for 10 min, and stored in 2 ml conical tubes at −80 °C until the microarray assay. FFPE samples were stored at room temperature.

The inclusion criteria for patients were as follows: (1) biopsy-confirmed aNSCLC with stage III or IV disease and complete clinical follow-up data; (2) aNSCLC patients receiving ICIs monotherapy, ICIs combined with chemotherapy (platinum, taxane, or pemetrexed), or angiogenesis inhibitors (anlotinib or bevacizumab); and (3) use of ICIs as first-line or later-line treatment. Patients meeting any of these conditions were excluded: (1) aNSCLC patients with other concurrent cancers; (2) aNSCLC patients with non-primary lung tumors; (3) aNSCLC patients having concomitant autoimmune diseases; and (4) aNSCLC patients using other immunosuppressive agents (*e.g.*, steroid medication). The efficacy of immunotherapy was evaluated using Response Evaluation Criteria in Solid Tumours (RECIST) version 1.1. In this study, based on the 3-month efficacy of ICIs treatment, patients were categorized as “responders” if they achieved complete remission, partial remission, or stable disease, and “non-responders” if they experienced disease progression. This study has been approved by the Ethics Committee of the National Cancer Center/National Clinical Research Center for Cancer/Cancer Hospital, Chinese Academy of Medical Sciences & Peking Union Medical College (No. 19-019/1804). All experiments were executed according to the Declaration of Helsinki principles.

#### HuProt Microarray and aNSCLC-Focused Microarray

Standard experimental procedures for high-density microarrays have been previously described (28). In summary, the HuProt microarray from CDI Labs consisted of approximately 21,000 proteins. The microarray was retrieved from −80 °C storage and blocked with bovine serum albumin (BSA, 9048-46-8, Sigma) for 1.5 h. Subsequently, it was incubated with plasma samples at a 1:1000-fold ratio for 1 h. After washing with 0.1% phosphate buffered solution-tween 20, the plates were incubated with Alexa fluor 647 goat anti-human IgG (109-605-003, Jackson) (diluted in 5% BSA) and scanned using GenePix 4300A (141095, Molecular Devices) with a 635 nm excitation laser. The signal intensities of lgG for each protein were quantified using GenePix Pro v.6.0 software (Molecular Devices) (https://www.moleculardevices.com/products/additional-products/genepix-microarray-systems-scanners). In the verification phase, candidate AAbs identified during the discovery phase were selected and printed onto 2 × 7 sub-arrays for an aNSCLC-focused microarray. The experimental procedures of the aNSCLC-focused microarray were similar to the high-density microarray, except for blocking and dilution buffer, which was 3% BSA.

#### ELISA for AAbs Validation

Ten recombinant candidate proteins, including transporter associated with antigen processing binding protein (TAPBP), protein phosphatase 6 regulatory subunit 2 (PPP6R2), mediator complex subunit 22 (MED22), mitotic arrest deficient 2 like 1 (MAD2L1), telomerase regulation-associated protein (SCYL1), dexh-box helicase 29 (DHX29), large tumor suppressor kinase 1 (LATS1), N-Myc downstream regulated 1 (NDRG1), BCL10 immune signaling adaptor (BCL10), and cellular tumor antigen p53 (TP53) with glutathione-S-transferase tags were procured from CDI laboratory, and myc-associated factor X (MAX, #AP89669, SAB) protein was purified from the yeast with histidine 6 tag. However, synaptojanin 2 (SYNJ2), cytochrome p450 family 11 subfamily B member 1 (CYP11B1), phosphatidylinositol-4-phosphate 3-kinase catalytic subunit type 2 alpha (PIK3C2A), and MIB E3 ubiquitin protein ligase 2 (MIB2) proteins were not synthesized successfully. The experimental procedure has been described (25, 28). Briefly, the proteins were diluted into a concentration of 1 ng/μl and added to plates for overnight incubation at 4 °C. Subsequently, the plates were blocked with 5% milk for 2 h. Plasma samples (diluted at 1:300 ratio) were added and incubated for 1 h. Each plasma sample was processed by preparing parallel duplicate wells, with every plate incorporating anti-glutathione-S-transferase-tag rabbit antibody (ab184804, Abcam) at a 1:300 dilution as a positive control, and blank duplicates with milk as the negative control. After three washes, HRP-labeled goat anti-human lgG secondary antibodies (109-035-088, Jackson) diluted 1:8000 was added. Finally, 75 μl of tetramethyl benzidine (abs9178, absin) was added to each well and reacted for 25 min. The plates were immediately detected at a wavelength of 450 nm for OD450 values after termination by the addition of sulfuric acid (abs9191, absin) using Multiskan Go microplate reader (51119200, Thermo Fisher Scientific) and SkanIt Software for Microplate Readers DDE, ver. 5.0.0.42 (Thermo Fisher Scientific) (https://www.thermofisher.cn/order/catalog/product/5187149).

#### Immunohistochemistry Validation

Following deparaffinization and antigen retrieval, FFPE samples underwent incubation with a 3% hydrogen peroxide solution to block endogenous peroxidase activity. Rabbit serum was sealed and then FFPE samples were sealed and incubated with primary antibodies, specifically a 1:1000 dilution for rabbit anti-human MAX IgG antibody (ab101271, Abcam). Then, add a 1:200 dilution of HRP-labeled goat anti-rabbit IgG secondary antibody (GB23303, Servicebio) and incubated for 1 h. Color development was achieved by adding diaminobenzidine (G1212, Servicebio), and cell nuclei were counterstained with hematoxylin (G1004, Servicebio). The results were interpreted using a white light microscope (E100, Nikon), and proteins were quantified using CaseViewer 2.4 (3DHISTECH) (https://www.3dhistech.com/solutions/caseviewer/) software. All patients have H&E staining, and cancer lesions were reviewed and verified by pathologists.

#### mRNA Validation in Publicly Available Datasets

Two gene expression omnibus datasets (http://www.ncbi.nlm.nih.gov/geo) were obtained for RNA-seq data. GSE135222 (26) included 27 NSCLC tissue samples and GSE126044 (27) included 16 NSCLC tissue samples and were all collected before ICIs treatment. Complete clinical efficacy information in these datasets, including PFS and efficacy evaluation, were retrieved. Additionally, the Kaplan–Meier Plotter (https://kmplot.com/analysis/) website was utilized to explore the prognostic value of AAbs-targeted mRNA in pan-cancer patients (bladder cancer, glioblastoma, and melanoma), who received anti-PD1 treatment, including pre-treatment samples from 244 patients and on-treatment samples from 30 patients.

#### Statistical Analysis

All statistical analyses were performed using the IBM SPSS Statistics 24 (https://www.ibm.com/support/pages/downloading-ibm-spss-statistics-24), GraphPad Prism 9 (https://www.graphpadchina.com/download.html), and R version 4.2.1 software (https://www.r-project.org/). Prior to differential analyses in the high-density and aNSCLC-focused microarrays, raw intensity data underwent loess transformation and log2 normalization. The “limma” package in R was utilized to identify differential AAbs between responders and non-responders, as well as before and after treatment in the discovery and verification phases based on the normalized intensity. Mann-Whitney U tests were employed for comparisons between responder and non-responder groups in both T0 and T1 time points. Sensitivity, specificity, and receiver operating characteristic curves were computed using the “pROC” and “ROCR” packages. Cluster dynamic changes were calculated using the “ClusterGVis” package in R. The “maxstat” package in R was utilized to determine the optimal cutoff value for high- and low-expression groups for survival analysis in the verification and validation phases. Paired t-tests were used to compare dynamic changes between paired samples in the dynamic cohorts. Protein expression levels were quantified using the IHC Score, which takes into account the intensity and percentage of positive tumor cells, resulting in scores ranging from 0 to 12. Stain intensity was classified as 0 (negative), 1 (weak), 2 (moderate), and 3 (strong), and the percentage of positive cells was scored as 1 (0–25%), 2 (26–50%), 3 (51–75%), and 4 (>75%). Two experienced pathologists independently reviewed all the results. Chi-square or Fischer’s exact tests were conducted to analyze the group percentages. Gene Ontology and Kyoto Encyclopedia of Genes and Genomes enrichment pathway enrichment analyses were performed using Metascape software (https://metascape.org/gp/index.html#/main/step1) and the Sanger plot website (http://www.sangerbox.com) (29). The GSE135222 and GSE126044 datasets were annotated using the GPL16791 platform. Gene set enrichment analyses in lung adenocarcinoma (LUAD) and lung squamous cell carcinoma (LUSC) from The Cancer Genome Atlas datasets were visualized on the CAMOIP database (http://www.camoip.net/). GEPIA (http://gepia.cancer-pku.cn/) was used to compute spearman correlation between the programmed cell death 1 ligand 1 (CD274) mRNA and prognostic markers’ mRNAs. A significance level of *p* < 0.05 (two-tailed) was considered statistically significant for all analyses.

## Results

### Study Design

**Fig. 1:**
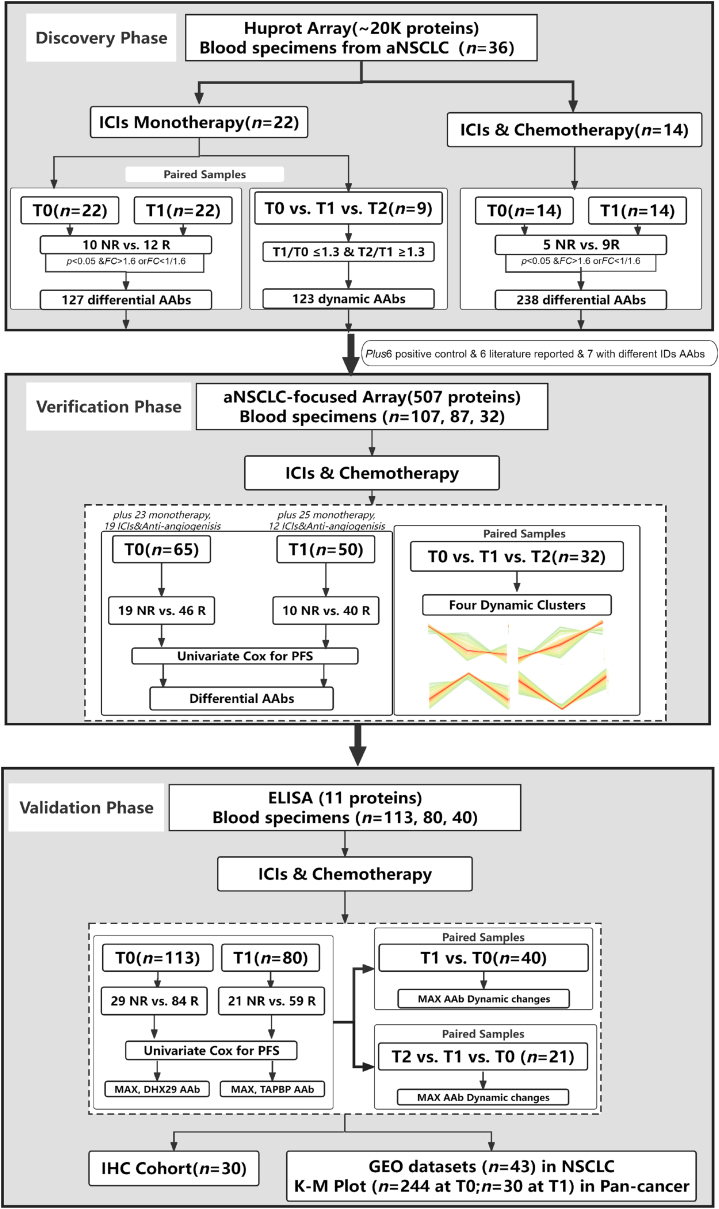
**Flowchart overview of the study.** Prognostic AAbs between responders and non-responders were identified using “limma” package in discovery and verification phases based on normalized intensity, both before and after treatment. Combined with univariate COX regression analysis for PFS, novel prognostic AAbs were identified through a high-density HuProt microarray (*n* = 36 at T0, 36 at T1, and 9 at T2), an aNSCLC-focused microarray (*n* = 107 at T0, 87 at T1, and 32 at T2), and ELISA (*n* = 113 at T0, 80 at T1, and 40 at T2), utilizing paired samples from 36, 49, and 40 patients across three phases. In the discovery phase, a high-density HuProt microarray displaying 21,000 proteins were utilized to identify prognostic AAbs in aNSCLC patients receiving ICIs monotherapy and chemoimmunotherapy. In the verification phase, 507 AAbs were selected for confirmation using an aNSCLC-focused microarray, followed by further validation of 11 AAbs in the validation phase through ELISA, ultimately confirming three AAbs in aNSCLC patients receiving chemoimmunotherapy. Subsequent validation encompassed AAbs-targeted proteins and mRNA in FFPE samples (*n* = 30 at T0), two NSCLC GEO datasets (*n* = 16 at T0 and 27 at T0), and pan-cancer datasets (*n* = 244 at T0 and 30 at T1) treated with ICIs therapy. AAbs, autoantibodies; aNSCLC, advanced non-small cell lung cancer; GEO, gene expression omnibus; ICIs, immune checkpoint inhibitors; IHC, immunohistochemistry; NR, represented non-responder who achieving disease progression within 3 months of treatment; Pan-Cancer, including bladder cancer, glioblastoma, and melanoma; PFS, progression-free survival; R, represented responder who achieving complete remission, partial remission, or stable disease within 3 months of treatment; T0, baseline time point; T1, time point within 3 months treatment; T2, evaluation time point of progression.

The overall study design, as illustrated in Figure 1, consisted of three-phase: discovery, verification, and validation. Each phase included plasma samples at pre-treatment (T0), post-treatment within 3 months of ICIs therapy (T1), and progression evaluation (T2) time points. Prognostic AAbs between responders and non-responders were identified using “limma” package in discovery and verification phases based on normalized intensity, both before and after treatment. Combined with univariate COX regression analysis for PFS, novel prognostic AAbs were identified through a high-density HuProt microarray (*n* = 36 at T0, 36 at T1, and 9 at T2), an aNSCLC-focused microarray (*n* = 107 at T0, 87 at T1, and 32 at T2), and ELISA (*n* = 113 at T0, 80 at T1, and 40 at T2), utilizing paired samples from 36, 49, and 40 patients across three phases. In the validation phase, 52 cases had overlapping pre-treatment blood samples with those from the verification phase, and 32 cases had overlapping post-treatment blood samples with those from the verification phase. In the discovery phase, employing *p* < 0.05 and *FC* > 1.6 or *FC* < 1/1.6 as a threshold for limma differential analysis between responder and non-responder groups in both pre-treatment and on-treatment, 127 and 238 AAbs were selected in ICIs monotherapy and chemoimmunotherapy. In the dynamic cohort, using T1/T0 ≤ 1/1.3 and T2/T1 ≥ 1.3 as a threshold for dynamic AAbs, 123 AAbs were selected, along with six positive control AAbs, six literature reported AAbs, and 7 AAbs having names but with different IDs, totaling 507 AAbs were initially selected for aNSCLC-focused microarray. Subsequently, in the verification phase, 19 AAbs and 27 AAbs (with three AAbs showing overlap) exhibited significant differentials (*p* < 0.05) between responder and non-responder groups in pre-treatment and on-treatment conditions, wherein eight AAbs and nine AAbs (with two AAbs showing overlap) demonstrated discriminatory potential for PFS (*p* < 0.05). Following ELISA tests, 15 AAbs were selected, but due to four proteins’ unsuccessful synthesis, 11 AAbs were tested in the validation phase. Ultimately, three AAbs were validated through a combination of limma differential analysis between responder and non-responder groups and univariate COX regression analysis for progression-free survival. Subsequent validation involved AAbs targeted proteins and mRNA in FFPE samples (*n* = 30 at T0), two NSCLC gene expression omnibus datasets (*n* = 16 at T0 and 27 at T0), and pan-cancer datasets (*n* = 244 at T0 and 30 at T1) treated with ICIs therapy. Detailed clinical characteristics were shown in Table 1 and Supplemental Tables S1–S3.

**Table 1:** Baseline (T0) clinical characteristics of study cohorts (*n* = 204)

Characteristics	Cohorts	Discovery (*n* = 36)		Verification (*n* = 107)			Validation (*n* = 113)
		ICIs	ICIs & Chemo	ICIs	ICIs & Chemo	ICIs & Angio	ICIs & Chemo
	n	22	14	23	65	19	113
Age (year)	Median	60	63.5	62	64	61	63
	Range	32–74	33–71	43–79	50–82	47–78	48–82
Gender	Male	18	12	21	56	11	94
	Female	4	2	2	9	8	19
Smoking	No	7	3	8	12	7	27
	Yes	15	11	15	53	12	86
ECOG	0	8	6	4	32	10	44
	1	14	8	18	19	7	33
	2	0	0	1	5	1	34
	Unknown	0	0	0	9	1	2
Histology	ADC	10	5	8	28	16	50
	SCC	11	8	14	36	3	60
	ASC	1	0	1	1	0	0
	LCC	0	1	0	0	0	3
Stage	Ⅲ	7	3	5	22	4	55
	Ⅳ	15	11	18	43	15	58
Clinical benefit	R	12	9	13	46	10	84
	NR	10	5	10	19	9	29
ICIs line	1	0	3	4	44	6	89
	2	7	5	8	14	8	15
	≥3	15	6	11	7	5	9
*EGFR*	Mutation	2	3	3	10	4	11
	WT	13	6	11	26	13	86
	Unknown	7	5	9	29	2	16

Note: In the validation phase, 52 cases had overlapping pre-treatment blood samples with those from the verification phase, and 32 cases had overlapping post-treatment blood samples with those from the verification phase.

ADC, adenocarcinoma; ASC, adenosquamous carcinoma; ECOG, Eastern Cooperative Oncology Group; EGFR, epidermal growth factor receptor; ICIs, immune checkpoint inhibitors; ICIs & Angio, immune checkpoint inhibitors combined with angiogenic inhibitors; ICIs & Chemo, immune checkpoint inhibitors combined with chemotherapy; LCC, large cell carcinoma; NR, represented non-responder who achieving disease progression within 3 months of treatment; R, represented responder who achieving complete remission, partial remission, or stable disease within 3 months of treatment; SCC, squamous carcinoma.

### aNSCLC AAbs Profiling and Screening for aNSCLC-Focused Microarray

In the discovery phase, 81 plasma samples were obtained from 36 aNSCLC patients, comprising 14 patients receiving chemoimmunotherapy (five patients with two-time points and nine patients with three-time points) and 22 patients undergoing ICIs monotherapy (with two-time points) (Supplemental Fig. S1). After loess normalization, the raw intensity data consistently distributed (Supplemental Fig. S2, *A* and *B*). Each HuProt microarray, as depicted in the schematic diagram (Supplemental Fig. S2), accommodated a single sample, and for inter-experiment reproducibility, duplication on two separate microarrays demonstrated *R* = 0.988 (Supplemental Fig. S2*C*); for intra-experiment reproducibility, with duplicate spots for 21,000 protein spots on each microarray, internal reproducibility was assessed by randomly selecting four samples (including one pre-treatment responder, one pre-treatment non-responder, one post-treatment responder, and one post-treatment non-responder), resulting in an intra-experiment reproducibility of *R* > 0.99 (Supplemental Fig. S2*D*). Vertical clustering of the 21,000 AAbs profiles among the 36 aNSCLC patients revealed four distinct clusters (Supplemental Fig. S3*A*). The lower expression cluster exhibited enrichment in snRNA pseudouridine synthesis, DNA metabolic process, and other pathways (Supplemental Fig. S3*B*), while the highest cluster was associated with the B cell receptor signaling pathway, monocarboxylic acid metabolic process, and other pathways (Supplemental Fig. S3*C*). Samples from the same patient at different time points exhibited a high degree of correlation, whereas the correlation was lower among different individuals, irrespective of whether they received ICIs monotherapy or chemoimmunotherapy (Supplemental Fig. S4, *A* and *B*).

**Fig. 2:**
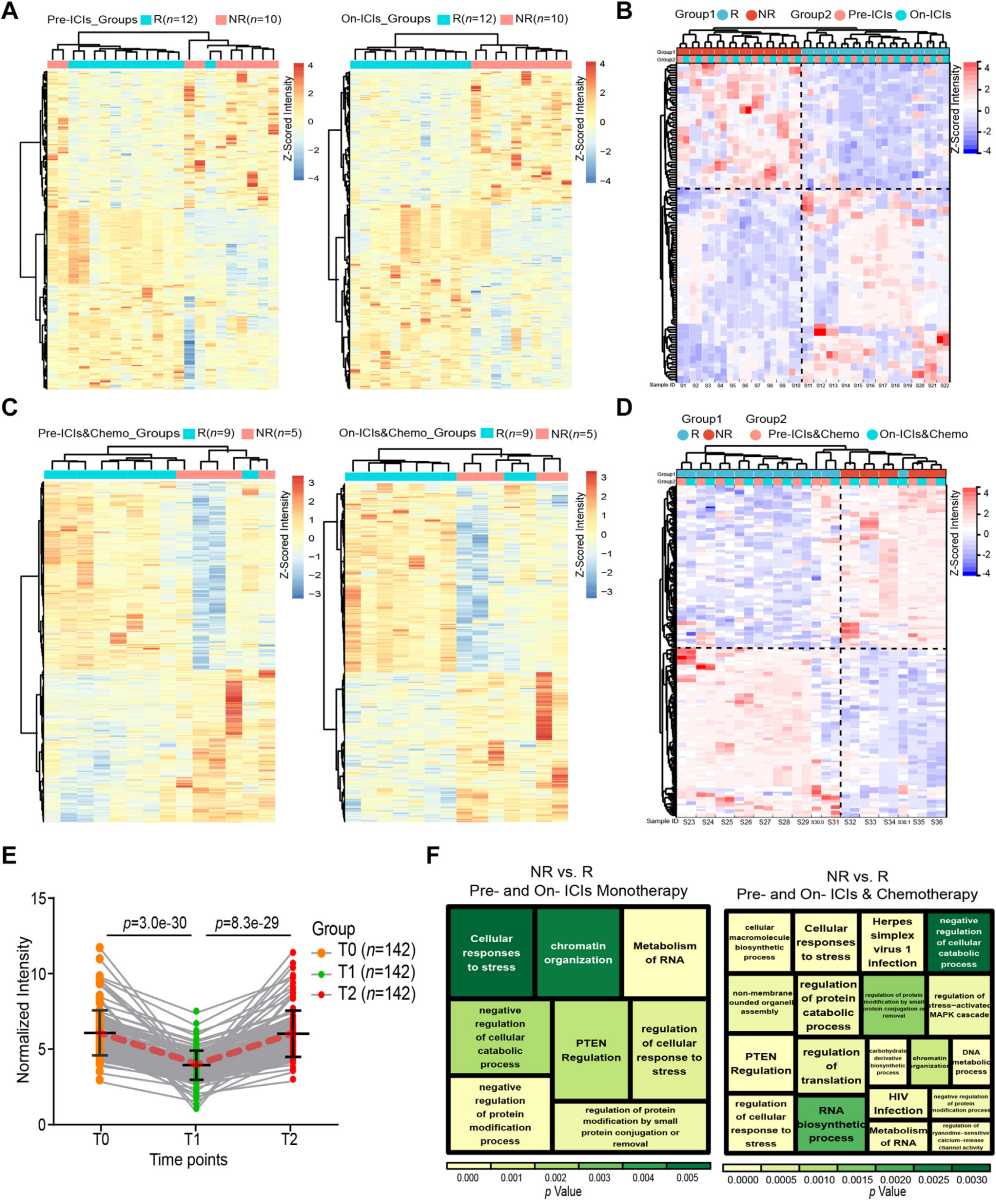
**High-density microarrays for identifying differential AAbs (*p* < 0.05) in the discovery phase (*n* = 36).***A* and *C*, heatmaps illustrating differential AAbs between responders and non-responders pre- and on-ICIs monotherapy (*n* = 22), as well as chemoimmunotherapy (*n* = 14) based on the normalized intensity data (Z-Scored across individuals and *p* < 0.05 and *FC* > 1.6 or *FC* < 1/1.6 as a threshold). *B* and *D*, clustermaps illustrating 127 differential AAbs between responders and non-responders overlapped pre- and on-ICIs monotherapy and overlapped 238 differential AAbs in chemoimmunotherapy based on the normalized intensity data (Z-Scored across individuals and *p* < 0.05 and *FC* > 1.6 or *FC* < 1/1.6 as a threshold). *E*, dynamic 123 prognostic AAbs showed decreased post-treatment (T1/T0 ≤ 1/1.3) and increased during disease progression (T2/T1 ≥ 1.3) in nine patients with three time points (T0, T1, and T2) receiving ICIs monotherapy. *F*, functional enrichment of differential AAbs overlapped pre- and on-ICIs monotherapy and chemoimmunotherapy, respectively. AAbs, autoantibodies; ICIs & Chemo, immune checkpoint inhibitors combined with chemotherapy; ICIs, immune checkpoint inhibitors; NR, represented non-responder who achieving disease progression within 3 months of treatment; R, represented responder who achieving complete remission, partial remission, or stable disease within 3 months of treatment; T0, baseline time point; T1, time point within 3 months treatment; T2, evaluation time point of progression.

Comparing responders (*n* = 12 for ICIs monotherapy and *n* = 9 for chemoimmunotherapy) and non-responders (*n* = 10 for ICIs monotherapy and *n* = 5 for chemoimmunotherapy) at T0 and T1 time points and assessing dynamic changes across three-time points using normalized intensity data, a total of 127 AAbs (Fig. 2, *A* and *B*), 238 AAbs (Fig. 2, *C* and *D*), and 123 AAbs (Fig. 2*E*) (*p* < 0.05) were identified as prognostic AAbs. Utilizing *p* < 0.05 and *FC* > 1.6 or *FC* < 1/1.6 as a threshold for limma differential analysis between responder and non-responder groups in both pre-treatment and on-treatment, 127 and 238 AAbs were selected in ICIs monotherapy and chemoimmunotherapy (Fig. 2, *A*–*D*). Cluster analysis revealed distinct AAb profiling in responder compared to non-responder patients, with individual clustering evident pre- and on-treatment on the whole, while AAbs abundances change for each individual from pre- to on-treatment was not evident (Fig. 2, *B* and *D*). Further comparison of mean intensity values in nine patients with three time points (T0, T1, and T2) receiving ICIs monotherapy indicated that 123 AAbs showed decreased post-treatment (T1/T0 ≦ 1/1.3) and increased during disease progression (T2/T1 ≥ 1.3) (Fig. 2*E*). Subsequently, plus six positive control AAbs, six literature reported AAbs, and seven AAbs having same names but with different IDs, these 507 AAbs were selected for the aNSCLC-focused microarray (Supplemental Table S3). Prognostic AAbs associated with ICIs monotherapy and chemoimmunotherapy were both enriched in RNA metabolism, negative regulation of protein modification processes, and PTEN regulation (Fig. 2*F*).

### Prognostic AAbs Verification for PFS and Dynamic Clusters

In the verification phase, dynamic paired samples were obtained from 49 aNSCLC patients: 17 receiving ICIs monotherapy, 19 undergoing chemoimmunotherapy, and 13 treated with ICIs combined with angiogenesis inhibitor (Supplemental Fig. S5). As depicted in the diagram (Supplemental Fig. S6), each aNSCLC-focused microarray accommodated 14 samples, and across three experimental batches, a single sample was distributed among three microarrays to assess inter-experiment reproducibility (*R* = 0.95, 0.95, 0.99) (Supplemental Fig. S6*A*); additionally, within the same microarray but in different blocks, the reproducibility of the same sample was tested, resulting in intra-experiment reproducibility (*R* = 0.993) (Supplemental Fig. S6*B*); with multi-spot configurations for protein spots on each block, intra-block reproducibility was further calculated, illustrated by randomly selecting one sample to show intra-block reproducibility (*R* = 0.999) (Supplemental Fig. S6*C*). Similarly to the high-density microarray, the AAbs spectrum exhibited strong similarity within the same individual but showed poor similarity between different individuals (Supplemental Fig. S7, *A*–*D*). Among the 507 AAbs on the aNSCLC-focused microarray, PDCD1 AAbs showed elevated levels after treatment in ICIs monotherapy, chemoimmunotherapy, and ICIs combined with angiogenesis inhibitor therapy (Supplemental Fig. S8*A*). PD-L1 expression and PD-1 checkpoint pathway in cancer was enriched across all three treatments, while the T cell receptor signaling pathway was enriched in ICIs monotherapy and ICIs combined with angiogenesis inhibitor therapy, and the vascular endothelial growth factor signaling pathway was observed in ICIs combined with angiogenesis inhibitor therapy (Supplemental Fig. S8*B*). Specifically, the degree of elevation of PDCD1 AAb varied among different treatments, with ICIs monotherapy showing the highest fold change (1.72-fold changes, *p* < 0.01), followed by ICIs combined with angiogenesis inhibitor therapy (1.44-fold changes, *p* < 0.001), and chemoimmunotherapy (1.37-fold changes, *p* < 0.01). The same results were also observed in the paired dynamic changes in PDCD1 AAb (Supplemental Fig. S8, *C*–*G*).

**Fig. 3:**
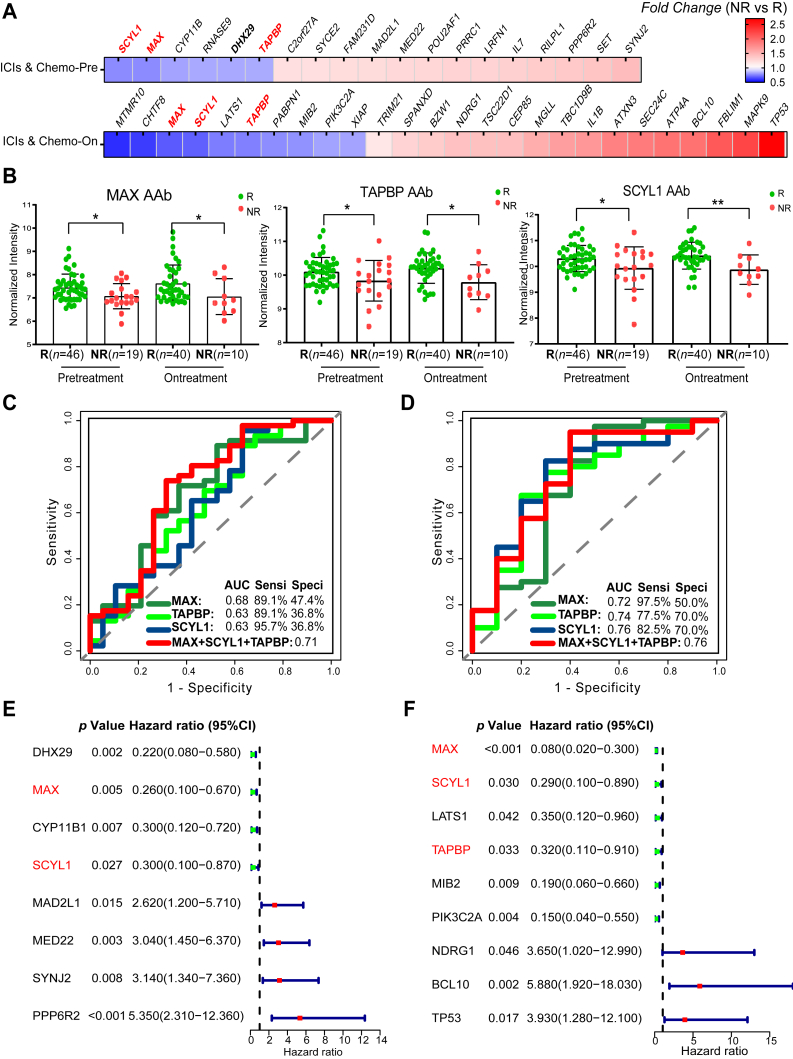
**Candidate prognostic AAbs for pre- and on-chemoimmunotherapy in the verification phase (*n* = 65 pre-treatment, 50 on-treatment).***A*, fold-changes of differential AAbs between responders and non-responders pre- and on-chemoimmunotherapy (*p* < 0.05) by “limma” analysis. *B*, comparison of three prognostic AAbs (*p* < 0.05) between responders and non-responders pre- and on-chemoimmunotherapy. *C* and *D*, ROC curves of the three AAbs (MAX, TAPBP, and SCYL1 AAbs) for predicting responder and non-responder patients pre- and on-chemoimmunotherapy. *E* and *F*, univariate Cox regression for PFS pre- and on-chemoimmunotherapy. ∗*p* < 0.05, ∗∗*p* < 0.01, ns: not significant. AAbs, autoantibodies; CI, confidence interval; FC, fold changes; HR, hazard risk; ICIs & Chemo, immune checkpoint inhibitors combined with chemotherapy; NR, represented non-responder who achieving disease progression within 3 months of treatment; PFS, progression-free survival; R, represented responder who achieving complete remission, partial remission, or stable disease within 3 months of treatment; ROC, receiver operating characteristic; TAPBP, transporter associated with antigen processing binding protein.

**Fig. 4:**
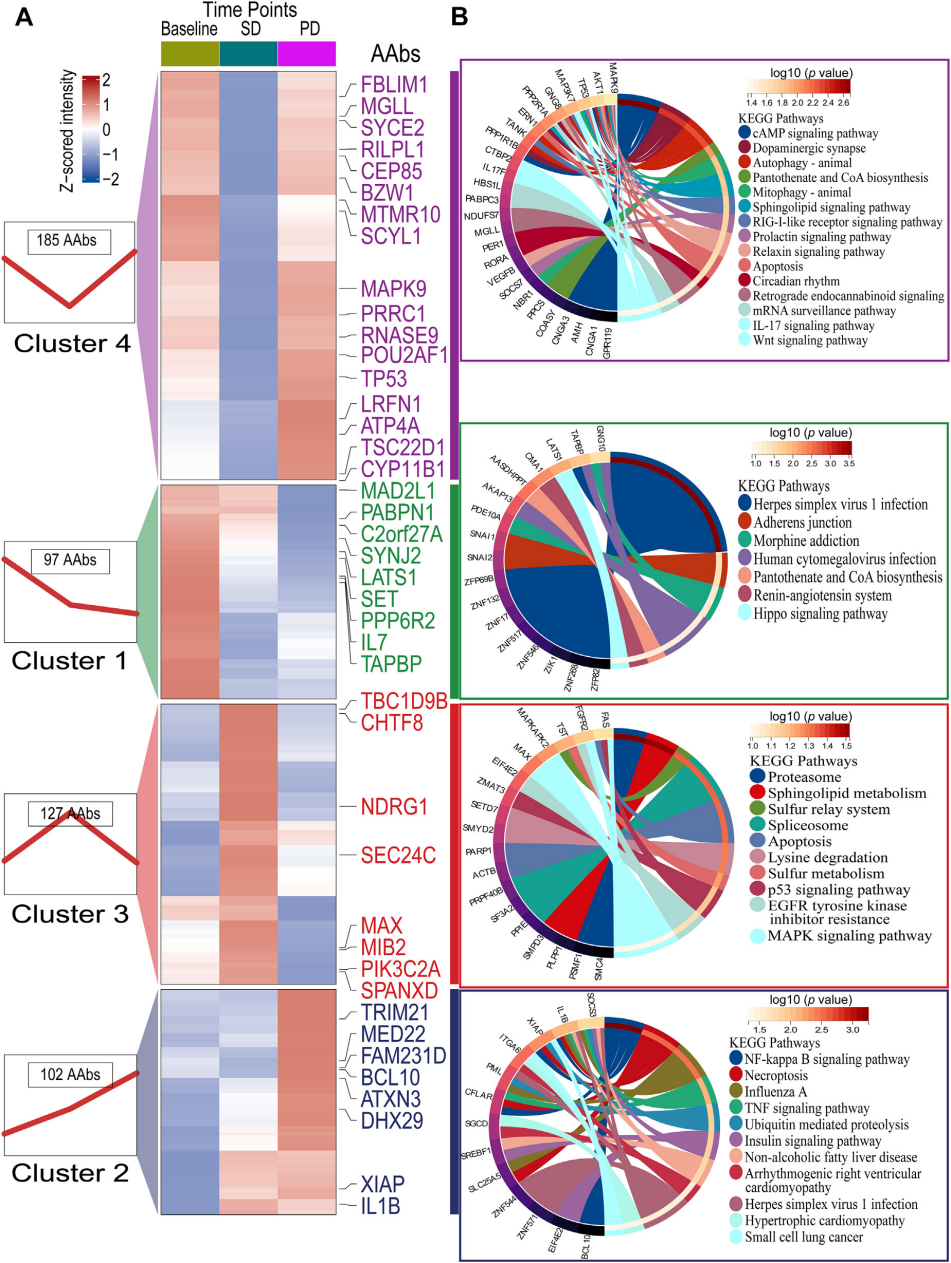
**Clusters and functional enrichment of AAbs from baseline, SD, to PD in the dynamic verification phase.** (*n* = 32). *A*, clusters of 507 AAbs from baseline, SD, to PD. The normalized intensity were Z-Scored across individuals. *B*, functional enrichment of 507 AAbs from baseline, SD, to PD in four clusters. AAbs, autoantibodies; KEGG, Kyoto Encyclopedia of Genes And Genomes; PD, progression disease; SD, steady disease.

In the comparison between responders (*n* = 46 at T0, *n* = 40 at T1) and non-responders (*n* = 19 at T0, *n* = 10 at T1) receiving chemoimmunotherapy by limma analysis, a total of 19 AAbs and 27 AAbs were selected (*p* < 0.05), of which three AAbs (MAX, TAPBP, and SCYL1) exhibited overlap between T0 and T1 time points, and the fold changes of these differential AAbs were consistent with the findings from the discovery phase (Fig. 3, *A* and *B* and Supplemental Table S4). MAX AAb exhibited 89.1% sensitivity and 47.4% specificity before chemoimmunotherapy and 97.5% sensitivity and 50.0% specificity after chemoimmunotherapy (Fig. 3, *C* and *D*). TAPBP and SCYL1 AAbs demonstrated sensitivity of 89.1% and 95.7% before chemoimmunotherapy, 77.5% and 82.5% after chemoimmunotherapy, with specificity of 36.8% and 36.8% before chemoimmunotherapy, and 70.0% and 70.0% after chemoimmunotherapy (Fig. 3, *C* and *D*). Logistic regression models for responders and non-responders revealed that the combination of MAX, SCYL1, and TAPBP AAbs had superior performance, with an area under the curve of 0.71 before chemoimmunotherapy (Fig. 3*C*) and 0.76 after chemoimmunotherapy (Fig. 3*D*). Univariate Cox regression analyses identified eight AAbs (DHX29, MAX, CYP11B1, SCYL1, MAD2L1, MED22, SYNJ2, and PPP6R2) at baseline and nine AAbs (MAX, SCYL1, LATS1, TAPBP, MIB2, PIK3C2A, NDRG1, BCL10, and TP53) post-treatment predictive of PFS (*p* < 0.05) (Fig. 3, *E* and *F* and Supplemental Table S5), among which MAX and SCYL1 showed predictive value both pre-treatment and on-treatment. Additionally, 11 AAbs (MAX, SCYL1, TAPBP, PPP6R2, MED22, MAD2L1, LATS1, NDRG1, BCL10, TP53, and DHX29) predictive of responses pre- or on-chemoimmunotherapy were selected as candidate AAbs for validation *via* ELISA. A dynamic analysis categorized the 507 AAbs into four clusters based on mean intensity value changes in T0 (baseline), T1 (stable disease), and T2 (disease progression), revealing distinct longitudinal patterns (*n* = 32): Cluster 1 with constantly decreasing AAbs, cluster 2 with constantly increasing AAbs, cluster 3 with elevated AAbs during diseases stability followed by a sharp decline during disease progression, and cluster 4 with declined AAbs during diseases stability followed by a sharp increase during disease progression (Fig. 4, Supplemental Table S6). Prognostic TAPBP, PPP6R2, and LATS1 AAbs were assigned to cluster 1, MED22, DHX29, and BCL10 AAbs were assigned to cluster 2, MAD2L1, NDRG1, and MAX AAbs were assigned to cluster 3, while SCYL1 and TP53 AAbs were assigned to cluster 4 (Supplemental Table S6).

Furthermore, a summary of AAbs associated with therapeutic and prognostic efficacy in NSCLC, spanning patients undergoing surgery, chemotherapy, radiotherapy, or immunotherapy, was compiled (Supplemental Table S7). Comparative analysis of AAbs documented in NSCLC was performed between the responder and non-responder groups during the discovery (36 AAbs) and verification (7 AAbs) phases before and after ICIs therapy, revealing a higher level of protein phosphatase 2 scaffold subunit alpha (PPP2R1A) AAb (30) in non-responders than responders (*p* < 0.05), while TRIM21 AAb (23) having higher level in responders than non-responders (*p* < 0.05) (Supplemental Fig. S9, Supplemental Table S8) in the discovery phase pretreatment; however, no significant differences (*p* > 0.05) were observed in the verification phase pretreatment.

### Prognostic AAbs Validation and Different Kinetics in Responders and Non-responders

**Fig. 5:**
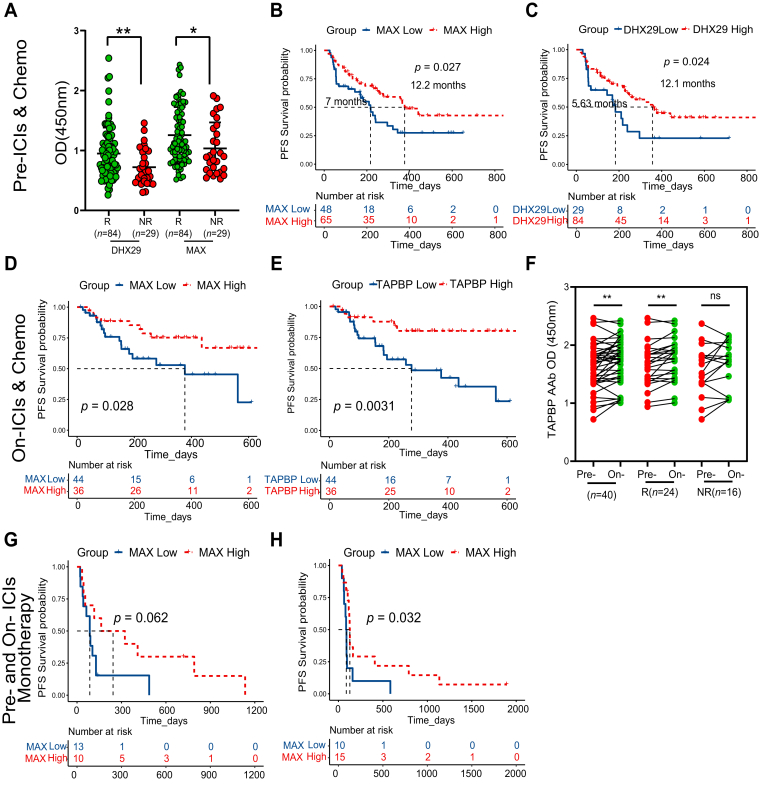
**Performance of three AAbs in discriminating responders and non-responders and predicting PFS pre- and on-chemoimmunotherapy (*n* = 113 pre-treatment, 80 on-treatment) in the validation phase and ICIs monotherapy (*n* = 23 pre-treatment, 25 on-treatment) in the verification phase.***A*, differential analyses of DHX29 and MAX AAbs between responders and non-responders. *B* and *C*, DHX29 and MAX AAbs in predicting PFS pre-chemoimmunotherapy. *D* and *E*, MAX and TAPBP AAbs in predicting PFS on-chemoimmunotherapy. *F*, dynamic changes of TAPBP AAb in validation cohort. *G* and *H*, MAX AAb in predicting PFS pre- and on- ICIs monotherapy in verification cohort. ∗*p* < 0.05, ∗∗*p* < 0.01, ns: not significant. AAbs, autoantibodies; ICIs & Chemo, immune checkpoint inhibitors combined with chemotherapy; NR, represented non-responder who achieving disease progression within 3 months of treatment; OD, optical density; PFS, progression-free survival; R, represented responder who achieving complete remission, partial remission, or stable disease within 3 months of treatment; TAPBP, transporter associated with antigen processing binding protein.

**Fig. 6:**
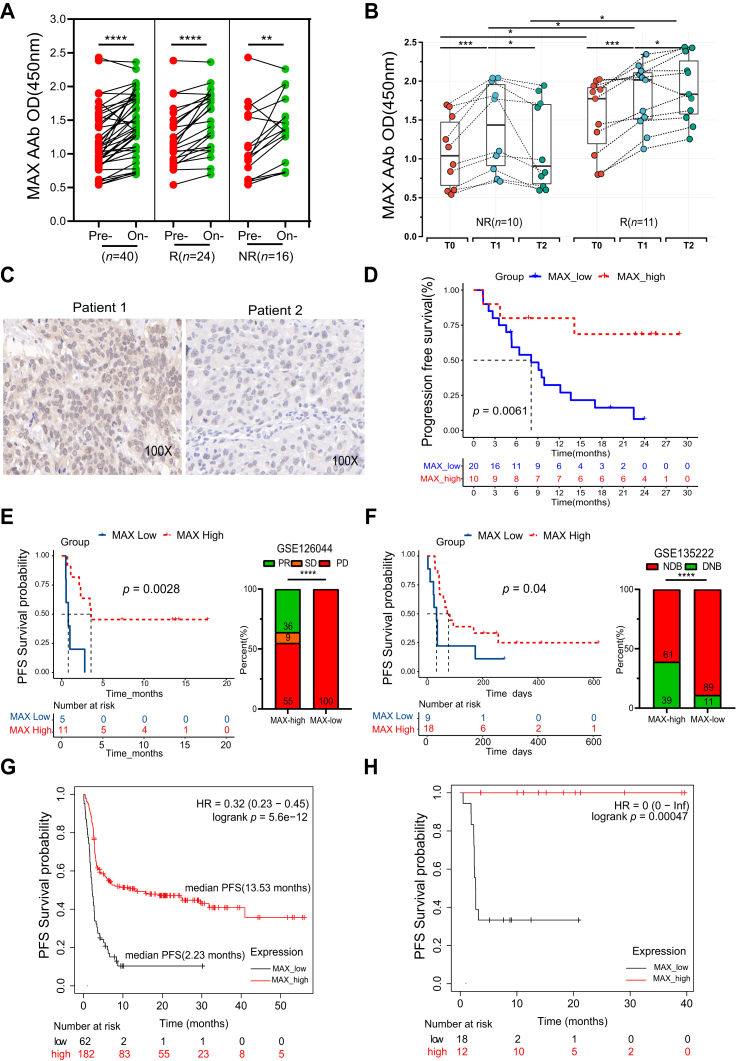
**Dynamic changes of MAX AAb in chemoimmunotherapy in the validation phase (*n* = 40, 21) and prognostic value of MAX protein and mRNA in IHC cohort (*n* = 30, 100X) and NSCLC datasets (*n* = 16, 27, 244 and 30).***A* and *B*, changes of MAX AAb at two-time points and three-time points pre- and on-chemoimmunotherapy in responders and non-responders, respectively. *C*, representative IHCs staining of MAX in patient 1 (PFS = 24.9 months) and patient 2 (PFS = 2.67 months). *D*, Kaplan–Meier survival curves of PFS grouped by the MAX expression. *E* and *F*, MAX mRNA in predicting PFS and differential analyses in NDB, DNB or PR, SD, PD groups in GSE135222 and GSE126044, respectively. *G* and *H*, MAX mRNA in predicting PFS pre- and on-immunotherapy in the K-M plot database. ∗*p* < 0.05, ∗∗*p* < 0.01, ∗∗∗*p* < 0.001, ∗∗∗∗*p* < 0.0001, ns: not significant. AAb, autoantibody; DNB, durable clinical benefit; ICIs, immune checkpoint inhibitors; IHC, immunohistochemistry; NDB, no durable benefit; NSCLC, non small-cell lung cancer; PD, progression disease; PFS, progression-free survival; PR, partial remission; SD, steady disease; T0, baseline time point; T1, time point within 3 months treatment; T2, evaluation time point of progression.

In the ELISA phase, as illustrated in the diagram (Supplemental Fig. S10), initial experiments involving 29 samples were conducted to assess inter-experiment reproducibility (*R* = 0.90) (Supplemental Fig. S10*A*); each plate incorporated duplicate wells for every sample, further evaluating inter-experiment reproducibility (*R* = 0.99) (Supplemental Fig. S10*B*); for both intra-experiment and inter-experiment reproducibility, coefficients of variation (CV) were calculated, where CV, defined as the SD (σ) of duplicate wells divided by the mean (μ) of duplicate wells, quantifies reproducibility, with larger CV values indicating poorer reproducibility; the experiment's CV values remained below 3.1%, ensuring reliable reproducibility of mean OD values (Supplemental Fig. S10, *C* and *D*). Among the 11 candidate AAbs, MAX and DHX29 AAbs exhibited significant differences (*p* < 0.05) (Supplemental Fig. S11, 5*A* and Supplemental Table S9) between responders (*n* = 84 at T0) and non-responders (*n* = 29 at T0) and were correlated with superior PFS (*p* < 0.05) (Fig. 5, *B* and *C*) pre-chemoimmunotherapy. MAX AAb displayed the capability to differentiate responders from non-responders with 66.7% sensitivity and 62.0% specificity (Supplemental Fig. S12*A*) before chemoimmunotherapy and 52.5% sensitivity and 76.2% specificity (Supplemental Fig. S12*B*) after chemoimmunotherapy. DHX29 AAb exhibited 84.5% sensitivity before chemoimmunotherapy, while TAPBP AAb after chemoimmunotherapy showed 50.8% sensitivity; the specificities were 44.8% and 66.7%, respectively (Supplemental Fig. S12). Logistic regression models for responders and non-responders showed that the MAX and DHX29 combination had an area under the curve of 0.67 before chemoimmunotherapy (Supplemental Fig. S12*A*) and 0.59 after chemoimmunotherapy when combining of MAX and TAPBP (Supplemental Fig. S12*B*). The optimal cutoff values to divide the high- and low-expression groups were determined as 1.00 OD for MAX, 0.59 OD for DHX29, and 1.85 OD for TAPBP using the “maxstat” package in R software. Furthermore, MAX and TAPBP AAbs showed predictive value for PFS on-chemoimmunotherapy (*p* < 0.05) and MAX AAb demonstrated prognostic value for PFS both pre- and on- ICIs monotherapy in the verification cohort (*p* < 0.05) (Fig. 5, *D*, *E*, *G* and *H*). TAPBP AAb showed an increasing trend on-chemoimmunotherapy *versus* pre-chemoimmunotherapy (*p* < 0.01), especially in responders (*p* < 0.01), while no significant difference was observed in non-responders (*p* > 0.05) (Fig. 5*F*). As depicted in Figure 6*A*, MAX AAb levels exhibited elevation during treatment compared to pre-treatment in both responders and non-responders (*p* < 0.01), exhibiting a rise followed by a decrease in non-responders (*p* < 0.05) and remaining constantly elevated in responders (*p* < 0.05) (Fig. 6*B*, Supplemental Table S10).

### MAX Protein and mRNA can Predict PFS and Functional Enrichment for MAX, TAPBP, and DHX29

MAX protein expression was assessed in 30 NSCLC FFPE samples. Representative examples of MAX expression ranging from negative, weak, moderate to strong were shown in Supplemental Fig. S13, and IHC staining of MAX in two representative patients with different PFS was illustrated in Figure 6*C* (patient 1 with long PFS of 24.9 months, stage III, and patient 2 with short PFS of 2.67 months, stage III). MAX protein expression showed predictive value for PFS (*p* < 0.05) (Fig. 6*D*). Additionally, high MAX mRNA expression correlated with improved prognosis in GSE126044 and GSE135222 datasets, manifesting longer PFS (*p* < 0.05), fewer progression events (*p* < 0.0001), and increased durable clinical benefit events (*p* < 0.0001) (Fig. 6, *E* and *F* and Supplemental Table S11). Additionally, in pan-cancer datasets (Kaplan–Meier plotter) comprising bladder cancer, glioblastoma, and melanoma patients treated with anti-PD1 therapy, MAX (*p* < 0.001) (Fig. 6, *G* and *H*) and DHX29 mRNA (*p* < 0.05) (Supplemental Fig. S14*A* and *B*) also exhibited predictive potential for PFS in both the pre-treatment and on-treatment, while TAPBP mRNA also showed predictive value for PFS on-treatment (*p* < 0.05) (Supplemental Fig. S13*C*).

**Fig. 7:**
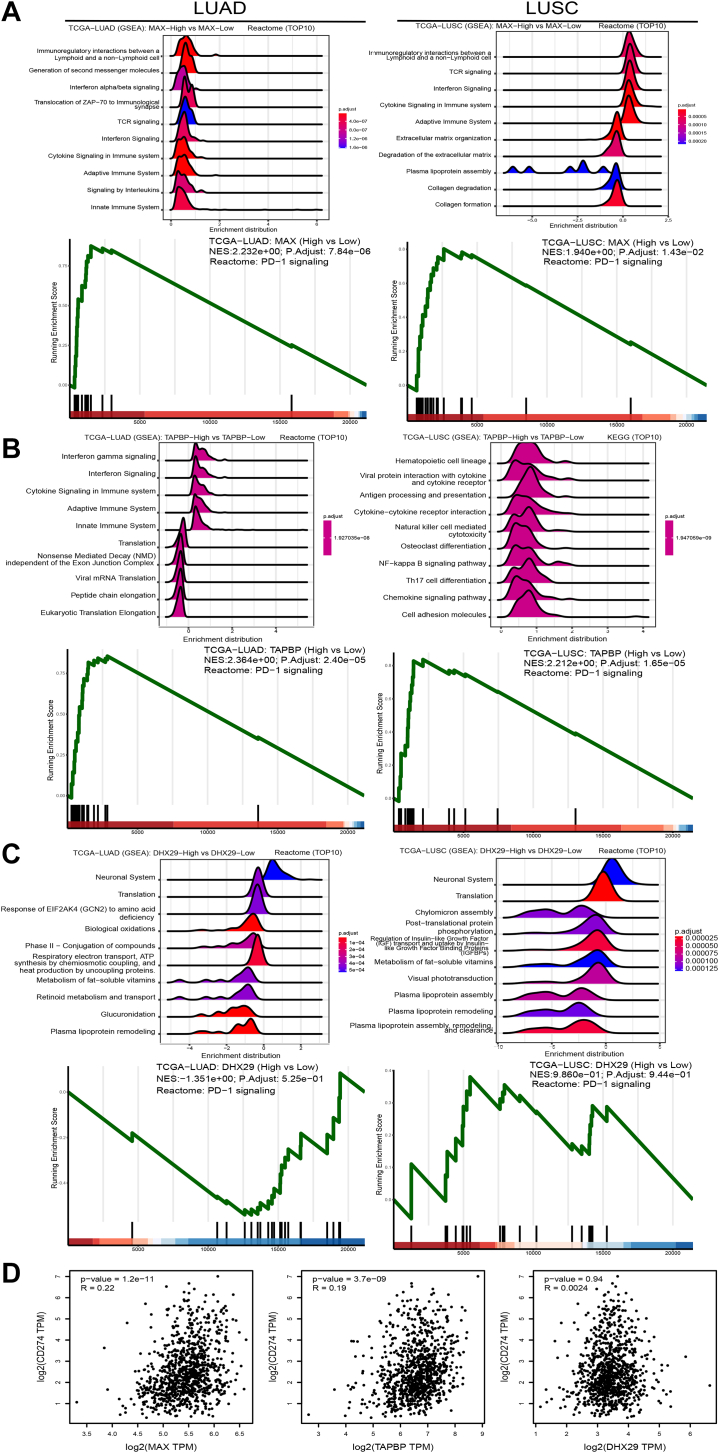
**GSEA analyses for MAX, TAPBP, and DHX29 in LUAD and LUSC TCGA datasets.***A*–*C*, GSEA and Reactome PD-1 signaling enrichment between high and low groups of MAX, TAPBP, and DHX29 in LUAD and LUSC. *D*, correlation between MAX, TAPBP, and DHX29 mRNAs and CD274 (PD-L1) mRNAs. GSEA, gene set enrichment analysis; LUAD, lung adenocarcinoma; LUSC, lung squamous cell carcinoma; PD-1, programmed death-1; PD-L1, programmed death ligand-1; TAPBP, transporter associated with antigen processing binding protein; TCGA, the cancer genome atlas.

Gene set enrichment analysis for MAX and TAPBP in LUAD and LUSC identified enrichment of the high-expression groups in interferon signaling, cytokine signaling in immune system, cytokine signaling in immune system, and adaptive immune system pathways, as well as an enrichment of PD-1 signaling in both MAX and TAPBP high-expression groups across both LUAD and LUSC (Fig. 7, *A* and *B*). DHX29 was focused the on neuronal system, translation, metabolism of fat-soluble vitamins, plasma lipoprotein remodeling, and it was not correlated with PD-1 signaling (Fig. 7*C*). MAX and TAPBP were positively correlated with CD274 (encoding PD-L1) (*r* = 0.22 and 0.19, *p* < 0.0001), while DHX29 was not significantly correlated with CD274 (Fig. 7*D*).

## Discussion

To our knowledge, this study represents the most extensive and comprehensive screening of 21,000 AAbs data in aNSCLC patients receiving ICIs monotherapy and chemoimmunotherapy, assessing pre- and post-treatment dynamics. Unlike previous studies that focused on a limited number of AAbs, our study pursued a systematic approach to screen, identify, and validate prognostic AAbs relevant to early progression and PFS in aNSCLC patients receiving ICIs. Our study encompassed a de-novo screening and multi-stage validation using a proteomic test based on AAbs. In clinical practice, our test holds potential as a prognostic and monitoring tool for chemoimmunotherapy, assisting in identifying patients with a poor prognosis and dynamically assessing treatment efficacy, facilitating timely detection of disease progression.

Chemoimmunotherapy has been flourishing with significant clinical benefits in aNSCLC. But most treated patients inevitably experience resistance, making it necessary for predictive and prognostic biomarkers to monitor treatment efficacy. Current markers such as tumor tissue PD-L1 detection, TMB, and MSI-H/dMMR face limitations due to varied detection platforms, tissue acquisition challenges, and difficulties in dynamic monitoring (8, 9, 10). To address these limitations, there is a growing focus on the development of liquid biopsies to analyze circulating tumor DNA, protein, and AAbs in peripheral blood as potential biomarkers. Tumor-associated AAbs have shown promise in CTLA-4 and PD-1 monoclonal antibody therapies for melanoma and lung cancer, exemplified by the survival association of NY-ESO-1 AAb in these patients. Additionally, AAbs such as antinuclear, anti-thyroglobulin, anti-thyroid peroxidase, p53, BRCA2, HUD, and TRIM21 have been identified as predictors of PFS and overall survival (OS) in NSCLC undergoing ICIs therapy (20, 21, 22, 23). However, these aforementioned studies have some limitations, including small sample size, insufficient de-novo studies, absence of multiple validation cohorts, limited exploration of chemoimmunotherapy, and insufficient real-time dynamic monitoring of therapeutic efficacy. Studies shows that chemotherapy seems to offset predictors of immunotherapy to a certain extent, and the adverse prognostic factors of chemoimmunotherapy may actually be adverse prognostic factors of chemotherapy and tumor characteristics (31).

The major finding of our study is the identification of longitudinal prognostic AAbs in aNSCLC receiving chemoimmunotherapy, revealing two pre-therapy AAbs (MAX and DHX29) and two on-therapy AAbs (MAX and TAPBP) in plasma samples that can predict early progression and PFS. Similar to prior studies in this field searching for predictive AAbs in NSCLC treated with ICIs, such as NY-ESO-1 (22, 23), these three AAbs (MAX, DHX29, and TAPBP) exhibit positive correlations with favorable outcomes, including fewer progression events, longer PFS, and OS. It is noteworthy that MAX AAb demonstrated predictive value both before and after treatment, displaying increased levels in responders and decreased levels in non-responders, while TAPBP AAb levels rise solely in responders but showed no significant change in non-responders. The differential AAbs kinetics between responder and non-responder suggest their potential as markers for dynamically monitoring treatment efficacy. Furthermore, the analysis of MAX, DHX29, and TAPBP mRNA in pre-treatment and on-treatment analyses of pan-cancer patients treated with anti-PD1 therapy exhibited predictive value in discriminating PFS. It remains to explore their prognostic potential in other cancer types receiving immunotherapy. Furthermore, MAX protein levels can discriminate PFS in pre-treatment NSCLC patients, further supporting the prognostic value of MAX. Collectively, these findings robustly support the ability of MAX, DHX29, and TAPBP AAbs to distinguish patients prone to developing immunotherapy resistance and predict their survival benefits. In the cross-comparison of reported AAbs associated with therapeutic efficacy in NSCLC and our AAbs during the discovery and verification phases, the PPP2R1A AAb exhibited higher levels in non-responders than responders, aligning with Akshay J.'s (30) findings that anti-PPP2R1A AAbs independently predict poor survival in NSCLC after surgery. Conversely, the TRIM21 AAb demonstrated lower levels in non-responders than responders, contradicting prior report (23) indicating that AAbs-positive patients have superior PFS than AAbs-negative patients. A potential explanation for this discrepancy lies in the literature's focus on assessing the positivity or negativity of five AAbs, including TRIM21, without evaluating the ability of TRIM21 AAbs themselves as prognostic factors for PFS through survival analysis, thereby introducing potential bias into the results.

Moreover, the consistency of individuals' autoantibody profiles pre- and post-treatment, alongside notable inter-individual variability observed in paired samples, aligns with previous research conducted by Abdellah Tebani (32) and Maja Neiman (33), which revealed that AAb profiles exhibit high variability between individuals while remaining stable over time. The high heterogeneity among individual AAb profiles poses challenges in the identification of prognostic biomarkers. The significant consistency of AAb profiles and their inter-individual variation emphasizes the importance of large-scale, multi-phase investigations to discover and validate prognostic AAbs.

Another intriguing aspect of interest is the longitudinal changes of PDCD1 AAb during immunotherapy. We observed an elevation in PDCD1 AAb levels following anti-PD1 therapy, whether administered as monotherapy or in combination with chemotherapy or angiogenesis inhibitors. The fold changes in PDCD1 AAbs from high to low were as follows: anti-PD1 monotherapy, anti-PD1 with angiogenesis inhibitors therapy, and anti-PD1 with chemotherapy. This pattern can be attributed to the inhibitory effect of chemotherapy on bone marrow hematopoietic function and B lymphocyte subsets (34). The clearance rate of monoantibody drugs, including nivolumab and pembrolizumab, has served as a predictive indicator for efficacy in NSCLC, melanoma, and renal cell carcinoma (35, 36, 37). Moreover, in anti-PD1 monotherapy, we observed elevated PDCD1 AAb levels in responders and no significant changes in non-responders. This discrepancy may be attributed to variations in the metabolic rates of anti-PD1 monoantibody among different patients, with responders exhibiting slower metabolism rates than non-responders, suggesting the potential of PDCD1 AAb as an indicator of anti-PD1 monoantibody metabolic rates.

Specifically speaking, MAX belongs to the helix-loop-helix leucine zipper family of transcription factors and functions as a cofactor for DNA binding, specifically interacting with the MYC family of oncoproteins and MYC antagonists, exerting influence on proliferation, differentiation, and apoptosis. MAX is known as a tumor suppressor in small-cell lung cancer (38, 39, 40). In NSCLC, MAX binds to the PD-L1 gene promoter, promoting PD-L1 transcription, thereby showing a positive correlation with PD-L1 expression and CD8^+^ T cell infiltration in lung tumors (39). Our study highlights the association between high levels of MAX mRNA, protein, or AAbs and favorable outcomes, corroborated by the positive correlation observed between MAX mRNA and PD-L1 mRNA. During treatment, as immunotherapy reshapes the immune microenvironment, we observed an elevation in MAX AAb levels, particularly in responders, suggesting the potential of MAX AAbs as a marker for monitoring chemoimmunotherapy efficacy.

TAPBP belongs to the immunoglobulin superfamily and plays a crucial role in mediating the interaction between newly assembled major histocompatibility complex class 1 and antigen processing-associated transporter. TAPBP is essential for effective CD8^+^ T cell responses to tumors and anti-cancer immune surveillance, as the presentation of major histocompatibility complex class-I antigens on cancer cells triggers the recognition and destruction of cancer cells by cytotoxic T cells (CTLs) (41). Downregulation of TAPBP (tapasin) protein expression has been observed in various cancers as an immune escape mechanism. The absence of tapasin hampers the antigen processing of tumor-associated antigens and leads to evasion of tumor-associated antigen–specific CTLs recognition, leading to poorer prognosis (such as colorectal cancer, glioblastoma, and NSCLC), tumor progression and metastasis, and lower CD8^+^T tumor-infiltrating lymphocytes (42, 43, 44, 45, 46). In NSCLC, elevated TAPBP gene expression in peripheral blood has been associated with improved disease-free survival, and tapasin expression in tumor samples has been positively correlated with better OS (44, 47). In melanoma patients, high tapasin expression has been associated with favorable responses to immunotherapy (48). Given that elevated levels of TAPBP AAb after treatment have shown prognostic value in immunotherapy and the positive association between TAPBP mRNA and PD-L1 mRNA, we propose that the increase in TAPBP AAb levels in responders after reshaping the tumor microenvironment is indicative of superior treatment outcomes.

DHX29 plays a crucial role in efficient 48S complex formation on mRNAs, associated with the 40S ribosomal subunit. DHX29 plays a role in promoting translation initiation, cell proliferation, and tumorigenesis (49). DHX29 serves as a cytosolic nucleic acid cosensor, crucial in recognizing cytoplasmic nucleic acids. Knocking down DHX29 reduces the cell's ability to produce type I interferon and interleukin-6 in response to nucleic acids and viruses. DHX29 activates the RIG-I/MAVS-dependent signaling pathway, making it important for immune responses and potential drug/vaccine design against viral infections (50). RIG-I activation is critical for responsiveness to checkpoint blockade, and RIG-I activating immunostimulatory RNA boosts the efficacy of anti-cancer vaccines and synergizes with immune checkpoint blockade (51, 52). However, there have been limited studies on the function of DHX29 in immunotherapy, and the mechanism underlying the prognostic value of DHX29 AAbs in immunotherapy remains unexplored.

While our study demonstrates promising results, it is currently in the research phase, and its implementation in routine clinical practice requires further validation and consideration of various factors. Further investigations are needed to assess the accuracy and reliability of our assay in treatment decision-making. Against the backdrop of ICI being one of the standard treatments for advanced non-small cell lung cancer, antibody level detection can serve as a valuable tool for clinical decision-making. Specifically, the expression levels of three AAbs, particularly the MAX AAb, can aid in predicting treatment response before initiation and dynamically monitoring the AAb panel post-treatment, facilitating timely detection of disease progression. The identified efficacy biomarkers in this study are intended solely for assisting in the monitoring of treatment efficacy in clinical settings, and the specific treatment approach should be selected based on the patient's clinical history, additional diagnostic tests, and overall treatment plan. In future research, we plan to conduct larger-scale, multicenter validation studies to establish robust guidelines for the clinical application of our detection method.

This study has several limitations. Firstly, it is a single-center study and lacks external center verification. Secondly, the number of dynamic paired samples included in the study is small, and the cohort consists of aNSCLC patients with different histological types, which could introduce heterogeneity. Furthermore, there was an absence of indicator correction, such as PD-L1 or TMB, and a comprehensive exploration of the molecular functions of these biomarkers was not conducted.

## Conclusions

In summary, our study successfully identified and validated a panel of two AAbs for pre-treatment assessment and a panel of two AAbs for on-treatment monitoring, demonstrating significant prognostic value across three cohorts receiving chemoimmunotherapy. These biomarkers exhibited promising predictive potential in distinguishing between responders and non-responders and effectively discriminating PFS outcomes. Notably, MAX and TAPBP AAbs displayed distinct kinetics between responders and non-responders, with MAX showing prognostic value at various levels, including mRNA, protein, and AAb. These predictive biomarkers hold promise for enhancing the precision of chemoimmunotherapy applications and further understanding of the molecular mechanisms associated with these biomarkers will advance our comprehension of the efficacy heterogeneity observed in chemoimmunotherapy.

## Ethics approval and consent to participate

This study has been approved by the Ethics Committee of the National Cancer Center/National Clinical Research Center for Cancer/Cancer Hospital, Chinese Academy of Medical Sciences & Peking Union Medical College (No. 19-019/1804). All experiments were executed according to the Declaration of Helsinki.

## Consent for publication

All authors agreed to submit for consideration for publication in this journal.

## Data availability

The datasets used and/or analyzed during the current study are available from the corresponding author on reasonable request.

## Supplemental data

This article contains supplemental data (20, 21, 22, 23, 24, 25, 30, 53, 54, 55, 56, 57).

## Conflict of interest

The authors declare no competing interests.
