# Supplementary material for: High-Throughput Antigen Microarray Identifies Longitudinal Prognostic Autoantibody for Chemoimmunotherapy in Advanced Non-Small Cell Lung Cancer
Source: Mol Cell Proteomics. 2024 Mar 20;23(5):100749. doi: 10.1016/j.mcpro.2024.100749 (PMC11070596; doi:10.1016/j.mcpro.2024.100749)
Supplement: Supplemental Figures [file mmc1.docx]

**Supplement Figures**

**
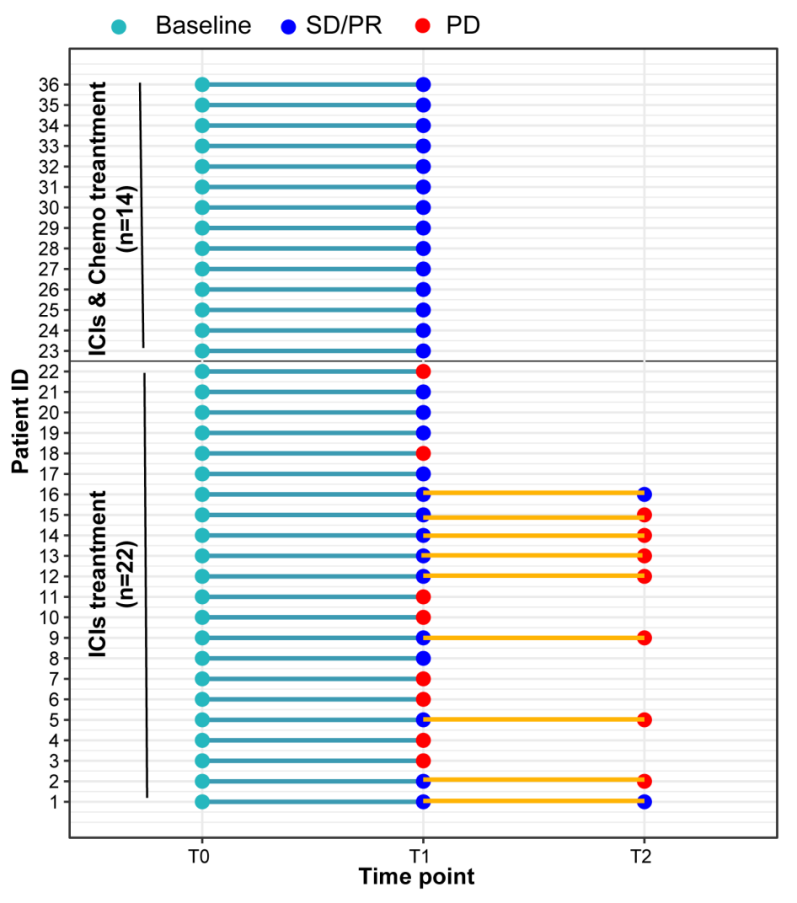
**

**Figure S1.** **81 sampling times of 36 aNSCLC patients in the discovery phase.**

*Abbreviation: aNSCLC: advanced non-small cell lung cancer; ICIs: immune checkpoint inhibitors; ICIs & Chemo: immune checkpoint inhibitors combined with chemotherapy; PR: partial remission; SD: steady disease; PD: progression disease; T0: baseline time point; T1: time point within three months treatment; T2: evaluation time point of progression.*


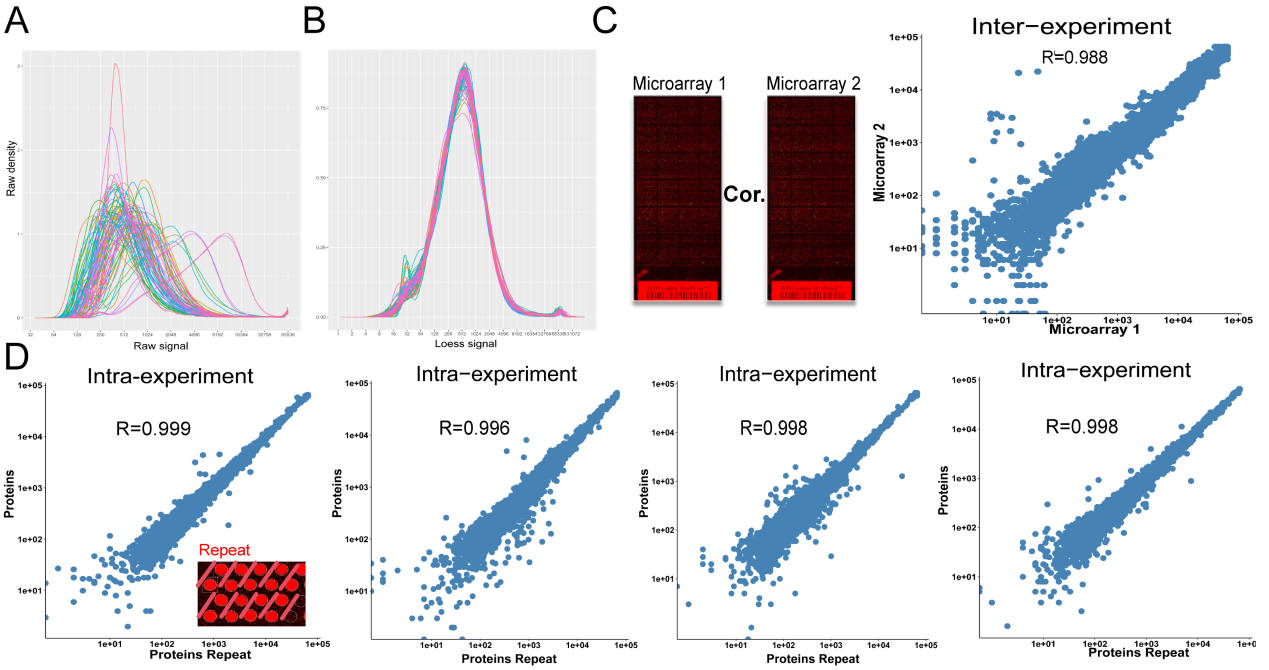


**Figure S2****. Normalization and reproducibility of autoantibody detection using high-density microarrays in the discovery phase. A-B.** Density distribution before and after loess normalization. **C.** Inter-experiment reproducibility between duplicates with one sample in two different microarrays. **D.** Intra-experiment reproducibility between protein duplicates within one microarray, encompassing both responder and non-responder pretreatment and post-treatment samples.

**
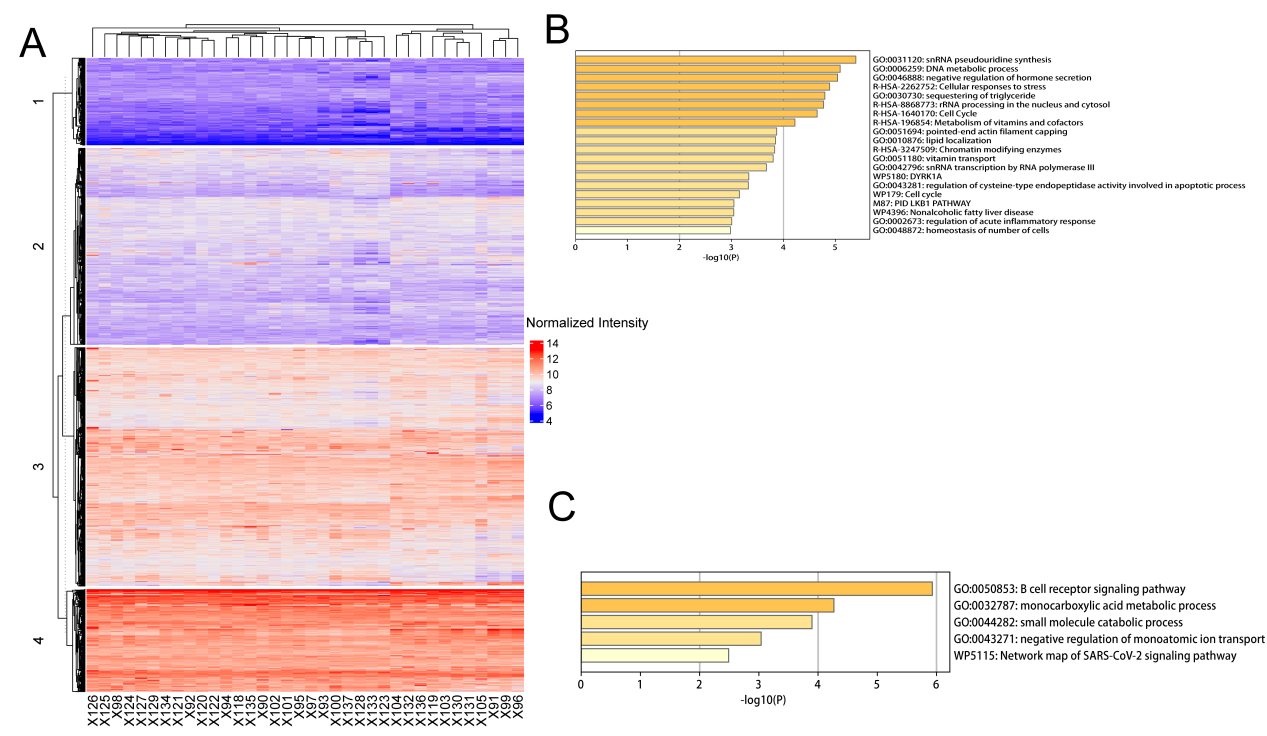
**

**Figure S3. Global AAbs profiling in aNSCLC (*n*=36) within the discovery phase. A.** Aggregation of ~21,000 AAbs into four clusters. **B-C.** Functional enrichment of AAbs targeted-proteins in Cluster 1 and Cluster 4, respectively.

*Abbreviation: AAbs: autoantibodies; aNSCLC: advanced non-small cell lung cancer.*


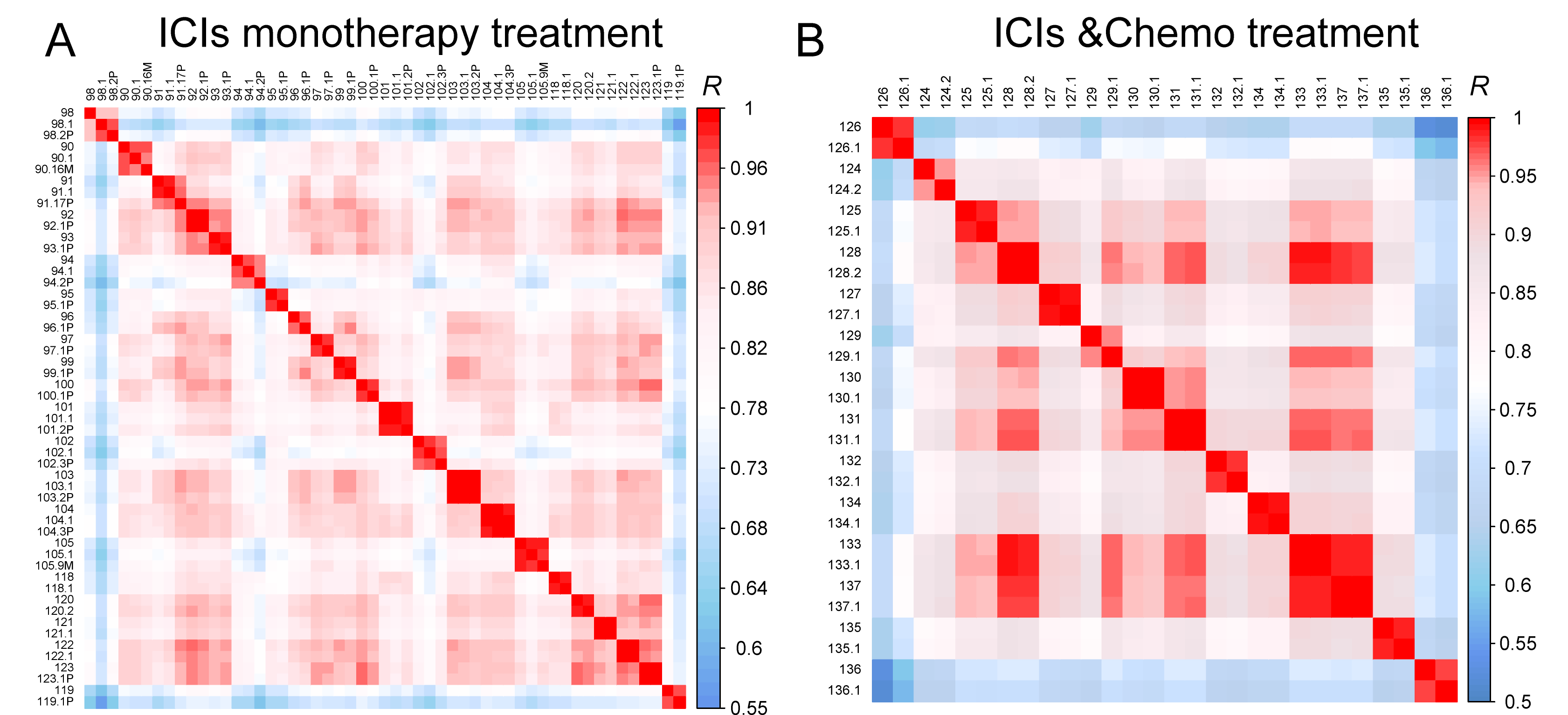


**Figure S4. Correlations of AAbs profiling in the same patients before and during ICIs treatment (*n*=36 with 72 samples) using high-density microarrays in the discovery phase.** **A-B.** Correlations of AAbs profiling are delineated in patients receiving ICIs monotherapy (*n*=22 with 44 samples) and ICIs combined with chemotherapy (*n*=14 with 28 samples), respectively.

*Abbreviation: AAbs: autoantibodies; ICIs: immune checkpoint inhibitors; ICIs & Chemo: immune checkpoint inhibitors combined with chemotherapy.*


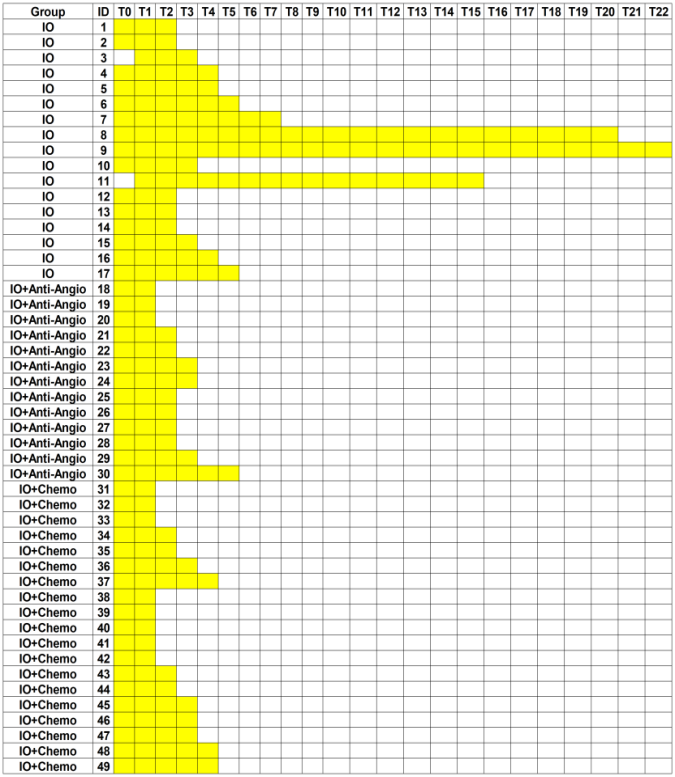


**Figure S5.** Dynamic sampling times among 49 aNSCLC patients in the verification phase, categorized into subgroups: 17 patients receiving ICIs monotherapy, 13 patients receiving ICIs combined with angiogenesis inhibitors, and 19 patients receiving ICIs combined with chemotherapy.

*Abbreviation: aNSCLC: advanced non-small cell lung cancer; ICIs: immune checkpoint inhibitors.*


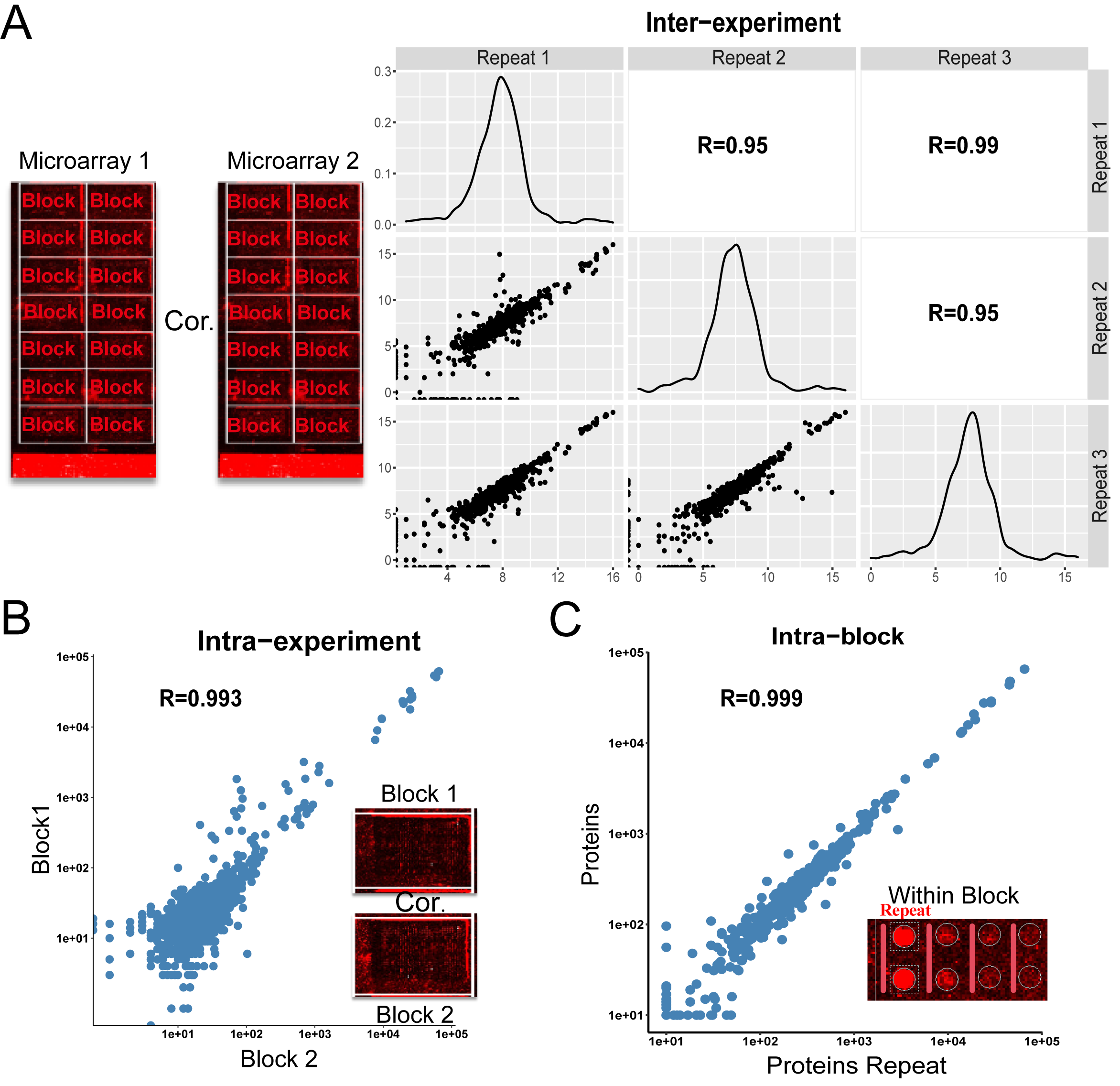


**Figure S6. Reproducibility of AAbs detection using aNSCLC-focused microarrays in the verification phase. A.** Inter-experiment reproducibility between duplicates with one sample in three different microarrays. **B.** Intra-experiment reproducibility between duplicates with one sample in different blocks within one microarray. **C.** Intra-block reproducibility between protein duplicates within one block.

*Abbreviation: AAbs: autoantibodies; aNSCLC: advanced non-small cell lung cancer.*


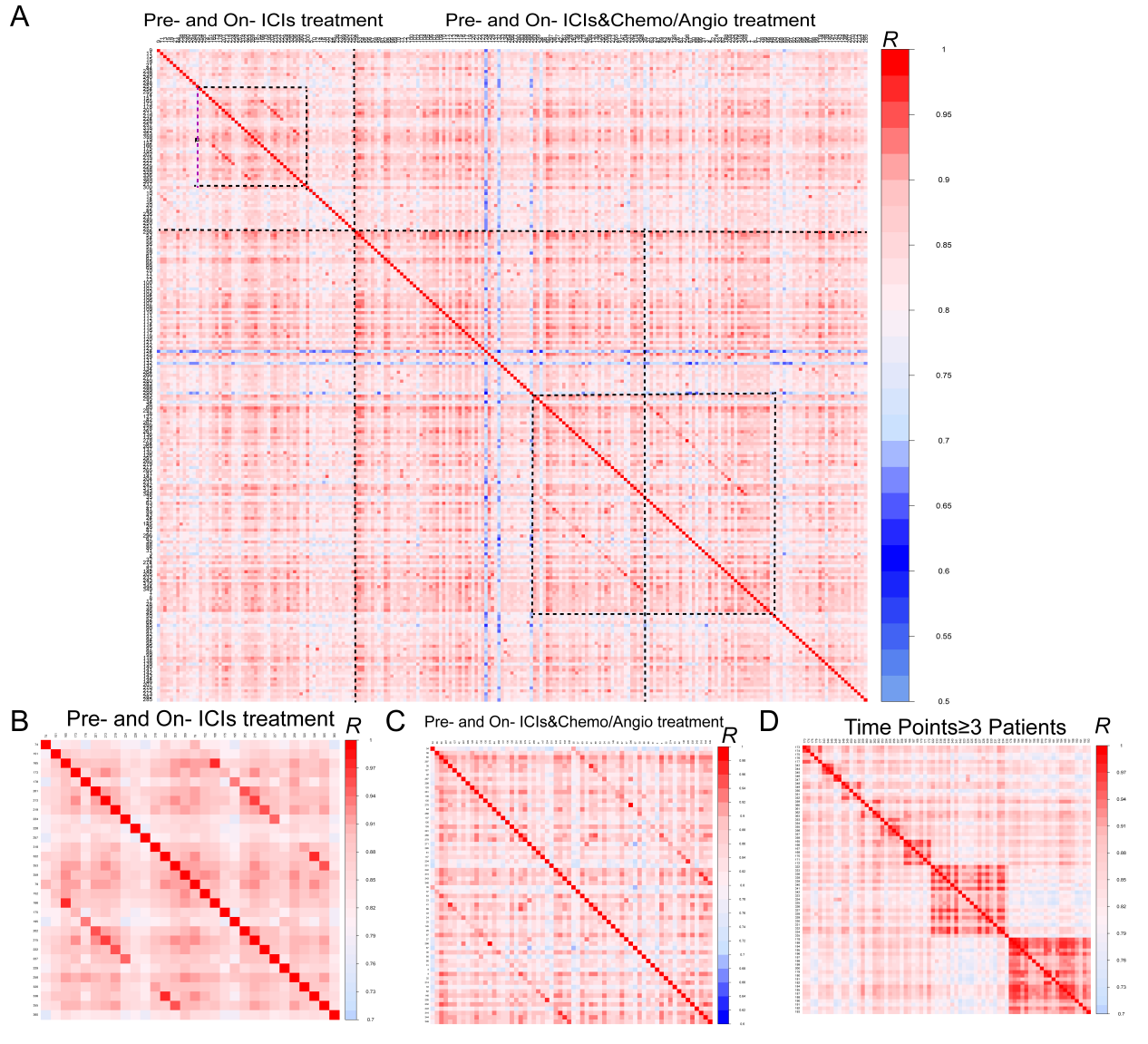


**Figure S7. Correlations of AAbs profiling in the same patients during before and during treatment (*n*=47) using aNSCLC-focused microarrays in the verification phase. A-C.** Heatmap depicting AAbs profilings correlations among patients treated with ICIs monotherapy (*n*=15 with 30 samples), ICIs combined with chemotherapy (*n*=19 with 38 samples), and ICIs combined with angiogenesis inhibitors (*n*=13 with 26 samples). **D.** Exploring AAbs profilings correlations in patients with more than three time points samples (*n*=23).

*Abbreviation: AAbs: autoantibodies; aNSCLC: advanced non-small cell lung cancer; ICIs: immune checkpoint inhibitors.*


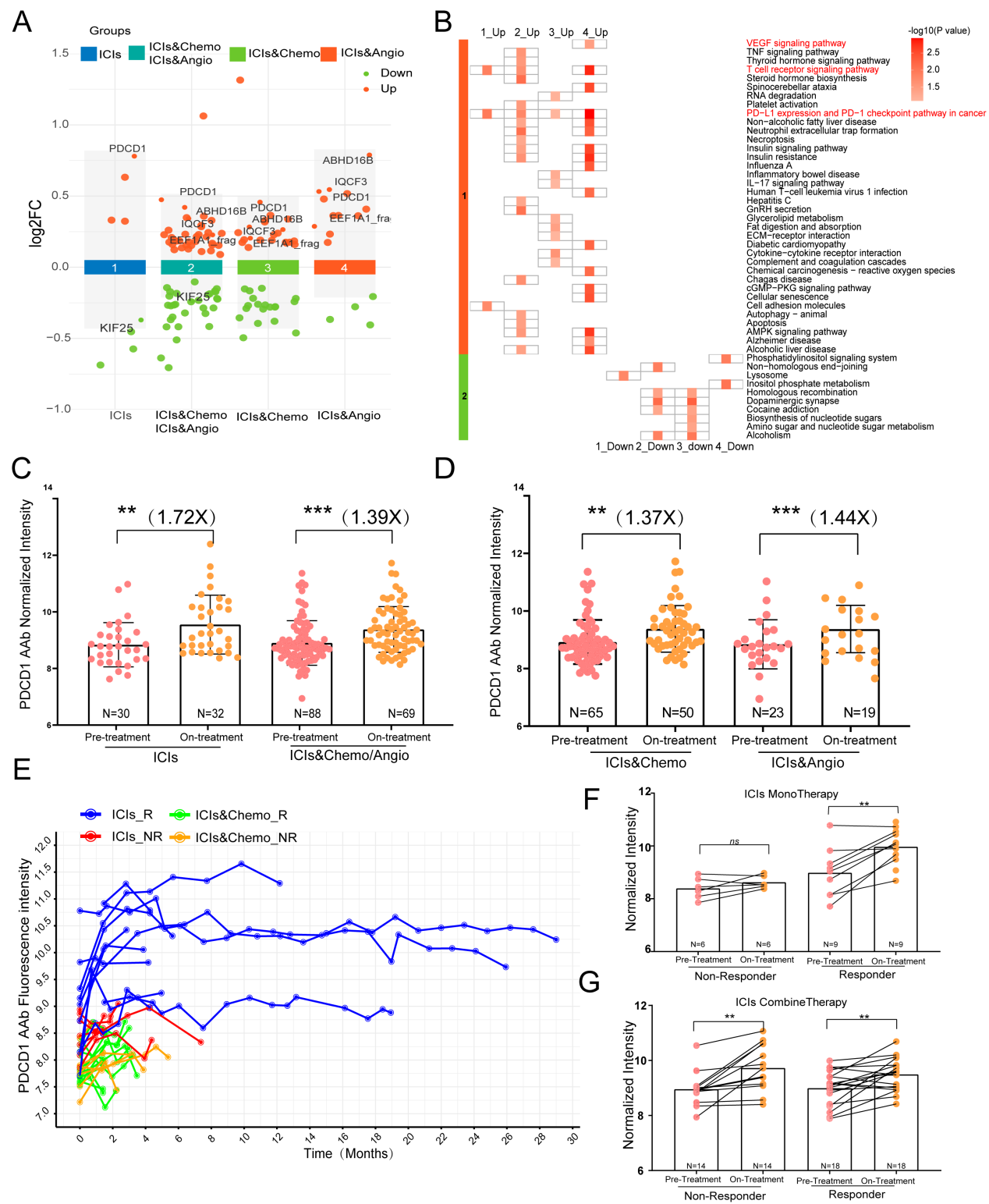


**Figure S8. Differential analyses of AAbs between pre- and on-ICIs monotherapy (*n*=30, 32), chemoimmunotherapy (*n*=65, 50), and ICIs combined with angiogenesis inhibitor (*n*=23, 19) using aNSCLC-focused microarrays in the verification phase.** **A.** Multi-volcano map displaying differential AAbs between pre- and on-treatment groups across ICIs monotherapy, chemoimmunotherapy and ICIs combined with angiogenesis inhibitor therapy. **B.** Biological pathways related to differential AAbs between pre- and on-treatment groups. **C-D.** Changes in anti-PDCD1 AAb pre- and on-ICIs monotherapy, chemoimmunotherapy and ICIs combined with angiogenesis inhibitor therapy. **E.** Heatmap of dynamic changes in anti-PDCD1 AAb in same patients pre- and on-ICIs monotherapy or chemoimmunotherapy (*n*=49). **F-G.** Comparison of PDCD1 AAb changes between responder and non-responder groups among paired patients receiving ICIs monotherapy (*n*=15) and chemoimmunotherapy (*n*=32).

*Abbreviation: AAbs: autoantibodies; ICIs: immune checkpoint inhibitors; aNSCLC: advanced non-small cell lung cancer; PDCD1: programmed cell death protein 1. * p < 0.05, ** p < 0.01, *** p < 0.001, **** p < 0.0001, ns: not significant.*


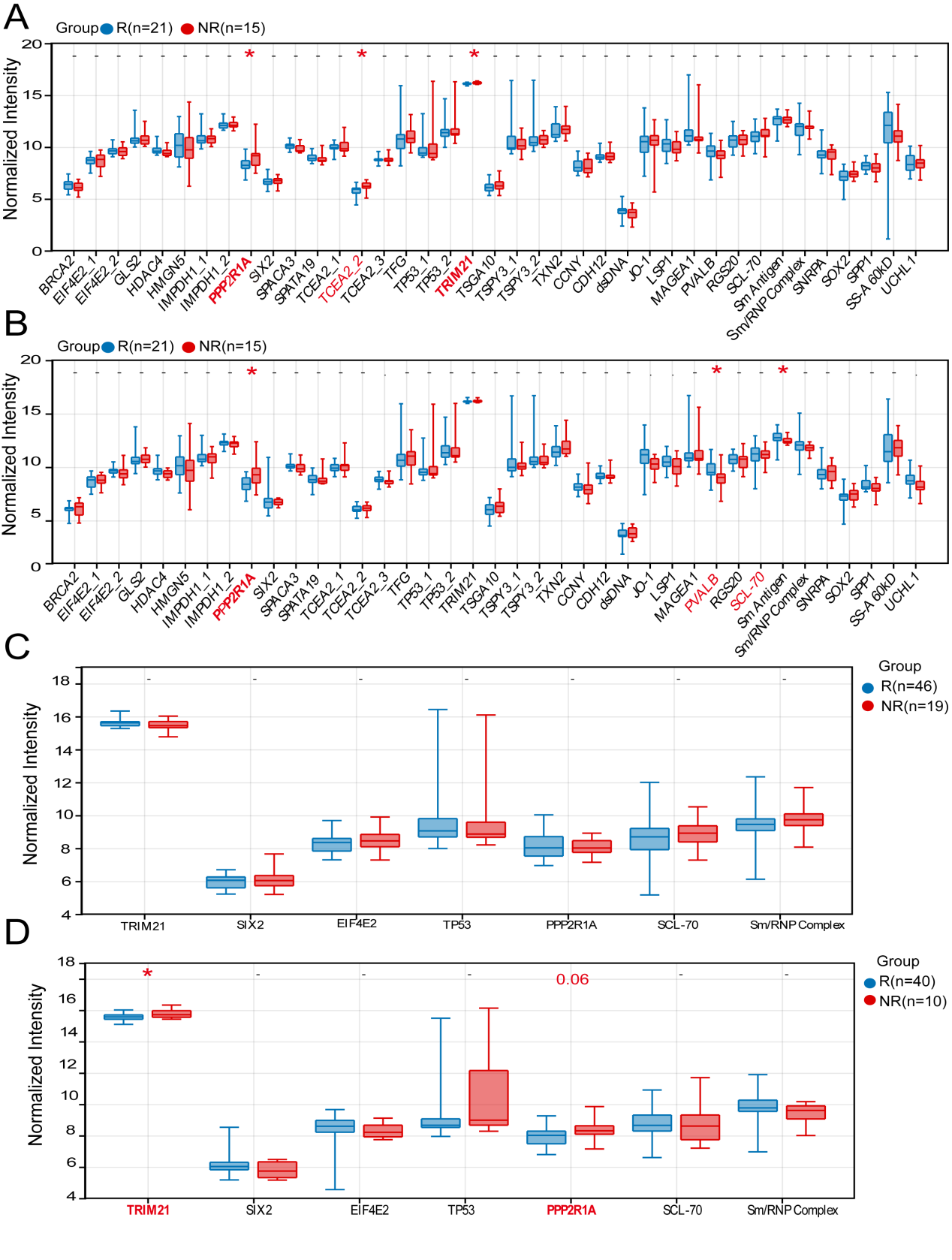


**Figure S9. Comparisons of AAbs reported in literature between R and NR groups before and during ICIs therapy in both the discovery and verification phases.** **A-B.** Comparisons of 36 AAbs between the R and NR groups before and after ICIs monotherapy and chemoimmunotherapy in the discovery phase (*n*=36). **C-D.** Comparisons of 7 AAbs between the R and NR groups before and after chemoimmunotherapy in the verification phase (*n*=65 pre-treatment and 50 on-treatment).

*Abbreviation: AAbs: autoantibodies; R: represented responder who achieving complete remission, partial remission, or stable disease within three months of treatment; NR: represented non-responder who achieving disease progression within three months of treatment; ICIs: immune checkpoint inhibitors. * p < 0.05, ** p < 0.01, *** p < 0.001, **** p < 0.0001, -: not significant.*


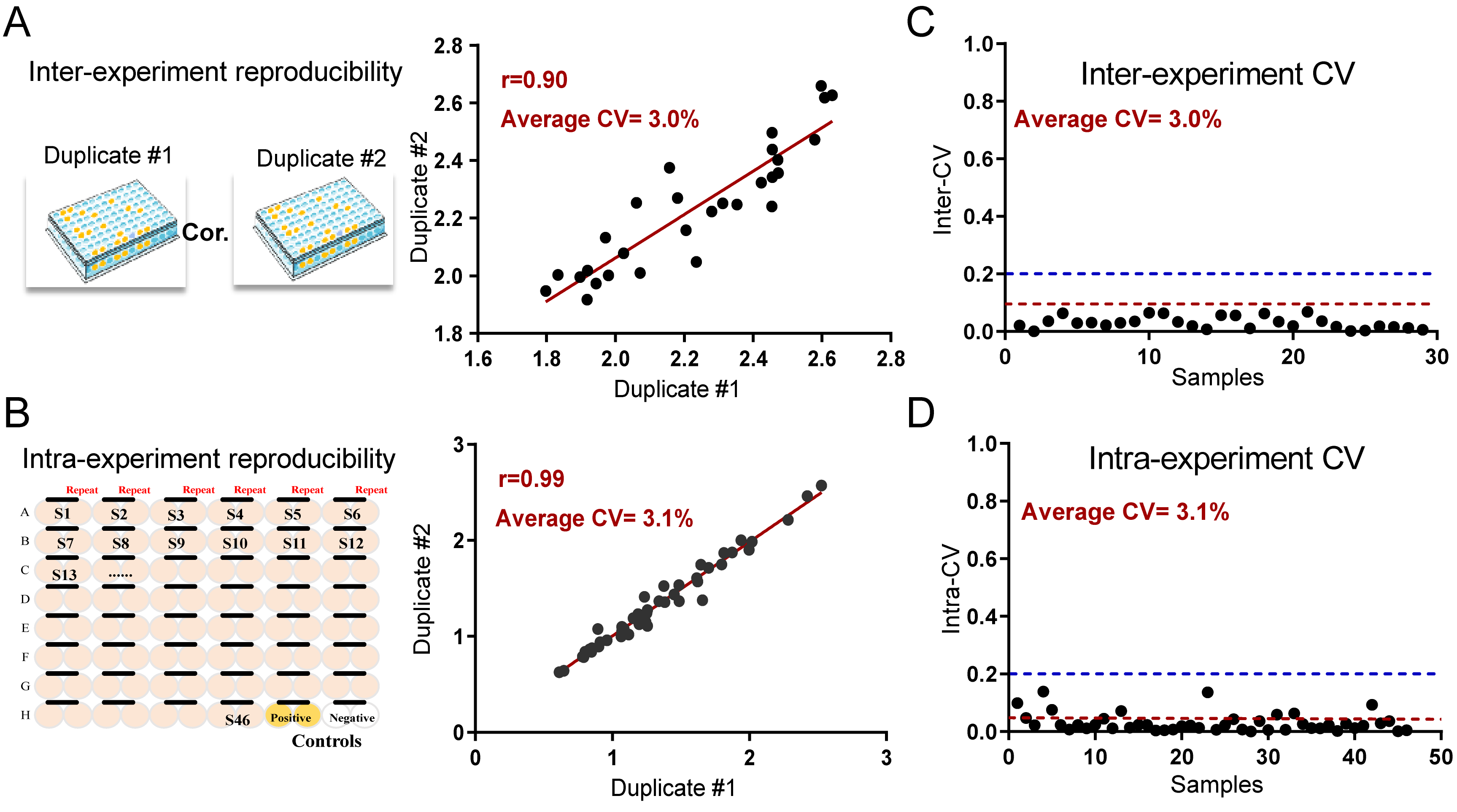


**Figure S10. Reproducibility and coefficient of variation (CV) in the detection of AAbs using ELISA tests in the validation phase.** **A.** Inter-experiment reproducibility between duplicates in different plates with 29 samples in two different plates. **B.** Intra-experiment reproducibility between duplicate wells in different proteins within one plate. **C-D.** Inter- and intra-experiment CV (CV = standard deviation of duplicate wells (σ) / mean of duplicate wells (μ)).

*Abbreviation: AAbs: autoantibodies; aNSCLC: advanced non-small cell lung cancer.*


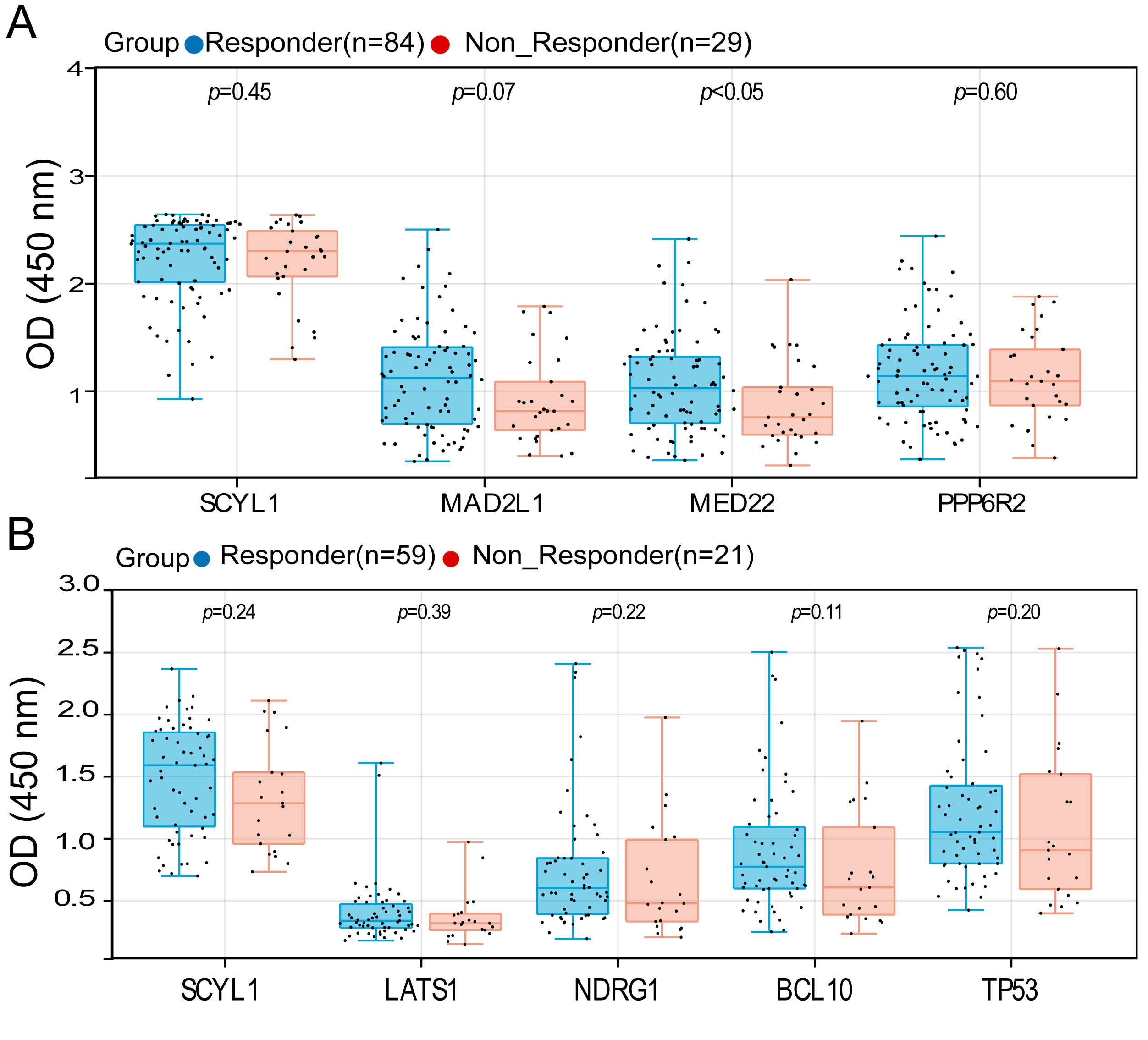


**Figure S11. Validation of the eight AAbs in discriminating responder and non-responder patients in the validation phase. A.** Comparison of the responder and non-responder groups according to SCYL1, MAD2L1, MED22, and PPP6R2 AAbs before chemoimmunotherapy. **B.** Comparison of the responder and non-responder groups according to SCYL1, LATS1, NDRG1, BCL10 and TP53 AAbs after chemoimmunotherapy.

*Note: The MED22 AAb level shows significant differences between responders and non-responders (p < 0.05); however, this contradicts the verification phase results, where MED22 was higher in non-responders than responders.*

*Abbreviation: OD: optical density; Responder: patients achieving complete remission, partial remission, or stable disease within three months of treatment; Non-Responder: patients achieving disease progression within three months of treatment.*

**
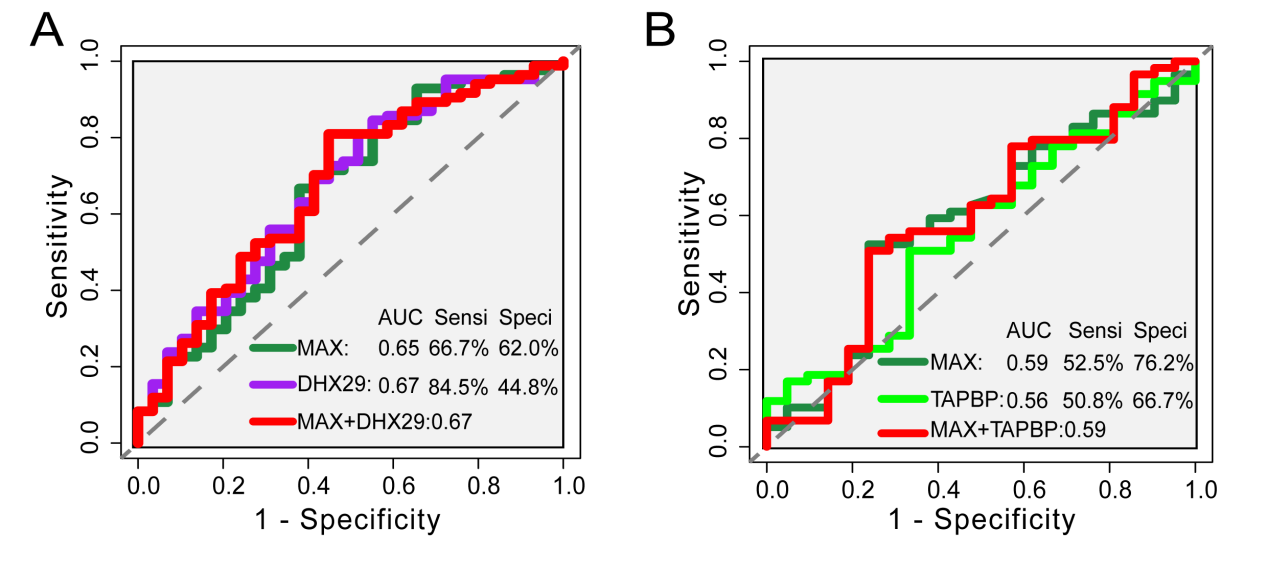
**

**Figure S12. ROC curves of the three AAbs for predicting R and NR patients before and during chemoimmunotherapy in the validation phase. A.** ROC curves of the MAX and DHX29 AAbs for predicting R and NR patients before chemoimmunotherapy (*n*=113). **B.** ROC curves of the MAX and TAPBP AAbs for predicting R and NR patients during chemoimmunotherapy (*n*=80).

*Abbreviation: ROC: receiver operating characteristic; AAbs: autoantibodies; R: represented responder who achieving complete remission, partial remission, or stable disease within three months of treatment; NR: represented non-responder who achieving disease progression within three months of treatment.*


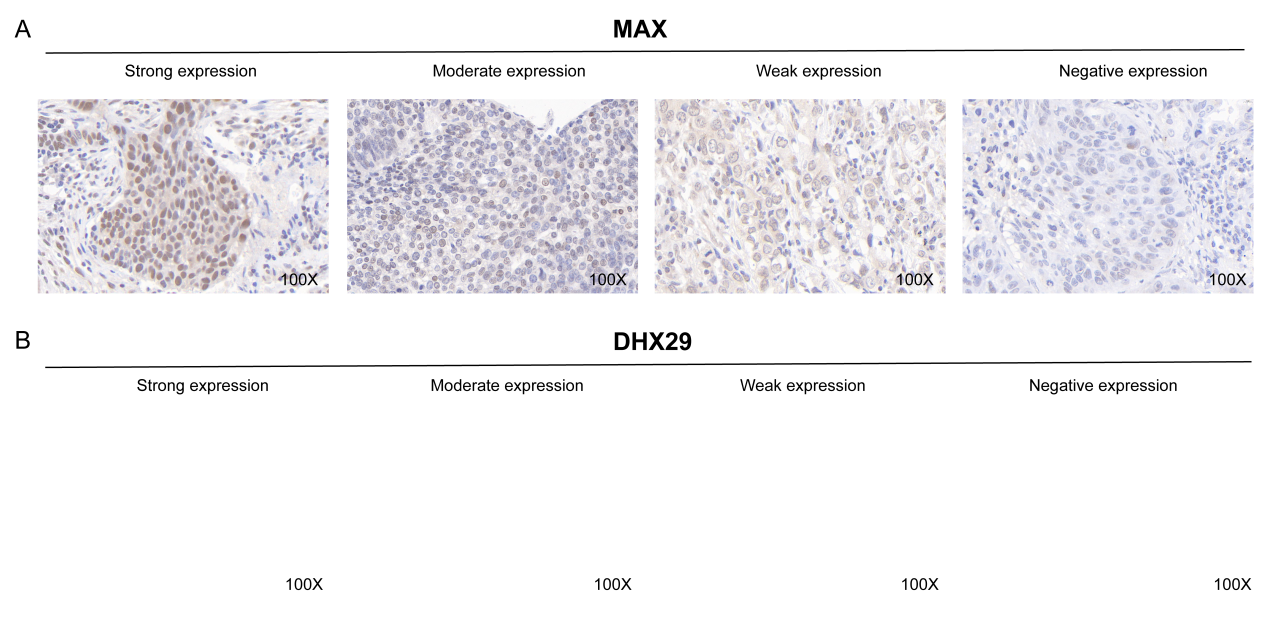


**Figure S13. Representative** **immunohistochemistry stain intensity of MAX expression (100X) (*n*=30).** MAX expression levels from strong, moderate, weak to negative.


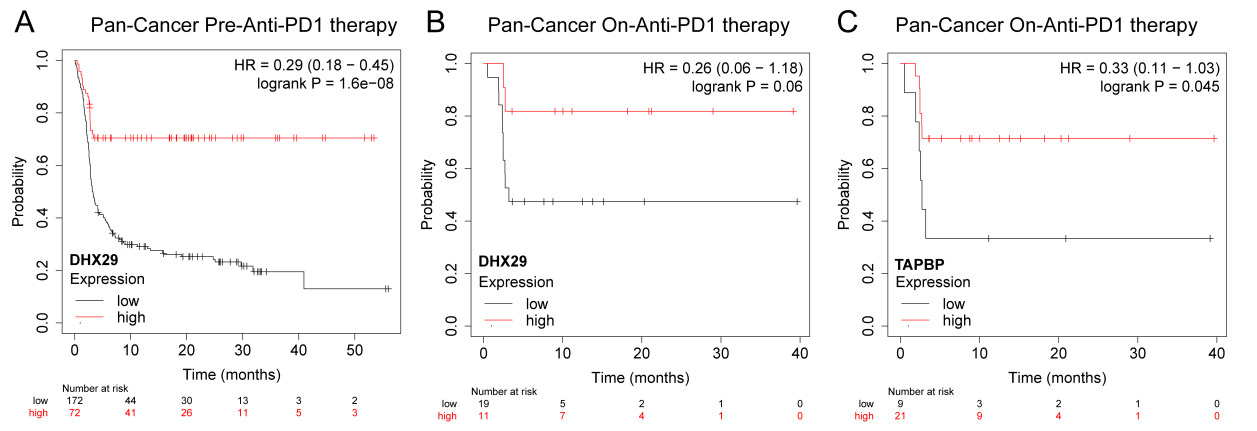


**Figure S14. Predictive value of DHX29 and TAPBP mRNAs in determining PFS before and during anti-PD1 therapy in K-M plot database.** **A-B.** DHX29 mRNA predictive potential for PFS before and during anti-PD1 therapy. **C.** TAPBP mRNA predictive capacity for PFS during anti-PD1 therapy.

*Abbreviation: mRNA: messenger RNA; PFS: progression-free survival; PD-1: programmed death-1.*
