# Supplementary material for: High-Throughput Antigen Microarray Identifies Longitudinal Prognostic Autoantibody for Chemoimmunotherapy in Advanced Non-Small Cell Lung Cancer
Source: Mol Cell Proteomics. 2024 Mar 20;23(5):100749. doi: 10.1016/j.mcpro.2024.100749 (PMC11070596; doi:10.1016/j.mcpro.2024.100749)
Supplement: Supplemental Tables [file mmc2.docx]

**Supplement Tables**

**Table S1. Post-treatment (T1) clinical characteristics of study cohorts (*n*=171).**

| **Characteristics** | **Cohorts** | **Discovery**  **(*n*=36)** | | **Verification**  **(*n*=87)** | | | **Validation**  **(*n*=80)** |
| --- | --- | --- | --- | --- | --- | --- | --- |
|  |  | **ICIs** | **ICIs &Chemo** | **ICIs** | **ICIs &**  **Chemo** | **ICIs &Angio** | **ICIs &**  **Chemo** |
|  | **n** | **22** | **14** | **25** | **50** | **12** | **80** |
| **Age (year)** | **Median** | 60 | 63.5 | 64 | 65 | 61 | 65 |
|  | **Range** | 32-74 | 33-71 | 43-74 | 33-79 | 48-84 | 36-80 |
| **Gender** | **Male** | 18 | 12 | 22 | 41 | 9 | 68 |
|  | **Female** | 4 | 2 | 3 | 9 | 3 | 12 |
| **Smoking** | **No** | 7 | 3 | 7 | 11 | 4 | 22 |
|  | **Yes** | 15 | 11 | 18 | 39 | 8 | 58 |
| **ECOG** | **0** | 8 | 6 | 9 | 24 | 6 | 22 |
|  | **1** | 14 | 8 | 12 | 6 | 5 | 27 |
|  | **2** | 0 | 0 | 2 | 3 | 1 | 28 |
|  | **Unknown** | 0 | 0 | 2 | 17 | 0 | 3 |
| **Histology** | **ADC** | 10 | 5 | 9 | 31 | 11 | 32 |
|  | **SCC** | 11 | 8 | 16 | 17 | 1 | 45 |
|  | **ASC** | 1 | 0 | 0 | 0 | 0 | 2 |
|  | **LCLC** | 0 | 1 | 0 | 2 | 0 | 1 |
| **Stage** | **Ⅲ** | 7 | 3 | 6 | 20 | 3 | 40 |
|  | **Ⅳ** | 15 | 11 | 19 | 30 | 9 | 40 |
| **Clinical Benefit** | **R** | 12 | 9 | 16 | 40 | 3 | 59 |
|  | **NR** | 10 | 5 | 9 | 10 | 9 | 21 |
| **ICIs Line** | **1** | 0 | 3 | 5 | 31 | 4 | 77 |
|  | **2** | 7 | 5 | 9 | 11 | 4 | 2 |
|  | **≥3** | 15 | 6 | 11 | 8 | 4 | 1 |
| ***EGFR*** | **Mutation** | 2 | 3 | 2 | 8 | 3 | 6 |
|  | **Wild** | 13 | 6 | 10 | 22 | 8 | 30 |
|  | **Unknown** | 7 | 5 | 13 | 20 | 1 | 44 |

*Note: In the validation phase, 52 cases had overlapping pre-treatment blood samples with those from the verification phase, and 32 cases had overlapping post-treatment blood samples with those from the verification phase.*

*Abbreviation: ICIs: immune checkpoint inhibitors; ICIs & Chemo: immune checkpoint inhibitors combined with chemotherapy; ICIs & Angio: immune checkpoint inhibitors combined with angiogenic inhibitors; ECOG: Eastern Cooperative Oncology Group; ADC: adenocarcinoma; SCC: squamous carcinoma; ASC: adenosquamous carcinoma; LCC: large cell carcinoma; R: represented responder who achieving complete remission, partial remission, or stable disease within three months of treatment; NR: represented non-responder who achieving disease progression within three months of treatment; EGFR: epidermal growth factor receptor.*

**Table S2. Clinical characteristics of the IHC cohort (*n*=30).**

| **Characteristics** | **Cohorts** | **Validation** |
| --- | --- | --- |
|  |  | **IHC cohort** |
|  | ***n*** | **30** |
| **Age (year)** | **Median** | 62 |
|  | **Range** | 38-74 |
| **Gender** | **Male** | 25 |
|  | **Female** | 5 |
| **Smoking** | **No** | 10 |
|  | **Yes** | 20 |
| **ECOG** | **0** | 23 |
|  | **1** | 7 |
| **Histology** | **ADC** | 18 |
|  | **SCC** | 12 |
|  | **ASC** | 0 |
| **Stage** | **I** | 3 |
|  | **II** | 5 |
|  | **Ⅲ** | 12 |
|  | **Ⅳ** | 10 |
| **Clinical Benefit** | **Responder** | 20 |
|  | **Non-responder** | 10 |
| **ICIs Line** | **1** | 12 |
|  | **2** | 11 |
|  | **≥3** | 7 |
| *EGFR* | Mutation | 9 |
|  | **Wild type** | 15 |
|  | **Unknown** | 6 |

*Abbreviation: IHC: immunohistochemistry; ECOG: Eastern Cooperative Oncology Group; ADC: adenocarcinoma; SCC: squamous carcinoma; ASC: adenosquamous carcinoma; LCC: large cell carcinoma; Responder: patients achieving complete remission, partial remission, or stable disease within three months of treatment; Non-Responder: patients achieving disease progression within three months of treatment; ICIs: immune checkpoint inhibitors; EGFR: epidermal growth factor receptor.*

**Table S3. Differential AAbs (*n*=507) before and after ICIs treatment using the aNSCLC-focused microarray in the verification cohort.**

| **ID** | **Name** | **Group** | ***FC*** | ***p.Value*** |
| --- | --- | --- | --- | --- |
| JHU29139.B20C19 | VEGF | Chemoimmunotherapy | 0.12 | *0.02* |
| JHU17014.B15C31 | ZNF517 | Chemoimmunotherapy | 0.32 | *0.01* |
| JHU30297.B20C28 | PRPF40B | Chemoimmunotherapy | 0.34 | *0.02* |
| JHU15986.B15C22 | GLP2R | Chemoimmunotherapy | 0.35 | *0.03* |
| JHU18909.B16C17 | HAPLN2 | Chemoimmunotherapy | 0.36 | *<0.01* |
| JHU01226.B14C22 | SNAI2 | Chemoimmunotherapy | 0.38 | *0.05* |
| JHU19946.B15C3 | U2SURP | Chemoimmunotherapy | 0.39 | *0.01* |
| JHU29270.B18C22 | ZNF814 | Chemoimmunotherapy | 0.39 | *0.02* |
| JHU08871.B7C15 | IGL@ | Chemoimmunotherapy | 0.40 | *0.01* |
| JHU19500.B14C29 | NEK5 | Chemoimmunotherapy | 0.40 | *0.02* |
| JHU19873.B16C31 | ZNF219 | Chemoimmunotherapy | 0.40 | *0.02* |
| JHU29789.B17C24 | DEDD2 | Chemoimmunotherapy | 0.41 | *0.01* |
| JHU18940.B13C22 | OLFML1 | Chemoimmunotherapy | 0.41 | *0.02* |
| JHU16477.B11C20 | TRAC | Chemoimmunotherapy | 0.41 | *0.01* |
| JHU24164.B16C28 | DHX30 | Chemoimmunotherapy | 0.41 | *0.03* |
| JHU16939.B14C21 | CHAT | Chemoimmunotherapy | 0.42 | *0.04* |
| JHU19541.B14C14 | TLK2 | Chemoimmunotherapy | 0.42 | *0.03* |
| JHU19357.B15C22 | AMH | Chemoimmunotherapy | 0.42 | *0.01* |
| JHU26022.B19C3 | MBNL3 | Chemoimmunotherapy | 0.42 | *<0.01* |
| JHU16141.B15C32 | USP21 | Chemoimmunotherapy | 0.42 | *0.01* |
| JHU13637.B9C6 | KLHDC8A | Chemoimmunotherapy | 0.43 | *0.03* |
| JHU01470.B16C4 | DXO | Chemoimmunotherapy | 0.44 | *0.02* |
| JHU30243.B19C28 | TRIM50 | Chemoimmunotherapy | 0.45 | *0.01* |
| JHU24727.B20C24 | MTMR10 | Chemoimmunotherapy | 0.45 | *<0.01* |
| JHU25505.B19C16 | POU3F3 | Chemoimmunotherapy | 0.46 | *0.01* |
| JHU29594.B19C22 | RDM1 | Chemoimmunotherapy | 0.47 | *0.01* |
| JHU30194.B20C11 | ACP7 | Chemoimmunotherapy | 0.47 | *0.02* |
| JHU00511.B17C10 | DNTT | Chemoimmunotherapy | 0.47 | *<0.01* |
| JHU30351.B19C20 | PDE10A | Chemoimmunotherapy | 0.47 | *0.01* |
| JHU09562.B5C31 | NKRF | Chemoimmunotherapy | 0.47 | *0.01* |
| JHU13221.B9C6 | C20orf24 | Chemoimmunotherapy | 0.48 | *0.02* |
| JHU18604.B14C12 | TXNDC2 | Chemoimmunotherapy | 0.48 | *0.05* |
| JHU18500.B14C30 | SF3A2 | Chemoimmunotherapy | 0.48 | *0.01* |
| Auto-antigen.B20C4R42 | Sm/RNP Complex | Chemoimmunotherapy | 0.48 | *0.04* |
| Auto-antigen.B20C6R41 | SCL-70 | Chemoimmunotherapy | 0.49 | *0.04* |
| JHU18997.B16C15 | CYP11B2 | Chemoimmunotherapy | 0.49 | *0.01* |
| JHU30089.B18C32 | MOGAT2 | Chemoimmunotherapy | 0.50 | *0.01* |
| JHU16418.B12C23 | HMGA1_frag | Chemoimmunotherapy | 0.51 | *0.04* |
| JHU05311.B3C31 | G3BP1 | Chemoimmunotherapy | 0.51 | *0.02* |
| JHU01792.B15C32 | PABPC3 | Chemoimmunotherapy | 0.51 | *0.02* |
| JHU15257.B12C21 | PRDM15 | Chemoimmunotherapy | 0.51 | *0.03* |
| JHU15305.B11C12 | DDX1 | Chemoimmunotherapy | 0.52 | *0.02* |
| JHU15381.B11C15 | ZNF571 | Chemoimmunotherapy | 0.52 | *0.01* |
| JHU07605.B6C26 | DSE | Chemoimmunotherapy | 0.52 | *0.02* |
| JHU29437.B18C30 | ZMIZ2 | Chemoimmunotherapy | 0.52 | *0.03* |
| JHU20818.B19C7 | AGBL5 | Chemoimmunotherapy | 0.52 | *0.04* |
| JHU18348.B16C24 | CILP | Chemoimmunotherapy | 0.52 | *0.01* |
| JHU14294.B11C8 | DHX29 | Chemoimmunotherapy | 0.52 | *0.04* |
| JHU13222.B12C19 | CHTF8 | Chemoimmunotherapy | 0.53 | *0.01* |
| JHU29940.B18C10 | IQCF3 | Chemoimmunotherapy | 0.53 | *0.01* |
| JHU18046.B15C16 | ZNF197 | Chemoimmunotherapy | 0.53 | *0.04* |
| JHU18872.B15C15 | TCTN3 | Chemoimmunotherapy | 0.53 | *0.02* |
| JHU24603.B19C22 | CCDC158 | Chemoimmunotherapy | 0.54 | *0.03* |
| JHU15733.B12C2 | OR5D16 | Chemoimmunotherapy | 0.54 | *0.01* |
| JHU16467.B10C5 | SCYL1 | Chemoimmunotherapy | 0.54 | *0.03* |
| JHU10476.B5C27 | ZC2HC1A | Chemoimmunotherapy | 0.54 | *0.02* |
| JHU18611.B15C32 | ADNP2 | Chemoimmunotherapy | 0.54 | *0.01* |
| JHU15857.B10C32 | ZIK1 | Chemoimmunotherapy | 0.54 | *<0.01* |
| JHU19734.B15C19 | Glis2 | Chemoimmunotherapy | 0.54 | *0.01* |
| JHU16707.B15C8 | ZNF546 | Chemoimmunotherapy | 0.55 | *0.02* |
| JHU08067.B15C18 | XIAP | Chemoimmunotherapy | 0.55 | *0.02* |
| JHU08968.B6C27 | LIMS1 | Chemoimmunotherapy | 0.55 | *0.01* |
| JHU15383.B10C27 | AGBL2 | Chemoimmunotherapy | 0.55 | *0.02* |
| JHU16807.B14C16 | ZNF768 | Chemoimmunotherapy | 0.55 | *0.03* |
| JHU11433.B15C5 | MAATS1 | Chemoimmunotherapy | 0.55 | *0.02* |
| JHU14248.B11C5 | SPATA5L1_frag | Chemoimmunotherapy | 0.55 | *0.03* |
| JHU18223.B15C26 | SMC6 | Chemoimmunotherapy | 0.55 | *0.01* |
| JHU25702.B19C1 | N4BP2L1 | Chemoimmunotherapy | 0.56 | *0.01* |
| JHU12690.B11C3 | PLEKHH3 | Chemoimmunotherapy | 0.56 | *0.01* |
| JHU14498.B14C23 | SRP19 | Chemoimmunotherapy | 0.56 | *0.02* |
| JHU12717.B11C23 | LMF2 | Chemoimmunotherapy | 0.56 | *0.05* |
| JHU17217.B16C19 | CENPBD1 | Chemoimmunotherapy | 0.56 | *0.04* |
| JHU05662.B11C27 | ZFP82 | Chemoimmunotherapy | 0.56 | *0.01* |
| JHU26774.B18C23 | DNAJC11 | Chemoimmunotherapy | 0.56 | *0.05* |
| JHU15580.B12C28 | AP1G1 | Chemoimmunotherapy | 0.57 | *0.02* |
| JHU19964.B14C2 | ZNF132 | Chemoimmunotherapy | 0.57 | *0.01* |
| JHU15984.B15C31 | MPV17L2 | Chemoimmunotherapy | 0.57 | *<0.01* |
| JHU19962.B16C5 | Q4vad4 | Chemoimmunotherapy | 0.57 | *0.03* |
| JHU02324.B14C29 | CFLAR | Chemoimmunotherapy | 0.57 | *<0.01* |
| JHU03904.B15C12 | NDUFS7 | Chemoimmunotherapy | 0.57 | *0.01* |
| JHU19392.B13C5 | KDM7A | Chemoimmunotherapy | 0.57 | *0.04* |
| JHU15974.B11C13 | CYP11B1 | Chemoimmunotherapy | 0.58 | *<0.01* |
| JHU17237.B15C25 | PSTK | Chemoimmunotherapy | 0.58 | *0.03* |
| JHU16427.B11C23 | BC073758 | Chemoimmunotherapy | 0.58 | *0.02* |
| JHU16575.B10C14 | ZKSCAN8 | Chemoimmunotherapy | 0.58 | *0.01* |
| JHU28208.B19C11 | ZNF266 | Chemoimmunotherapy | 0.59 | *0.01* |
| JHU16437.B10C3 | IL17F | Chemoimmunotherapy | 0.59 | *0.01* |
| JHU02080.B14C15 | POLR2E | Chemoimmunotherapy | 0.59 | *0.05* |
| JHU16716.B14C22 | ZNF774 | Chemoimmunotherapy | 0.59 | *0.01* |
| JHU15978.B11C21 | EEF1A1_frag | Chemoimmunotherapy | 0.59 | *0.01* |
| JHU17088.B15C26 | RNASE9 | Chemoimmunotherapy | 0.59 | *<0.01* |
| JHU18674.B15C1 | EPX | Chemoimmunotherapy | 0.59 | *0.01* |
| JHU16763.B15C24 | PRDM13 | Chemoimmunotherapy | 0.60 | *<0.01* |
| JHU30031.B20C27 | FBXO16 | Chemoimmunotherapy | 0.60 | *0.03* |
| JHU15205.B12C21 | CHTF18 | Chemoimmunotherapy | 0.60 | *0.04* |
| JHU04387.B1C8 | SCCPDH | Chemoimmunotherapy | 0.60 | *0.04* |
| JHU25618.B19C25 | GALR3 | Chemoimmunotherapy | 0.60 | *0.01* |
| JHU18198.B16C2 | L3MBTL2 | Chemoimmunotherapy | 0.60 | *0.04* |
| JHU15309.B12C23 | EHHADH | Chemoimmunotherapy | 0.60 | *0.04* |
| JHU13688.B10C19 | ZNF766 | Chemoimmunotherapy | 0.60 | *0.02* |
| JHU14310.B11C22 | CCAR2 | Chemoimmunotherapy | 0.60 | *0.02* |
| JHU00125.B13C7 | DUSP26 | Chemoimmunotherapy | 0.61 | *0.02* |
| JHU19088.B14C17 | EMILIN3 | Chemoimmunotherapy | 0.61 | *0.01* |
| JHU05983.B8C4 | DDX28 | Chemoimmunotherapy | 0.61 | *0.02* |
| JHU18434.B15C30 | B3GAT1 | Chemoimmunotherapy | 0.61 | *0.02* |
| JHU14709.B11C27 | ZNF256 | Chemoimmunotherapy | 0.61 | *0.04* |
| JHU13078.B12C18 | KAT8 | Chemoimmunotherapy | 0.61 | *0.03* |
| JHU24725.B18C7 | ABHD16B | Chemoimmunotherapy | 0.61 | *0.01* |
| JHU17078.B16C2 | PIK3C2A | Chemoimmunotherapy | 0.61 | *0.02* |
| JHU17687.B15C25 | ZNF17 | Chemoimmunotherapy | 0.61 | *0.03* |
| JHU10493.B17C7 | AGO4 | Chemoimmunotherapy | 0.61 | *0.04* |
| JHU18832.B13C3 | CCL16 | Chemoimmunotherapy | 0.61 | *0.03* |
| JHU19879.B15C30 | ZNF366 | Chemoimmunotherapy | 0.61 | *0.04* |
| JHU14247.B11C3 | SNAI1 | Chemoimmunotherapy | 0.61 | *0.03* |
| JHU09360.B8C27 | LATS1 | Chemoimmunotherapy | 0.62 | *0.02* |
| JHU14296.B11C32 | AGO1 | Chemoimmunotherapy | 0.62 | *<0.01* |
| JHU18777.B16C18 | KCNH4 | Chemoimmunotherapy | 0.62 | *0.04* |
| JHU20194.B19C1 | PISD | Chemoimmunotherapy | 0.62 | *<0.01* |
| JHU19941.B16C15 | SNAI3 | Chemoimmunotherapy | 0.62 | *0.02* |
| JHU14708.B12C22 | ZNF140 | Chemoimmunotherapy | 0.62 | *0.02* |
| JHU14902.B9C18 | ZNF268 | Chemoimmunotherapy | 0.62 | *0.02* |
| JHU20693.B19C7 | RNF180 | Chemoimmunotherapy | 0.62 | *0.03* |
| JHU17083.B14C20 | PSKH2 | Chemoimmunotherapy | 0.63 | *0.04* |
| JHU03879.B4C26 | FGFR1OP2 | Chemoimmunotherapy | 1.62 | *0.01* |
| JHU11297.B5C14 | SEC24C | Chemoimmunotherapy | 1.62 | *0.01* |
| JHU07361.B5C7 | PLPP1 | Chemoimmunotherapy | 1.63 | *0.04* |
| JHU02063.B4C29 | MPHOSPH6 | Chemoimmunotherapy | 1.63 | *0.02* |
| JHU14837.B9C10 | PUDP | Chemoimmunotherapy | 1.64 | *0.04* |
| JHU15206.B12C10 | CLIC2 | Chemoimmunotherapy | 1.65 | *0.03* |
| JHU07695.B5C12 | CCNJL_frag | Chemoimmunotherapy | 1.65 | *0.05* |
| JHU02787.B1C17 | AKT1 | Chemoimmunotherapy | 1.65 | *0.01* |
| JHU03274.B4C16 | CASS4 | Chemoimmunotherapy | 1.68 | *0.03* |
| JHU04338.B2C8 | COMT | Chemoimmunotherapy | 1.68 | *0.01* |
| JHU00819.B6C9 | MED28 | Chemoimmunotherapy | 1.69 | *0.03* |
| JHU10724.B7C28 | SMPD3 | Chemoimmunotherapy | 1.69 | *0.02* |
| JHU14316.B9C14 | LPIN1 | Chemoimmunotherapy | 1.70 | *0.01* |
| JHU00964.B13C19 | AASDHPPT | Chemoimmunotherapy | 1.70 | *0.03* |
| JHU10682.B6C18 | RILPL1 | Chemoimmunotherapy | 1.70 | *0.01* |
| JHU16130.B10C2 | SH3D19 | Chemoimmunotherapy | 1.71 | *0.02* |
| JHU03948.B10C5 | ANXA6 | Chemoimmunotherapy | 1.72 | *0.05* |
| JHU05389.B4C2 | CD68 | Chemoimmunotherapy | 1.72 | *0.03* |
| JHU13014.B10C28 | ZBTB22 | Chemoimmunotherapy | 1.73 | *0.05* |
| JHU07059.B5C22 | MAP3K11 | Chemoimmunotherapy | 1.74 | *0.03* |
| JHU06355.B7C24 | CD53 | Chemoimmunotherapy | 1.75 | *0.02* |
| JHU10987.B8C4 | FAM231D | Chemoimmunotherapy | 1.75 | *0.02* |
| JHU01923.B4C20 | ASPH | Chemoimmunotherapy | 1.75 | *0.01* |
| JHU14372.B14C7 | RGCC | Chemoimmunotherapy | 1.75 | *0.04* |
| JHU19272.B13C10 | CRYL1 | Chemoimmunotherapy | 1.76 | *0.02* |
| JHU10989.B7C16 | LRFN1 | Chemoimmunotherapy | 1.76 | *0.05* |
| JHU12903.B9C4 | SPANXD | Chemoimmunotherapy | 1.77 | *0.02* |
| JHU16329.B9C1 | SRA1_frag | Chemoimmunotherapy | 1.77 | *0.01* |
| JHU25682.B18C6 | SYCE2 | Chemoimmunotherapy | 1.79 | *0.04* |
| JHU15932.B9C4 | RAP1GDS1 | Chemoimmunotherapy | 1.79 | *0.04* |
| JHU14405.B10C6 | PDPN | Chemoimmunotherapy | 1.79 | *0.02* |
| JHU17186.B13C1 | PML | Chemoimmunotherapy | 1.81 | *0.04* |
| JHU08641.B8C16 | ABTB1 | Chemoimmunotherapy | 1.81 | *0.01* |
| JHU12333.B9C26 | SYNJ2 | Chemoimmunotherapy | 1.82 | *0.05* |
| JHU00079.B13C7 | SKAP2 | Chemoimmunotherapy | 1.83 | *0.01* |
| JHU19803.B15C30 | DMTF1 | Chemoimmunotherapy | 1.84 | *<0.01* |
| JHU04903.B2C18 | ARL11 | Chemoimmunotherapy | 1.84 | *0.04* |
| JHU18030.B14C6 | SOCS7 | Chemoimmunotherapy | 1.85 | *0.01* |
| JHU07606.B6C5 | EIF4ENIF1 | Chemoimmunotherapy | 1.86 | *0.02* |
| JHU08382.B9C17 | FAM29A | Chemoimmunotherapy | 1.89 | *0.03* |
| JHU16515.B12C14 | NG_015859.1_frag | Chemoimmunotherapy | 1.90 | *0.03* |
| JHU12919.B10C25 | ZNF655_frag | Chemoimmunotherapy | 1.90 | *0.02* |
| JHU04122.B6C25 | CFAP43 | Chemoimmunotherapy | 1.91 | *0.02* |
| JHU11491.B5C21 | PPP6R2 | Chemoimmunotherapy | 1.91 | *0.03* |
| JHU06563.B5C2 | NAALADL2 | Chemoimmunotherapy | 1.91 | *0.03* |
| JHU13952.B9C27 | NBR1 | Chemoimmunotherapy | 1.93 | *0.03* |
| JHU15562.B9C6 | SNCAIP | Chemoimmunotherapy | 1.95 | *0.04* |
| JHU18986.B13C32 | CHRDL2 | Chemoimmunotherapy | 1.95 | *0.04* |
| JHU12117.B9C1 | RILPL2 | Chemoimmunotherapy | 1.96 | *0.01* |
| JHU10498.B6C27 | FCRL2 | Chemoimmunotherapy | 1.98 | *0.03* |
| JHU12262.B9C2 | ATN1 | Chemoimmunotherapy | 1.98 | *0.02* |
| JHU04311.B3C15 | TST | Chemoimmunotherapy | 1.98 | *0.04* |
| JHU06198.B6C23 | NANS | Chemoimmunotherapy | 2.00 | *0.02* |
| JHU14340.B12C27 | SLC5A6 | Chemoimmunotherapy | 2.01 | *0.01* |
| JHU12988.B11C31 | PER1 | Chemoimmunotherapy | 2.03 | *0.01* |
| JHU14133.B9C7 | NSL1 | Chemoimmunotherapy | 2.06 | *0.05* |
| JHU03771.B4C13 | CDK2AP2 | Chemoimmunotherapy | 2.06 | *0.04* |
| JHU09127.B8C10 | BZW1 | Chemoimmunotherapy | 2.06 | *0.02* |
| JHU03988.B2C8 | MGC24103 | Chemoimmunotherapy | 2.07 | *0.01* |
| JHU07452.B5C32 | NDRG1 | Chemoimmunotherapy | 2.08 | *0.02* |
| JHU00807.B4C26 | FOSL2 | Chemoimmunotherapy | 2.11 | *0.01* |
| JHU16394.B10C26 | C2orf27A | Chemoimmunotherapy | 2.12 | *0.03* |
| JHU04394.B9C23 | SNX11 | Chemoimmunotherapy | 2.12 | *0.01* |
| JHU14074.B10C4 | ZNF773 | Chemoimmunotherapy | 2.17 | *0.01* |
| JHU01014.B1C24 | MAGEA4 | Chemoimmunotherapy | 2.26 | *<0.01* |
| JHU08741.B8C18 | NACC2 | Chemoimmunotherapy | 2.28 | *0.01* |
| JHU05067.B11C8 | SH2D2A | Chemoimmunotherapy | 2.28 | *0.02* |
| JHU14849.B10C30 | LASP1 | Chemoimmunotherapy | 2.28 | *0.02* |
| JHU11117.B8C30 | SGCD | Chemoimmunotherapy | 2.29 | *0.02* |
| JHU01479.B4C28 | FUBP1 | Chemoimmunotherapy | 2.29 | *0.02* |
| JHU05456.B3C23 | STRAP | Chemoimmunotherapy | 2.30 | *0.03* |
| JHU19676.B16C27 | POU2AF1 | Chemoimmunotherapy | 2.30 | *0.01* |
| JHU07764.B6C15 | STK4_frag | Chemoimmunotherapy | 2.31 | *<0.01* |
| JHU11024.B5C1 | MED22 | Chemoimmunotherapy | 2.33 | *<0.01* |
| JHU12713.B15C21 | THOP1 | Chemoimmunotherapy | 2.34 | *<0.01* |
| JHU04602.B1C30 | TSC22D1 | Chemoimmunotherapy | 2.37 | *0.05* |
| JHU12832.B9C25 | ATXN3 | Chemoimmunotherapy | 2.38 | *0.02* |
| JHU04809.B3C4 | MAPK1IP1L | Chemoimmunotherapy | 2.42 | *0.04* |
| JHU07413.B6C27 | CEP85 | Chemoimmunotherapy | 2.44 | *0.02* |
| JHU05228.B3C1 | IFT57 | Chemoimmunotherapy | 2.45 | *0.04* |
| JHU00829.B2C15 | PCTP | Chemoimmunotherapy | 2.45 | *0.01* |
| JHU03328.B1C4 | NUDCD2 | Chemoimmunotherapy | 2.46 | *0.01* |
| JHU15339.B13C30 | CCDC184 | Chemoimmunotherapy | 2.46 | *0.03* |
| JHU08569.B7C22 | DDB1 | Chemoimmunotherapy | 2.47 | *0.04* |
| JHU06832.B8C31 | LURAP1L | Chemoimmunotherapy | 2.48 | *<0.01* |
| JHU04134.B1C21 | ARL6 | Chemoimmunotherapy | 2.49 | *0.01* |
| JHU02635.B3C30 | IST1 | Chemoimmunotherapy | 2.52 | *0.01* |
| JHU03470.B1C15 | ANAPC15 | Chemoimmunotherapy | 2.52 | *<0.01* |
| JHU18518.B16C7 | UBQLN3 | Chemoimmunotherapy | 2.55 | *0.03* |
| JHU11872.B11C7 | RHBDD1 | Chemoimmunotherapy | 2.58 | *0.02* |
| JHU05547.B1C14 | TEX13A | Chemoimmunotherapy | 2.63 | *0.02* |
| JHU18011.B14C1 | NKTR | Chemoimmunotherapy | 2.66 | *0.02* |
| JHU17319.B13C17 | STK39 | Chemoimmunotherapy | 2.68 | *0.03* |
| JHU01303.B3C27 | MAD2L1 | Chemoimmunotherapy | 2.70 | *<0.01* |
| JHU13236.B9C11 | GGA1 | Chemoimmunotherapy | 2.74 | *0.01* |
| JHU06828.B6C21 | BCL10 | Chemoimmunotherapy | 2.80 | *0.03* |
| JHU02255.B2C7 | MGLL | Chemoimmunotherapy | 2.80 | *0.02* |
| JHU07282.B5C10 | SOCS3 | Chemoimmunotherapy | 2.81 | *0.01* |
| JHU26008.B19C5 | RASSF7 | Chemoimmunotherapy | 2.82 | *0.04* |
| JHU19682.B14C8 | PCGF2 | Chemoimmunotherapy | 2.91 | *0.01* |
| JHU09955.B6C28 | PRRC1 | Chemoimmunotherapy | 2.93 | *0.02* |
| JHU09963.B6C7 | SCHIP1 | Chemoimmunotherapy | 3.00 | *0.04* |
| JHU07242.B8C31 | IL7 | Chemoimmunotherapy | 3.00 | *0.03* |
| JHU29101.B13C25 | ZNF746 | Chemoimmunotherapy | 3.03 | *<0.01* |
| JHU19945.B20C10 | SP4 | Chemoimmunotherapy | 3.07 | *0.03* |
| JHU02734.B3C3 | HSBP1 | Chemoimmunotherapy | 3.12 | *0.05* |
| JHU30058.B18C17 | PPCS | Chemoimmunotherapy | 3.17 | *0.03* |
| JHU14703.B12C32 | VEGFB | Chemoimmunotherapy | 3.23 | *0.04* |
| JHU12621.B9C10 | TBC1D9B | Chemoimmunotherapy | 3.23 | *0.05* |
| JHU03308.B2C11 | MED29 | Chemoimmunotherapy | 3.36 | *<0.01* |
| JHU04617.B1C12 | ATG12 | Chemoimmunotherapy | 3.62 | *0.01* |
| JHU18183.B16C11 | FBLIM1 | Chemoimmunotherapy | 3.72 | *0.02* |
| JHU14241.B9C3 | SET | Chemoimmunotherapy | 3.98 | *0.01* |
| JHU06558.B5C3 | MAPK9 | Chemoimmunotherapy | 5.92 | *<0.01* |
| JHU17971.B15C1 | ATP4A | Chemoimmunotherapy | 12.14 | *<0.01* |
| JHU17172.B15C14 | MYBPC2 | ICIs Monotherapy | 0.19 | *0.01* |
| JHU30129.B17C29 | MACROD2 | ICIs Monotherapy | 0.22 | *0.01* |
| JHU16908.B15C4 | RSPH1 | ICIs Monotherapy | 0.24 | *0.01* |
| JHU17178.B14C32 | PAGE2B | ICIs Monotherapy | 0.25 | *0.02* |
| JHU29573.B18C12 | MGARP | ICIs Monotherapy | 0.28 | *0.01* |
| JHU29793.B19C1 | CCDC117 | ICIs Monotherapy | 0.29 | *0.01* |
| JHU27204.B17C23 | TMOD2 | ICIs Monotherapy | 0.30 | *0.02* |
| JHU29727.B18C29 | ACOT1 | ICIs Monotherapy | 0.31 | *0.02* |
| JHU29499.B20C26 | GGACT | ICIs Monotherapy | 0.31 | *0.01* |
| JHU29696.B18C29 | SETD7 | ICIs Monotherapy | 0.34 | *0.03* |
| JHU27481.B20C20 | OSTF1 | ICIs Monotherapy | 0.34 | *0.01* |
| JHU30116.B20C4 | ANKRD2 | ICIs Monotherapy | 0.34 | *<0.01* |
| JHU29677.B19C9 | NFKBID | ICIs Monotherapy | 0.36 | *0.01* |
| JHU30259.B19C30 | CYP4F2 | ICIs Monotherapy | 0.36 | *0.01* |
| JHU16875.B15C4 | LELP1 | ICIs Monotherapy | 0.37 | *0.01* |
| JHU29771.B18C16 | GPR152 | ICIs Monotherapy | 0.37 | *0.02* |
| JHU30151.B18C16 | PPP4R2 | ICIs Monotherapy | 0.38 | *0.02* |
| JHU29580.B18C20 | H1FNT | ICIs Monotherapy | 0.39 | *0.02* |
| JHU18127.B14C31 | SLC16A2 | ICIs Monotherapy | 0.40 | *0.01* |
| JHU30188.B19C11 | RCSD1 | ICIs Monotherapy | 0.40 | *0.03* |
| JHU30323.B17C21 | SPPL2B | ICIs Monotherapy | 0.41 | *0.01* |
| JHU17031.B15C26 | C9orf139 | ICIs Monotherapy | 0.41 | *0.02* |
| JHU29858.B17C25 | RFTN1 | ICIs Monotherapy | 0.41 | *0.03* |
| JHU16885.B16C11 | MT4 | ICIs Monotherapy | 0.41 | *0.03* |
| JHU29395.B19C24 | CNGA3 | ICIs Monotherapy | 0.42 | *0.02* |
| JHU02653.B4C30 | PEX19 | ICIs Monotherapy | 0.42 | *0.01* |
| JHU05658.B15C24 | VSIG1 | ICIs Monotherapy | 0.42 | *0.01* |
| JHU30125.B19C22 | MESDC1 | ICIs Monotherapy | 0.43 | *0.01* |
| JHU16835.B15C25 | SCIMP | ICIs Monotherapy | 0.44 | *0.02* |
| JHU15086.B11C32 | UCN3 | ICIs Monotherapy | 0.44 | *0.01* |
| JHU16934.B15C1 | CCDC114 | ICIs Monotherapy | 0.44 | *<0.01* |
| JHU30266.B20C21 | ERVW-1 | ICIs Monotherapy | 0.46 | *0.01* |
| JHU19908.B16C26 | ZNF646 | ICIs Monotherapy | 0.46 | *0.01* |
| JHU27153.B17C5 | FAM84B | ICIs Monotherapy | 0.46 | *0.05* |
| JHU18304.B14C14 | PARP1 | ICIs Monotherapy | 0.46 | *0.02* |
| JHU30176.B18C25 | AGXT | ICIs Monotherapy | 0.47 | *0.02* |
| JHU16832.B14C25 | SMCO3 | ICIs Monotherapy | 0.47 | *0.05* |
| JHU16911.B13C25 | SULT1A3 | ICIs Monotherapy | 0.47 | *0.03* |
| JHU29375.B19C6 | PPP1R14D | ICIs Monotherapy | 0.47 | *<0.01* |
| JHU18133.B13C18 | TNRC6C | ICIs Monotherapy | 0.49 | *0.01* |
| JHU19671.B15C29 | PABPN1 | ICIs Monotherapy | 0.49 | *0.04* |
| JHU16899.B13C1 | PSMF1 | ICIs Monotherapy | 0.49 | *0.02* |
| JHU06896.B5C13 | SLC25A5 | ICIs Monotherapy | 0.49 | *0.03* |
| JHU17281.B15C30 | TWF1 | ICIs Monotherapy | 0.50 | *0.03* |
| JHU12684.B15C16 | NCKIPSD | ICIs Monotherapy | 0.50 | *0.02* |
| JHU29973.B18C12 | CDNF | ICIs Monotherapy | 0.50 | *0.01* |
| JHU17403.B14C18 | TXLNA | ICIs Monotherapy | 0.50 | *0.01* |
| JHU08363.B5C10 | MORN4 | ICIs Monotherapy | 0.50 | *0.05* |
| JHU19927.B16C2 | Pdcd11 | ICIs Monotherapy | 0.51 | *0.02* |
| JHU30178.B17C25 | C1orf141 | ICIs Monotherapy | 0.51 | *0.01* |
| JHU05331.B15C11 | MAX | ICIs Monotherapy | 0.51 | *0.01* |
| JHU18697.B15C6 | MKL1 | ICIs Monotherapy | 0.51 | *<0.01* |
| JHU12237.B12C13 | SREBF1 | ICIs Monotherapy | 0.52 | *0.02* |
| JHU15036.B11C17 | SYBU | ICIs Monotherapy | 0.53 | *0.01* |
| JHU17108.B13C5 | ZBTB24 | ICIs Monotherapy | 0.54 | *0.05* |
| JHU08026.B6C7 | OR2S2 | ICIs Monotherapy | 0.55 | *0.04* |
| JHU16858.B19C12 | GAGE12B | ICIs Monotherapy | 0.55 | *0.05* |
| JHU17972.B15C24 | BTBD7 | ICIs Monotherapy | 0.56 | *0.02* |
| JHU15270.B16C10 | TAPBP | ICIs Monotherapy | 0.57 | *0.01* |
| JHU12405.B9C29 | MAPKAPK2 | ICIs Monotherapy | 0.57 | *0.01* |
| JHU30142.B19C19 | HARBI1 | ICIs Monotherapy | 0.57 | *0.03* |
| JHU30073.B18C14 | LRRC73 | ICIs Monotherapy | 0.58 | *0.02* |
| JHU12389.B9C22 | HOXC4 | ICIs Monotherapy | 0.58 | *0.01* |
| JHU02092.B18C21 | SEMG1 | ICIs Monotherapy | 0.58 | *0.03* |
| JHU19694.B15C8 | ZMAT3 | ICIs Monotherapy | 0.58 | *0.03* |
| JHU16142.B12C3 | USP36 | ICIs Monotherapy | 0.58 | *0.02* |
| JHU19770.B14C16 | Repin1 | ICIs Monotherapy | 0.59 | *0.01* |
| JHU17358.B13C21 | MIB2 | ICIs Monotherapy | 0.60 | *0.01* |
| JHU16866.B14C24 | HSPB3 | ICIs Monotherapy | 0.61 | *0.01* |
| JHU17872.B14C19 | AJAP1 | ICIs Monotherapy | 0.61 | *0.01* |
| JHU16282.B11C24 | GOLGA7 | ICIs Monotherapy | 0.61 | *0.02* |
| JHU16039.B11C18 | STAU1 | ICIs Monotherapy | 0.61 | *0.02* |
| JHU16884.B13C25 | MRGPRE | ICIs Monotherapy | 0.61 | *0.04* |
| JHU11692.B12C9 | TARBP2 | ICIs Monotherapy | 0.61 | *0.02* |
| JHU19960.B15C5 | ZFP30 | ICIs Monotherapy | 0.61 | *0.01* |
| JHU30190.B17C27 | PNPLA5 | ICIs Monotherapy | 0.62 | *0.04* |
| JHU18457.B13C28 | HMOX2 | ICIs Monotherapy | 1.52 | *0.02* |
| JHU10701.B15C20 | GATSL2 | ICIs Monotherapy | 1.61 | *0.04* |
| JHU11155.B16C25 | COASY | ICIs Monotherapy | 1.62 | *0.03* |
| JHU15912.B11C4 | METTL21C | ICIs Monotherapy | 1.62 | *0.04* |
| JHU11839.B14C1 | XM_009242791.1_frag | ICIs Monotherapy | 1.63 | *0.01* |
| JHU03554.B2C29 | C11orf68 | ICIs Monotherapy | 1.64 | *0.04* |
| JHU00976.B13C4 | AIDA | ICIs Monotherapy | 1.65 | *0.01* |
| JHU12665.B14C2 | HBS1L | ICIs Monotherapy | 1.65 | *0.02* |
| JHU05345.B3C31 | PPP2R1A | ICIs Monotherapy | 1.66 | *0.02* |
| JHU07968.B5C13 | AHNAK2 | ICIs Monotherapy | 1.68 | *0.04* |
| JHU08467.B5C5 | DPP3 | ICIs Monotherapy | 1.72 | *0.05* |
| JHU04563.B2C7 | MED10 | ICIs Monotherapy | 1.74 | *0.03* |
| JHU11570.B9C5 | OTUB2 | ICIs Monotherapy | 1.74 | *0.01* |
| JHU10388.B6C15 | EP400NL | ICIs Monotherapy | 1.74 | *0.05* |
| JHU04533.B2C18 | CARHSP1 | ICIs Monotherapy | 1.75 | *0.04* |
| JHU29686.B19C16 | KIF25 | ICIs Monotherapy | 1.77 | *0.02* |
| JHU18948.B13C22 | RCN3 | ICIs Monotherapy | 1.79 | *<0.01* |
| JHU05927.B5C17 | RP2 | ICIs Monotherapy | 1.79 | *<0.01* |
| JHU10104.B13C11 | FAM9C | ICIs Monotherapy | 1.80 | *<0.01* |
| JHU07592.B5C12 | C1orf101 | ICIs Monotherapy | 1.81 | *0.02* |
| JHU21865.B15C18 | EPB41L1 | ICIs Monotherapy | 1.82 | *0.04* |
| JHU16942.B14C12 | DAPK3 | ICIs Monotherapy | 1.83 | *0.03* |
| JHU16752.B14C11 | MLXIP | ICIs Monotherapy | 1.84 | *0.03* |
| JHU04886.B2C19 | TWF1 | ICIs Monotherapy | 1.84 | *0.01* |
| JHU17279.B13C6 | TLK1 | ICIs Monotherapy | 1.86 | *0.02* |
| JHU18358.B13C25 | FLAD1 | ICIs Monotherapy | 1.86 | *0.03* |
| JHU04900.B12C29 | ANXA10 | ICIs Monotherapy | 1.86 | *0.04* |
| JHU13960.B9C8 | PITPNA | ICIs Monotherapy | 1.86 | *0.03* |
| JHU11140.B5C12 | BLOC1S2 | ICIs Monotherapy | 1.88 | *<0.01* |
| JHU10451.B5C12 | TAPT1 | ICIs Monotherapy | 1.91 | *0.02* |
| JHU00436.B13C16 | UBXN1 | ICIs Monotherapy | 1.92 | *0.01* |
| JHU11023.B6C4 | SMYD2 | ICIs Monotherapy | 1.93 | *<0.01* |
| JHU13759.B14C5 | PPP1R1B | ICIs Monotherapy | 1.94 | *0.01* |
| JHU04595.B2C18 | TANK | ICIs Monotherapy | 2.00 | *0.02* |
| JHU08393.B6C13 | ILKAP | ICIs Monotherapy | 2.00 | *<0.01* |
| JHU18237.B13C2 | YARS | ICIs Monotherapy | 2.01 | *<0.01* |
| JHU02774.B3C3 | SNRNP70 | ICIs Monotherapy | 2.03 | *0.01* |
| JHU00426.B14C1 | HDAC3 | ICIs Monotherapy | 2.03 | *0.02* |
| JHU00465.B2C15 | TCEAL1 | ICIs Monotherapy | 2.04 | *<0.01* |
| JHU02539.B1C4 | BABAM1 | ICIs Monotherapy | 2.04 | *0.02* |
| JHU19056.B16C2 | SPACA7 | ICIs Monotherapy | 2.04 | *0.03* |
| JHU04590.B2C31 | SSFA2_frag | ICIs Monotherapy | 2.05 | *0.03* |
| JHU05493.B4C32 | CTBP2 | ICIs Monotherapy | 2.06 | *0.03* |
| JHU25302.B17C4 | AKT1 | ICIs Monotherapy | 2.06 | *0.02* |
| JHU08885.B6C3 | MAP4 | ICIs Monotherapy | 2.16 | *0.02* |
| JHU04290.B16C9 | PPIE | ICIs Monotherapy | 2.19 | *0.02* |
| JHU10699.B6C13 | C2orf74 | ICIs Monotherapy | 2.21 | *0.04* |
| JHU05401.B2C15 | GPR119 | ICIs Monotherapy | 2.26 | *0.05* |
| JHU13904.B13C29 | CRNN | ICIs Monotherapy | 2.37 | *0.01* |
| JHU05029.B4C26 | FKBP3 | ICIs Monotherapy | 2.63 | *<0.01* |
| JHU08814.B7C13 | SPR | ICIs Monotherapy | 2.81 | *<0.01* |
| JHU29725.B19C6 | PPP4R2 | Same name with dif ID | 0.26 | *0.02* |
| JHU11081.B8C18 | MED29 | Same name with dif ID | 2.06 | *<0.01* |
| JHU15263.B9C7 | SCHIP1 | Same name with dif ID | 2.44 | *0.03* |
| JHU16448.B11C12 | MAD2L1 | Same name with dif ID | 2.59 | *<0.01* |
| JHU05905.B5C17 | MAP4 | Same name with dif ID | 2.60 | *0.02* |
| JHU19614.B13C8 | RASSF7 | Same name with dif ID | 2.67 | *0.03* |
| JHU20149.B17C2 | GGA1 | Same name with dif ID | 3.18 | *0.02* |
| Auto-antigen.B20C16R38 | Influenza A M2 | Dynamic | N/A | *N/A* |
| JHU00063.B2C5 | PDK3 | Dynamic | N/A | *N/A* |
| JHU00198.B2C6 | ARL17A_frag | Dynamic | N/A | *N/A* |
| JHU00735.B1C28 | RAB3C | Dynamic | N/A | *N/A* |
| JHU00913.B2C32 | LDB3 | Dynamic | N/A | *N/A* |
| JHU01220.B2C15 | SCARB2 | Dynamic | N/A | *N/A* |
| JHU01753.B14C21 | CD300LG | Dynamic | N/A | *N/A* |
| JHU01911.B18C26 | NELFCD | Dynamic | N/A | *N/A* |
| JHU02270.B2C18 | PPARA | Dynamic | N/A | *N/A* |
| JHU02690.B3C26 | ACTB | Dynamic | N/A | *N/A* |
| JHU02759.B3C15 | PPA2_frag | Dynamic | N/A | *N/A* |
| JHU02894.B4C19 | CHMP1B | Dynamic | N/A | *N/A* |
| JHU02930.B2C24 | MRPL19 | Dynamic | N/A | *N/A* |
| JHU03133.B2C15 | PLEK2 | Dynamic | N/A | *N/A* |
| JHU03319.B3C19 | C5orf46 | Dynamic | N/A | *N/A* |
| JHU03403.B3C31 | KPTN | Dynamic | N/A | *N/A* |
| JHU03460.B4C20 | AGPAT3_frag | Dynamic | N/A | *N/A* |
| JHU03559.B4C22 | LINC00526 | Dynamic | N/A | *N/A* |
| JHU03711.B4C11 | NSMCE2 | Dynamic | N/A | *N/A* |
| JHU03863.B1C23 | CHD9_frag | Dynamic | N/A | *N/A* |
| JHU04273.B4C20 | C16orf89 | Dynamic | N/A | *N/A* |
| JHU04283.B1C23 | NIPA2 | Dynamic | N/A | *N/A* |
| JHU04485.B3C1 | SGOL1 | Dynamic | N/A | *N/A* |
| JHU04554.B1C18 | IFI27 | Dynamic | N/A | *N/A* |
| JHU04556.B4C7 | IL1B | Dynamic | N/A | *N/A* |
| JHU04640.B4C12 | GNG10 | Dynamic | N/A | *N/A* |
| JHU04764.B4C32 | PSMC4 | Dynamic | N/A | *N/A* |
| JHU04870.B1C25 | SCAMP4 | Dynamic | N/A | *N/A* |
| JHU04906.B15C24 | C15orf41 | Dynamic | N/A | *N/A* |
| JHU05148.B3C22 | PRMT6 | Dynamic | N/A | *N/A* |
| JHU05257.B3C1 | SCO1 | Dynamic | N/A | *N/A* |
| JHU05350.B2C21 | RPL18 | Dynamic | N/A | *N/A* |
| JHU05361.B20C14 | STARD4 | Dynamic | N/A | *N/A* |
| JHU05895.B8C14 | GPATCH2 | Dynamic | N/A | *N/A* |
| JHU05941.B5C29 | TMEM120A | Dynamic | N/A | *N/A* |
| JHU06329.B12C28 | TFB2M | Dynamic | N/A | *N/A* |
| JHU07221.B15C19 | CYSLTR2 | Dynamic | N/A | *N/A* |
| JHU07645.B6C20 | NPY5R | Dynamic | N/A | *N/A* |
| JHU07970.B5C30 | ANKRD49 | Dynamic | N/A | *N/A* |
| JHU08510.B8C15 | SEZ6L2 | Dynamic | N/A | *N/A* |
| JHU08730.B11C13 | VPS13B_frag | Dynamic | N/A | *N/A* |
| JHU08745.B6C10 | SARG | Dynamic | N/A | *N/A* |
| JHU08751.B6C31 | GLIPR2 | Dynamic | N/A | *N/A* |
| JHU08978.B5C17 | MTF2 | Dynamic | N/A | *N/A* |
| JHU09132.B5C28 | CCDC115_frag | Dynamic | N/A | *N/A* |
| JHU09729.B5C15 | HESX1 | Dynamic | N/A | *N/A* |
| JHU10082.B7C14 | ASB4 | Dynamic | N/A | *N/A* |
| JHU10094.B8C14 | CTDSP2 | Dynamic | N/A | *N/A* |
| JHU10262.B8C19 | TRIM60 | Dynamic | N/A | *N/A* |
| JHU10707.B5C31 | MPHOSPH9 | Dynamic | N/A | *N/A* |
| JHU10715.B8C18 | PRDX5 | Dynamic | N/A | *N/A* |
| JHU10720.B5C7 | RORA | Dynamic | N/A | *N/A* |
| JHU10727.B8C10 | SPATA12 | Dynamic | N/A | *N/A* |
| JHU10754.B7C2 | AQP8 | Dynamic | N/A | *N/A* |
| JHU10897.B7C8 | LYNX1 | Dynamic | N/A | *N/A* |
| JHU11706.B15C30 | TLX2_frag | Dynamic | N/A | *N/A* |
| JHU11750.B5C7 | ITGA6 | Dynamic | N/A | *N/A* |
| JHU11757.B10C6 | VWA5A | Dynamic | N/A | *N/A* |
| JHU11952.B12C23 | MAP3K7 | Dynamic | N/A | *N/A* |
| JHU12484.B10C24 | ERN1 | Dynamic | N/A | *N/A* |
| JHU12487.B11C1 | GNG8 | Dynamic | N/A | *N/A* |
| JHU12566.B10C28 | DOK3 | Dynamic | N/A | *N/A* |
| JHU12796.B12C8 | PRRX2 | Dynamic | N/A | *N/A* |
| JHU12889.B9C11 | PMF1 | Dynamic | N/A | *N/A* |
| JHU12981.B6C32 | NR6A1 | Dynamic | N/A | *N/A* |
| JHU13032.B12C12 | CCL22 | Dynamic | N/A | *N/A* |
| JHU13047.B9C5 | FAS | Dynamic | N/A | *N/A* |
| JHU13055.B9C23 | FAM110D | Dynamic | N/A | *N/A* |
| JHU13068.B11C26 | LRP11 | Dynamic | N/A | *N/A* |
| JHU13081.B9C5 | NKX2-8 | Dynamic | N/A | *N/A* |
| JHU13187.B11C9 | PSMD14 | Dynamic | N/A | *N/A* |
| JHU13291.B10C25 | SLC6A16 | Dynamic | N/A | *N/A* |
| JHU13363.B9C18 | NUB1 | Dynamic | N/A | *N/A* |
| JHU13558.B12C8 | RAB3IP | Dynamic | N/A | *N/A* |
| JHU13992.B12C4 | CASQ1 | Dynamic | N/A | *N/A* |
| JHU14410.B9C4 | SPAG11A | Dynamic | N/A | *N/A* |
| JHU14590.B16C28 | RHOQ | Dynamic | N/A | *N/A* |
| JHU14723.B3C14 | FERMT1 | Dynamic | N/A | *N/A* |
| JHU15091.B10C29 | ZNF182 | Dynamic | N/A | *N/A* |
| JHU15409.B20C29 | FGFR2 | Dynamic | N/A | *N/A* |
| JHU15466.B5C29 | TRIM27 | Dynamic | N/A | *N/A* |
| JHU15913.B10C11 | C11orf85 | Dynamic | N/A | *N/A* |
| JHU16103.B11C23 | LARP7 | Dynamic | N/A | *N/A* |
| JHU16107.B10C2 | ZNF385C_frag | Dynamic | N/A | *N/A* |
| JHU16190.B12C17 | C5AR2 | Dynamic | N/A | *N/A* |
| JHU16827.B13C25 | ANKRD19P | Dynamic | N/A | *N/A* |
| JHU16850.B16C16 | CXorf40A | Dynamic | N/A | *N/A* |
| JHU17037.B16C15 | TMEM257 | Dynamic | N/A | *N/A* |
| JHU17094.B13C5 | SYT14 | Dynamic | N/A | *N/A* |
| JHU17115.B16C17 | ALDH18A1 | Dynamic | N/A | *N/A* |
| JHU17190.B16C14 | RXFP1 | Dynamic | N/A | *N/A* |
| JHU17691.B13C31 | ANO10 | Dynamic | N/A | *N/A* |
| JHU18753.B17C2 | CNGA1 | Dynamic | N/A | *N/A* |
| JHU18908.B16C29 | GNRH1 | Dynamic | N/A | *N/A* |
| JHU24557.B17C29 | ZFC3H1 | Dynamic | N/A | *N/A* |
| JHU24711.B18C17 | SEPT8 | Dynamic | N/A | *N/A* |
| JHU25609.B19C11 | VWF | Dynamic | N/A | *N/A* |
| JHU25628.B18C4 | PDS5B | Dynamic | N/A | *N/A* |
| JHU25735.B18C28 | AKAP13 | Dynamic | N/A | *N/A* |
| JHU26155.B19C23 | ZFP69B | Dynamic | N/A | *N/A* |
| JHU26473.B19C20 | ACADSB | Dynamic | N/A | *N/A* |
| JHU27978.B20C24 | BCL7A | Dynamic | N/A | *N/A* |
| JHU29108.B7C23 | AK9 | Dynamic | N/A | *N/A* |
| JHU29142.B20C11 | ITGA4B7 | Dynamic | N/A | *N/A* |
| JHU29292.B17C8 | ZNF544 | Dynamic | N/A | *N/A* |
| JHU29350.B18C24 | DIRC1 | Dynamic | N/A | *N/A* |
| JHU29373.B19C17 | C2orf83 | Dynamic | N/A | *N/A* |
| JHU29392.B18C25 | CD180 | Dynamic | N/A | *N/A* |
| JHU29428.B18C15 | TLR10 | Dynamic | N/A | *N/A* |
| JHU29475.B20C10 | LIG3 | Dynamic | N/A | *N/A* |
| JHU29501.B19C20 | DAOA | Dynamic | N/A | *N/A* |
| JHU29553.B20C20 | CMA1 | Dynamic | N/A | *N/A* |
| JHU29604.B18C20 | PHF13 | Dynamic | N/A | *N/A* |
| JHU29635.B17C5 | CYYR1 | Dynamic | N/A | *N/A* |
| JHU29880.B18C31 | C19orf57 | Dynamic | N/A | *N/A* |
| JHU29958.B18C26 | DLEU7 | Dynamic | N/A | *N/A* |
| JHU29985.B20C14 | C9orf40 | Dynamic | N/A | *N/A* |
| JHU30127.B18C27 | TTC41P | Dynamic | N/A | *N/A* |
| JHU30211.B19C25 | ONECUT1 | Dynamic | N/A | *N/A* |
| JHU30350.B20C23 | SYNPO2L | Dynamic | N/A | *N/A* |
| JHU30379.B20C23 | BICD2 | Dynamic | N/A | *N/A* |
| JHU30382.B18C26 | SLC9A5 | Dynamic | N/A | *N/A* |
| JHU14968.B10C12 | SET | Dynamic | N/A | *N/A* |
| JHU00746.B2C13R11 | SIX2 | Literature | N/A | *N/A* |
| JHU02721.B3C20R48 | EIF4E2 | Literature | N/A | *N/A* |
| JHU04788.B3C9R75 | TP53 | Literature | N/A | *N/A* |
| JHU15310.B9C23R61 | EIF4E2 | Literature | N/A | *N/A* |
| JHU15642.B11C13R63 | PDCD1 | Literature | N/A | *N/A* |
| JHU16843.B13C11R25 | CD274 | Literature | N/A | *N/A* |
| JHU00287.B14C24 | TRIM21 | Pos-control | N/A | *N/A* |
| JHU01175.B13C10 | FAM160B2 | Pos-control | N/A | *N/A* |
| JHU02846.B3C29 | RNF7 | Pos-control | N/A | *N/A* |
| JHU03021.B13C29 | KV205 | Pos-control | N/A | *N/A* |
| JHU15174.B12C11 | SIRPB1 | Pos-control | N/A | *N/A* |
| JHU15253.B11C31 | PDK2 | Pos-control | N/A | *N/A* |

*Abbreviation: AAbs: autoantibodies; ICIs: immune checkpoint inhibitors; aNSCLC: advanced non-small cell lung cancer.*

**Table S4. Differential AAbs identified between responder and non-responder patients before (*n*=65) and after (*n*=50) chemoimmunotherapy through “limma” analysis in the verification cohort and the corresponding fold changes in the discovery phase (*p* < 0.05).**

| **Non-Responder vs. Responder** | | | | | | | |
| --- | --- | --- | --- | --- | --- | --- | --- |
| **Baseline (19AAbs)** | | | | **T1 (27AAbs)** | | | |
| **ID** | **Name** | **FC** | ***p*** | **ID** | **Name** | **FC** | ***p*** |
| JHU12333P129G12 | SYNJ2 | 1.442 | *0.003* | JHU04788P050D05 | TP53 | 2.677 | *0.045* |
| JHU14968P157E10 | SET_1 | 1.386 | *0.045* | JHU06558P069H11 | MAPK9 | 2.229 | *0.006* |
| JHU11491P120H11 | PPP6R2 | 1.366 | *0.014* | JHU18183P239B02 | FBLIM1 | 2.098 | *0.002* |
| JHU10682P112E03 | RILPL1 | 1.335 | *0.047* | JHU06828P072F03 | BCL10 | 1.943 | *0.007* |
| JHU07242P076B12 | IL7 | 1.312 | *0.043* | JHU17971P223D01 | ATP4A | 1.933 | *0.029* |
| JHU10989P115C10 | LRFN1 | 1.29 | *0.032* | JHU11297P118G02 | SEC24C | 1.875 | *0.003* |
| JHU09955P104E04 | PRRC1 | 1.289 | *0.022* | JHU12832P135H04 | ATXN3 | 1.833 | *0* |
| JHU19676P182B08 | POU2AF1 | 1.265 | *0.032* | JHU13236P139H02 | GGA1_1 | 1.731 | *0.008* |
| JHU11024P115H09 | MED22 | 1.243 | *0.05* | JHU04556P048B05 | IL1B | 1.692 | *0.024* |
| JHU01303P014D08 | MAD2L1_1 | 1.229 | *0.042* | JHU12621P132G05 | TBC1D9B | 1.642 | *0.038* |
| JHU10987P115A11 | FAM231D | 1.221 | *0.042* | JHU02255P024F05 | MGLL | 1.544 | *0.005* |
| JHU25682P243E09 | SYCE2 | 1.207 | *0.035* | JHU07413P078F12 | CEP85 | 1.431 | *0.021* |
| JHU16394P173E12 | C2orf27A | 1.206 | *0.026* | JHU04602P048G10 | TSC22D1 | 1.414 | *0.039* |
| JHU15270P160B02 | TAPBP | 0.832 | *0.049* | JHU07452P078G08 | NDRG1 | 1.405 | *0.036* |
| JHU14294P150C09 | DHX29 | 0.831 | *0.047* | JHU09127P096A01 | BZW1 | 1.335 | *0.034* |
| JHU17088P201C12 | RNASE9 | 0.83 | *0.045* | JHU12903P135G03 | SPANXD | 1.287 | *0.041* |
| JHU15974P168D02 | CYP11B1 | 0.819 | *0.01* | JHU00287P235E08 | TRIM21 | 1.166 | *0.019* |
| JHU05331P227D02 | MAX | 0.774 | *0.02* | JHU08067P228C11 | XIAP | 0.818 | *0.031* |
| JHU16467P173F05 | SCYL1 | 0.773 | *0.029* | JHU17078P201A09 | PIK3C2A | 0.816 | *0.041* |
|  |  |  |  | JHU17358P204A03 | MIB2 | 0.784 | *0.011* |
|  |  |  |  | JHU19671P182D02 | PABPN1 | 0.776 | *0.022* |
|  |  |  |  | JHU15270P160B02 | TAPBP | 0.749 | *0.016* |
|  |  |  |  | JHU09360P098B08 | LATS1 | 0.744 | *0.022* |
|  |  |  |  | JHU16467P173F05 | SCYL1 | 0.687 | *0.006* |
|  |  |  |  | JHU05331P227D02 | MAX | 0.676 | *0.045* |
|  |  |  |  | JHU13222P139B11 | CHTF8 | 0.617 | *0.001* |
|  |  |  |  | JHU24727P243A08 | MTMR10 | 0.588 | *0.001* |

*Abbreviation: AAbs: autoantibodies; Responder: patients achieving complete remission, partial remission, or stable disease within three months of treatment; Non-responder: patients achieving disease progression within three months of treatment; aNSCLC: advanced non-small cell lung cancer; T1: time point within three months treatment; FC: fold change.*

**Table S5. Survival analysis of 507 AAbs (only significant AAbs are presented) for PFS before (*n*=65) and after (*n*=50) chemoimmunotherapy in the verification cohort.**

| **Uni-variate COX for PFS** | | | | | | | |
| --- | --- | --- | --- | --- | --- | --- | --- |
| **Baseline (8AAbs)** | | | | **T1 (9AAbs)** | | | |
| **Name** | **HR(95%CI)** | **FC** | ***P Value*** | **Name** | **HR(95%CI)** | **FC** | ***P Value*** |
| DHX29 | 0.22(0.08-0.58) | 0.83 | *0.002* | **MAX** | 0.08（0.02-0.3） | 0.68 | *<0.001* |
| **MAX** | 0.26(0.10-0.67) | 0.77 | *0.005* | **SCYL1** | 0.29（0.1-0.89） | 0.69 | *0.03* |
| CYP11B1 | 0.30(0.12-0.72) | 0.82 | *0.007* | LATS1 | 0.35（0.12-0.96） | 0.74 | *0.042* |
| **SCYL1** | 0.3(0.1-0.87) | 0.77 | *0.027* | TAPBP | 0.32（0.11-0.91） | 0.75 | *0.033* |
| MAD2L1 | 2.62(1.20-5.71) | 1.23 | *0.015* | MIB2 | 0.19（0.06-0.66） | 0.78 | *0.009* |
| MED22 | 3.04(1.45-6.37) | 1.24 | *0.003* | PIK3C2A | 0.15（0.04-0.55） | 0.82 | *0.004* |
| SYNJ2 | 3.14(1.34-7.36) | 1.44 | *0.008* | NDRG1 | 3.65（1.02-12.99） | 1.41 | *0.046* |
| PPP6R2 | 5.35(2.31-12.36) | 1.37 | *<0.001* | BCL10 | 5.88（1.92-18.03） | 1.94 | *0.002* |
|  |  |  |  | TP53 | 3.93（1.28-12.1） | 2.68 | *0.017* |

*Abbreviation: PFS: progression-free survival; aNSCLC: advanced non-small cell lung cancer; ICIs: immune checkpoint inhibitors; T1: time point within three months treatment; HR: hazard risk; CI: confidence interval; FC: fold change.*

**Table S6. Dynamic clusters for 507 AAbs and their enrichment of KEGG pathways in the verification cohort.**

| **Cluster1** | **Cluster2** | **Cluster3** | **Cluster4** |
| --- | --- | --- | --- |
| Human IgG | Human IgM | FOSL2 | anti-h-IgG |
| AASDHPPT | PDK3 | PCTP | Influenza A M2 |
| FAM160B2 | DUSP26 | LDB3 | SCL-70 |
| SCARB2 | TRIM21 | MAD2L1 | Sm/RNP Complex |
| SNAI2 | HDAC3 | CD300LG | Cy3/Cy5 |
| SEMG1 | UBXN1 | SLC5A6 | GST |
| CHMP1B | TCEAL1 | MPHOSPH6 | SKAP2 |
| MRPL19 | RAB3C | PPARA | ARL17A_frag |
| PLEK2 | SIX2 | BABAM1 | DNTT |
| CASS4 | MED28 | PEX19 | AIDA |
| LINC00526 | MAGEA4 | ACTB | DXO |
| NSMCE2 | NELFCD | EIF4E2 | FUBP1 |
| CHD9_frag | ASPH | KV205 | PABPC3 |
| CARHSP1 | CFLAR | C5orf46 | POLR2E |
| MED10 | IST1 | KPTN | MGLL |
| ATG12 | SNX11 | ANAPC15 | HSBP1 |
| GNG10 | IFI27 | MGC24103 | SNRNP70 |
| MAPK1IP1L | IL1B | CFAP43 | RNF7 |
| ARL11 | SSFA2_frag | C16orf89 | MED29 |
| SH2D2A | SCO1 | NIPA2 | NUDCD2 |
| PRMT6 | BCL10 | PPIE | AGPAT3_frag |
| G3BP1 | SLC25A5 | TST | C11orf68 |
| ZFP82 | SOCS3 | COMT | CDK2AP2 |
| GPATCH2 | C1orf101 | PSMC4 | FGFR1OP2 |
| MAP3K11 | AHNAK2 | TWF1 | NDUFS7 |
| IL7 | XIAP | ANXA10 | ANXA6 |
| STK4_frag | FAM29A | C15orf41 | ARL6 |
| MTF2 | DPP3 | MAX | SCCPDH |
| LATS1 | SEZ6L2 | RPL18 | SGOL1 |
| SCHIP1 | VPS13B_frag | STARD4 | TANK |
| ASB4 | IGL@ | STRAP | TSC22D1 |
| PPP6R2 | CTDSP2 | RP2 | TP53 |
| VWA5A | FAM9C | NANS | SCAMP4 |
| XM_009242791.1_frag | TRIM60 | LURAP1L | FKBP3 |
| ATN1 | ZC2HC1A | CYSLTR2 | IFT57 |
| SYNJ2 | C2orf74 | PLPP1 | PPP2R1A |
| NCKIPSD | GATSL2 | NDRG1 | CD68 |
| PMF1 | PRDX5 | EIF4ENIF1 | GPR119 |
| NR6A1 | FAM231D | NPY5R | CTBP2 |
| PSMD14 | MED22 | ANKRD49 | TEX13A |
| C20orf24 | SGCD | MORN4 | VSIG1 |
| SET | TLX2_frag | ABTB1 | TMEM120A |
| SNAI1 | ITGA6 | SARG | DDX28 |
| CCAR2 | RILPL2 | GLIPR2 | TFB2M |
| PDPN | SREBF1 | CCDC115_frag | CD53 |
| PUDP | DOK3 | HESX1 | MAPK9 |
| ZNF268 | ATXN3 | FCRL2 | NAALADL2 |
| SYBU | ZNF655_frag | SMPD3 | CEP85 |
| CLIC2 | ZBTB22 | SPATA12 | DSE |
| PRDM15 | FAM110D | AQP8 | CCNJL_frag |
| SCHIP1 | KAT8 | SMYD2 | OR2S2 |
| TAPBP | NKX2-8 | BLOC1S2 | ILKAP |
| DDX1 | GGA1 | SEC24C | DDB1 |
| TRIM27 | RAB3IP | OTUB2 | NACC2 |
| SNCAIP | DHX29 | TARBP2 | SPR |
| ZIK1 | RGCC | MAPKAPK2 | MAP4 |
| STAU1 | LASP1 | TBC1D9B | MAP4 |
| SRA1_frag | UCN3 | PLEKHH3 | LIMS1 |
| C2orf27A | EIF4E2 | PRRX2 | BZW1 |
| MAD2L1 | ZNF571 | SPANXD | NKRF |
| NG_015859.1_frag | PDCD1 | FAS | PRRC1 |
| ZNF546 | METTL21C | CHTF8 | EP400NL |
| MLXIP | EEF1A1_frag | SLC6A16 | TAPT1 |
| DAPK3 | GLP2R | RHOQ | AGO4 |
| ZNF517 | LARP7 | FERMT1 | RILPL1 |
| PSKH2 | USP36 | SET | MPHOSPH9 |
| ZBTB24 | HMGA1_frag | SIRPB1 | RORA |
| TLK1 | ANKRD19P | PDK2 | LYNX1 |
| ZNF17 | TMEM257 | FGFR2 | LRFN1 |
| SMC6 | ALDH18A1 | AP1G1 | MED29 |
| CRYL1 | PML | OR5D16 | COASY |
| PABPN1 | BTBD7 | RAP1GDS1 | MAATS1 |
| DMTF1 | NKTR | ZNF385C_frag | RHBDD1 |
| U2SURP | B3GAT1 | SH3D19 | MAP3K7 |
| ZNF132 | HMOX2 | GOLGA7 | HOXC4 |
| EPB41L1 | TXNDC2 | BC073758 | ERN1 |
| CCDC158 | EPX | TRAC | GNG8 |
| AKAP13 | MKL1 | SMCO3 | HBS1L |
| ZFP69B | TCTN3 | SCIMP | THOP1 |
| ACADSB | GNRH1 | CD274 | LMF2 |
| ITGA4B7 | HAPLN2 | CXorf40A | PER1 |
| DIRC1 | CYP11B2 | GAGE12B | CCL22 |
| ZMIZ2 | NEK5 | HSPB3 | LRP11 |
| LIG3 | Repin1 | LELP1 | NUB1 |
| CMA1 | ZNF646 | MRGPRE | KLHDC8A |
| NFKBID | ABHD16B | MT4 | ZNF766 |
| CDNF | MBNL3 | PSMF1 | PPP1R1B |
| FBXO16 | DNAJC11 | RSPH1 | CRNN |
| LRRC73 | VEGF | SULT1A3 | NBR1 |
| MESDC1 | ZNF544 | CCDC114 | PITPNA |
| MACROD2 | DAOA | CHAT | CASQ1 |
| AGXT | CYYR1 | C9orf139 | ZNF773 |
| RCSD1 | PPP4R2 | PIK3C2A | NSL1 |
| ACP7 | GPR152 | SYT14 | SPATA5L1_frag |
| TRIM50 | CCDC117 | PAGE2B | AGO1 |
| ERVW-1 | C19orf57 | RXFP1 | LPIN1 |
| PDE10A | MOGAT2 | MIB2 | SPAG11A |
|  | HARBI1 | TXLNA | SRP19 |
|  | PPP4R2 | AJAP1 | VEGFB |
|  | PNPLA5 | L3MBTL2 | ZNF140 |
|  | SPPL2B | PARP1 | ZNF256 |
|  | BICD2 | SF3A2 | ZNF182 |
|  |  | UBQLN3 | CHTF18 |
|  |  | CCL16 | EHHADH |
|  |  | SPACA7 | CCDC184 |
|  |  | ZMAT3 | AGBL2 |
|  |  | Pdcd11 | C11orf85 |
|  |  | ZFC3H1 | CYP11B1 |
|  |  | SEPT8 | MPV17L2 |
|  |  | PDS5B | USP21 |
|  |  | FAM84B | C5AR2 |
|  |  | TMOD2 | IL17F |
|  |  | AK9 | SCYL1 |
|  |  | PPP1R14D | ZKSCAN8 |
|  |  | GGACT | ZNF774 |
|  |  | MGARP | PRDM13 |
|  |  | H1FNT | ZNF768 |
|  |  | RDM1 | RNASE9 |
|  |  | SETD7 | MYBPC2 |
|  |  | ACOT1 | CENPBD1 |
|  |  | RFTN1 | PSTK |
|  |  | DLEU7 | TWF1 |
|  |  | ANKRD2 | STK39 |
|  |  | CYP4F2 | ANO10 |
|  |  | PRPF40B | ATP4A |
|  |  | SYNPO2L | SOCS7 |
|  |  |  | ZNF197 |
|  |  |  | SLC16A2 |
|  |  |  | TNRC6C |
|  |  |  | FBLIM1 |
|  |  |  | YARS |
|  |  |  | CILP |
|  |  |  | FLAD1 |
|  |  |  | ADNP2 |
|  |  |  | CNGA1 |
|  |  |  | KCNH4 |
|  |  |  | OLFML1 |
|  |  |  | RCN3 |
|  |  |  | CHRDL2 |
|  |  |  | EMILIN3 |
|  |  |  | AMH |
|  |  |  | KDM7A |
|  |  |  | TLK2 |
|  |  |  | RASSF7 |
|  |  |  | POU2AF1 |
|  |  |  | PCGF2 |
|  |  |  | Glis2 |
|  |  |  | ZNF219 |
|  |  |  | SNAI3 |
|  |  |  | SP4 |
|  |  |  | ZFP30 |
|  |  |  | Q4vad4 |
|  |  |  | GGA1 |
|  |  |  | PISD |
|  |  |  | RNF180 |
|  |  |  | AGBL5 |
|  |  |  | DHX30 |
|  |  |  | MTMR10 |
|  |  |  | AKT1 |
|  |  |  | AKT1 |
|  |  |  | POU3F3 |
|  |  |  | VWF |
|  |  |  | GALR3 |
|  |  |  | SYCE2 |
|  |  |  | N4BP2L1 |
|  |  |  | RASSF7 |
|  |  |  | OSTF1 |
|  |  |  | BCL7A |
|  |  |  | ZNF266 |
|  |  |  | ZNF746 |
|  |  |  | ZNF814 |
|  |  |  | C2orf83 |
|  |  |  | CD180 |
|  |  |  | CNGA3 |
|  |  |  | TLR10 |
|  |  |  | PHF13 |
|  |  |  | KIF25 |
|  |  |  | DEDD2 |
|  |  |  | IQCF3 |
|  |  |  | C9orf40 |
|  |  |  | PPCS |
|  |  |  | TTC41P |
|  |  |  | C1orf141 |
|  |  |  | ONECUT1 |
|  |  |  | SLC9A5 |

*Abbreviation: AAbs: autoantibodies; KEGG: Kyoto Encyclopedia of Genes And Genomes.*

**Table S7. The autoantibody markers reported in the literature associated with therapeutic and prognosis efficacy in NSCLC.**

| **Treatment** | **Cancer**  **type** | ***n*** | **Autoantibodies** | **Prognostic endpoint** | ***Ref.*** |
| --- | --- | --- | --- | --- | --- |
| Anti-PD-1  therapy | NSCLC | 137 | ANA, thyroglobulin, thyroid peroxidase | PFS | *[1]* |
|  |  | 42 | lgM-RF | PD, PFS, OS | *[2]* |
|  |  | 88 | NY-ESO-1, XAGE1 | PFS, OS | *[3]* |
|  |  | 166 | NY-ESO-1, p53, BRCA2, HUD, TRIM21 | ORR, PFS | *[4]* |
|  |  | 74 | SIX2, EIF4E2 | PD/Non-PD | *[5]* |
|  |  | 108 | anti-PD1 IgG4 | OS | *[6]* |
| Surgery | NSCLC | 157 | SPATA19, TSPY3, GLS2, TCEA2, TSGA10, HMGN5, LUZP4, HDAC4, SPACA3, IMPDH1, TXN2, TFG, PPP2R1A | OS | *[7]* |
|  | Lung  adenocarcinoma | 5 | LSP1, SPP1, PVALB, RGS20,SNRPA,CDH12 | Recurrence | *[8]* |
| Surgery or chemotherapy | NSCLC | 264 | CCNY | OS | *[9]* |
| Surgery, chemotherapy, or radiotherapy | NSCLC | 188 | p53 | OS | *[10]* |
|  |  | 127 | TOPO48 | OS | *[11]* |
|  |  | 177 | p53, SOX2, PGP9.5, MAGEA1 | PFS, OS | *[12]* |

*Abbreviation: PD-1: programmed cell death 1; AAb: autoantibodies; NSCLC: non-*

*small cell lung cancer; PD/Non-PD: disease-progression/non-progression; PFS: progression-free survival; OS: overall survival; ANA: antinuclear antibodies; lgM-RF: immunoglobulin M-rheumatoid factors; NY-ESO-1: new york esophageal squamous cell carcinoma 1; XAGE1: tumor testicular antigen; Tp53: cellular tumor antigen p53; BRCA2: breast cancer type 2 susceptibility protein; HuD: neuro-associated antibody; TRIM21: Tripartite motif containing-21; SIX2: homeobox protein SIX2; EIF4E2: eukaryotic translation initiation factor 4E type; SPATA19: Sperm atogenesis-associated protein 19; TSPY3: testis-specificy-encoded protein 3; GLS2: glutaminase liver isoform, mitochondrial; TCEA2: transcription elongation factor a protein 2; TSGA10: testis specific 10; HMGN5: high mobility group nucleosome binding domain 5; LUZP4: leucine zipper protein 4; HDAC4: histone deacetylase 4; SPACA3: sperm acrosome associated 3; IMPDH1: inosine monophosphate dehydrogenase 1; TXN2: thioredoxin 2; TFG: trafficking from er to golgi regulator; PPP2R1A: protein phosphatase 2 scaffold subunit aalpha; LSP1: lymphocyte specific protein 1; SPP1: Secreted phosphoprotein 1; PVALB: parvalbumin; RGS20: regulator of G protein signaling 20; SNRPA: small nuclear ribonucleoprotein polypeptide A; CDH12: cadherin 12; CCNY: cyclin Y; TOPO48: DNA topoisomerase I; SOX2: Sex determining region Y-box 2; PGP9.5: ubiquitin thiolesterase; MAGEA1: MAGE family member A1.*

**References.**

1. Toi Y, Sugawara S, Sugisaka J, *et al.* Profiling preexisting antibodies in patients treated with anti-PD-1 therapy for advanced non-small cell lung cancer. JAMA Oncol. 2019 Mar 1;5(3):376-383. [https://doi.org/10.1001/jamaoncol.2018.5860.](1https:/doi.org/10.1001/jamaoncol.2018.5860.)
2. Ugolini A, Zizzari IG, Ceccarelli F, *et al.* IgM-Rheumatoid factor confers primary resistance to anti-PD-1 immunotherapies in NSCLC patients by reducing CD137^+^ T-cells. EBioMedicine. 2020 , 62:103098. [https://doi.org/10.1016/j.ebiom.2020.103098.](2https:/doi.org/10.1016/j.ebiom.2020.103098. )
3. Ohue Y, Kurose K, Karasaki T, *et al.* Serum antibody against NY-ESO-1 and XAGE1 antigens potentially predicts clinical responses to anti-programmed cell death-1 therapy in NSCLC. J Thorac Oncol. 2019, 14(12):2071-2083. [https://doi.org/10.1016/j.jtho.2019.08.008.](3https:/doi.org/10.1016/j.jtho.2019.08.008.)
4. Zhou J, Zhao J, Jia Q, *et al.* Peripheral blood autoantibodies against to tumor-associated antigen predict clinical outcome to immune checkpoint inhibitor-based treatment in advanced non-small cell lung cancer. Front Oncol. 2021 , 11:625578. [https://doi.org/10.3389/fonc.2021.625578.](4https:/doi.org/10.3389/fonc.2021.625578.)
5. Tan Q, Wang D, Yang J, *et al.* Autoantibody profiling identifies predictive biomarkers of response to anti-PD1 therapy in cancer patients. Theranostics. 2020 , 10(14):6399-6410. [https://doi.org/10.7150/thno.45816.](5 https:/doi.org/10.7150/thno.45816. )
6. Tan Q, Dai L, Wang Y, *et al*. Anti-PD1/PDL1 IgG subclass distribution in ten cancer types and anti-PD1 IgG4 as biomarker for the long time survival in NSCLC with anti-PD1 therapy. Cancer Immunol Immunother. 2022 Jul;71(7):1681-1691. [https://doi.org/10.1007/s00262-021-03106-z.](6https:/doi.org/10.1007/s00262-021-03106-z.)
7. Patel AJ, Tan TM, Richter AG, *et al.* A highly predictive autoantibody-based biomarker panel for prognosis in early-stage NSCLC with potential therapeutic implications. Br J Cancer. 2022 ;126(2):238-246. [https://doi.org/10.1038/s41416-021-01572-x.](7https:/doi.org/10.1038/s41416-021-01572-x. )
8. Li Y, Li CQ, Guo SJ, *et al.* Longitudinal serum autoantibody repertoire profiling identifies surgery-associated biomarkers in lung adenocarcinoma. EBioMedicine. 2020 Mar;53:102674. [https://doi.org/doi: 10.1016/j.ebiom.2020.102674.](8 https:/doi.org/doi: 10.1016/j.ebiom.2020.102674.)
9. Ma L, Yue W, Teng Y, *et al.* Serum anti-CCNY autoantibody is an independent prognosis indicator for postoperative patients with early-stage nonsmall-cell lung carcinoma. Dis Markers. 2013;35(5):317-25. [https://doi.org/10.1155/2013/935943.](9https:/doi.org/10.1155/2013/935943.)
10. Mattioni M, Soddu S, Prodosmo A, *et al.* Prognostic role of serum p53 antibodies in lung cancer. BMC Cancer. 2015 Mar 18;15:148. [https://doi.org/10.1186/s12885-015-1174-4.](10https:/doi.org/10.1186/s12885-015-1174-4.)
11. Wu WB, Yie SM, Ye SR, *et al.* An Autoantibody against human dna-topoisomerase I is a novel biomarker for non-small cell lung cancer. Ann Thorac Surg. 2018 Jun;105(6):1664-1670. [https://doi.org/10.1016/j.athoracsur.2018.01.036.](11https:/doi.org/10.1016/j.athoracsur.2018.01.036.)
12. Chen SS, Li K, Wu J, *et al.* Stem signatures associated antibodies yield early diagnosis and precise prognosis predication of patients with non-small cell lung cancer. J Cancer Res Clin Oncol. 2021 Jan;147(1):223-233. [https://doi.org/10.1007/s00432-020-03325-4.](12https:/doi.org/10.1007/s00432-020-03325-4.)

**Table S8. The specific autoantibody markers reported in the literature associated with therapeutic and prognosis efficacy in NSCLC.**

|  | **Gene name**  **(*Huprot name*)** |  | **Discovery Panel ID** |  | **Verification Panel ID** |
| --- | --- | --- | --- | --- | --- |
| 1 | ANAs | ✔ | - | ✔ |  |
| 1-1 |  | ✔ | Auto-antigen.B20C4R39 | ✘ | N/A |
| 1-2 |  | ✔ | Auto-antigen.B20C24R39 | ✘ | N/A |
| 1-3 |  | ✔ | Auto-antigen.B20C6R41 | ✔ | Auto-antigenP267A09 |
| 1-4 |  | ✔ | Auto-antigen.B20C16R41 | ✘ | N/A |
| 1-5 |  | ✔ | Auto-antigen.B20C4R41 | ✔ | Auto-antigenP267A11 |
| 1-6 |  | ✔ | Auto-antigen.B20C6R39 | ✘ | N/A |
| 2 | Thyroglobulin | ✘ | N/A | ✘ | N/A |
| 3 | Thyroid peroxidase (TPO) | ✘ | N/A | ✘ | N/A |
| 4 | lgM-RF | ✘ | N/A | ✘ | N/A |
| 5 | NY-ESO-1(CTAG1A) | ✘ | N/A | ✘ | N/A |
| 6 | XAGE1(XAGE1A) | ✘ | N/A | ✘ | N/A |
| 7 | Tp53 | ✔ | JHU04788.B3C9/JHU13973.B10C27 | ✔ | JHU04788P050D05 |
| 8 | HUD(ELAVL4) | ✘ | N/A | ✘ | N/A |
| 9 | BRCA2 | ✔ | JHU25607.B17C24 | ✘ | N/A |
| 10 | TRIM21 | ✔ | JHU00287.B14C24 | ✔ | JHU00287P235E08 |
| 11 | SIX2 | ✔ | JHU00746.B2C13 | ✔ | JHU00746P008F10 |
| 12 | EIF4E2 | ✔ | JHU02721.B3C20/JHU15310.B9C23 | ✔ | JHU02721P029C11 |
| 13 | anti-PD1 IgG4 | ✘ | N/A | ✘ | N/A |
| 14 | SPATA19 | ✔ | JHU07283.B10C22 | ✘ | N/A |
| 15 | TSPY3 | ✔ | JHU10609.B16C3/JHU10609.B8C16 | ✘ | N/A |
| 16 | GLS2 | ✔ | JHU17986.B16C11 | ✘ | N/A |
| 17 | TCEA2 | ✔ | JHU04308.B2C15/JHU06417.B6C25/JHU11693.B12C8 | ✘ | N/A |
| 18 | TSGA10 | ✔ | JHU06137.B8C18 | ✘ | N/A |
| 19 | HMGN5 | ✔ | JHU01985.B15C13 | ✘ | N/A |
| 20 | LUZP4 | ✘ | N/A | ✘ | N/A |
| 21 | HDAC4 | ✔ | JHU07041.B5C9 | ✘ | N/A |
| 22 | SPACA3 | ✔ | JHU10918.B6C29 | ✘ | N/A |
| 23 | IMPDH1 | ✔ | JHU04168.B4C30/JHU09734.B8C29 | ✘ | N/A |
| 24 | TXN2 | ✔ | JHU01053.B13C6 | ✘ | N/A |
| 25 | TFG | ✔ | JHU01525.B4C28 | ✘ | N/A |
| 26 | PPP2R1A | ✔ | JHU05345.B3C31 | ✔ | JHU05345P056D12 |
| 27 | LSP1 | ✔ | JHU09072.B7C4 | ✘ | N/A |
| 28 | SPP1 | ✔ | JHU04492.B1C11 | ✘ | N/A |
| 29 | PVALB | ✔ | JHU10147.B7C22 | ✘ | N/A |
| 30 | RGS20 | ✔ | JHU07939.B5C1 | ✘ | N/A |
| 31 | SNRPA | ✔ | JHU02190.B3C4 | ✘ | N/A |
| 32 | CDH12 | ✔ | JHU08176.B8C22 | ✘ | N/A |
| 33 | CCNY | ✔ | JHU11339.B7C19 | ✘ | N/A |
| 34 | TOPO48(SCL-70) | ✔ | Auto-antigen.B20C6R41 | ✔ | Auto-antigenP267A09 |
| 35 | SOX2 | ✔ | JHU04491.B15C14 | ✘ | N/A |
| 36 | PGP9.5(UCHL1) | ✔ | JHU04505.B3C25 | ✘ | N/A |
| 37 | MAGEA1 | ✔ | JHU18291.B15C8 | ✘ | N/A |

*Abbreviation:✔: represents the antibody is present in discovery or validation cohorts; ✘: represents the antibody is not present in discovery or validation cohorts.*

**Table S9.** **Comparisons of OD values for the MAX and DHX29 AAbs between responders (*n*=84) and non-responders (*n*=29) in the validation cohort.**

| **AAbs** | **Responders(*n*=84)** | **Non-Responders(*n*=39)** | ***p.Value*** |
| --- | --- | --- | --- |
| MAX | 1.26±0.47 | 1.03±0.43 | *0.02* |
| DHX29 | 0.95±0.43 | 0.72±0.30 | *0.0062* |

*Abbreviation: OD: optical density; AAbs: autoantibodies; Responder: patients achieving complete remission, partial remission, or stable disease within three months of treatment; Non-Responder: patients achieving disease progression within three months of treatment.*

**Table S10. Comparisons of OD values for MAX AAb pre- and on-chemoimmunotherapy in the validation cohort.**

| **ID** | **Response** | **MAX** | |
| --- | --- | --- | --- |
|  |  | Pre-chemoimmunotherapy | On-chemoimmunotherapy |
| 1 | Responder | 1.506 | 1.793 |
| 2 | Responder | 1.393 | 1.485 |
| 3 | Responder | 1.437 | 1.861 |
| 4 | Responder | 0.763 | 0.778 |
| 5 | Responder | 1.045 | 1.047 |
| 6 | Responder | 1.183 | 1.684 |
| 7 | Responder | 1.027 | 1.105 |
| 8 | Responder | 0.539 | 0.694 |
| 9 | Responder | 0.903 | 1.318 |
| 10 | Responder | 1.672 | 1.818 |
| 11 | Responder | 1.608 | 1.886 |
| 12 | Responder | 2.384 | 2.362 |
| 13 | Responder | 1.056 | 1.965 |
| 14 | Responder | 1.892 | 2.053 |
| 15 | Responder | 1.152 | 1.769 |
| 16 | Responder | 1.250 | 1.661 |
| 17 | Responder | 0.862 | 0.950 |
| 18 | Responder | 0.927 | 1.101 |
| 19 | Responder | 1.067 | 1.272 |
| 20 | Responder | 0.837 | 1.044 |
| 21 | Responder | 1.114 | 1.403 |
| 22 | Responder | 1.405 | 1.873 |
| 23 | Responder | 1.902 | 1.883 |
| 24 | Responder | 0.807 | 1.271 |
| 25 | Non-Responder | 0.914 | 1.368 |
| 26 | Non-Responder | 0.584 | 0.869 |
| 27 | Non-Responder | 0.620 | 0.728 |
| 28 | Non-Responder | 0.959 | 1.280 |
| 29 | Non-Responder | 0.900 | 2.021 |
| 30 | Non-Responder | 0.798 | 1.254 |
| 31 | Non-Responder | 1.695 | 2.038 |
| 32 | Non-Responder | 1.122 | 1.545 |
| 33 | Non-Responder | 0.974 | 1.558 |
| 34 | Non-Responder | 1.581 | 1.814 |
| 35 | Non-Responder | 1.775 | 1.330 |
| 36 | Non-Responder | 0.587 | 0.714 |
| 37 | Non-Responder | 1.095 | 1.366 |
| 38 | Non-Responder | 1.546 | 1.453 |
| 39 | Non-Responder | 0.542 | 0.736 |
| 40 | Non-Responder | 2.431 | 2.257 |

*Abbreviation: OD: optical density; AAbs: autoantibodies; Responder: patients achieving complete remission, partial remission, or stable disease within three months of treatment; Non-Responder: patients achieving disease progression within three months of treatment.*

**Table S11. MAX mRNA expression levels along with survival clinical data from two GEO datasets for NSCLC.**

| **GSE135222** | | | | |
| --- | --- | --- | --- | --- |
| **Samples** | **MAX** | **PFS state** | **PFS time** | **Benefit** |
| NSCLC378 | 5.371558863 | 0 | 13.73333333 | DNB |
| NSCLC1104 | 6.946262984 | 1 | 0.966666667 | NDB |
| NSCLC947 | 5.717539343 | 0 | 20.6 | DNB |
| NSCLC990 | 6.390082951 | 1 | 3.166666667 | NDB |
| NSCLC1203 | 5.033423002 | 1 | 0.833333333 | NDB |
| NSCLC1327 | 5.56193706 | 0 | 6.833333333 | DNB |
| NSCLC1401 | 7.226893814 | 1 | 0.933333333 | NDB |
| NSCLC1155 | 5.149340669 | 1 | 1.133333333 | NDB |
| NSCLC573 | 7.972577785 | 1 | 1.5 | NDB |
| NSCLC825 | 5.622637567 | 1 | 1.433333333 | NDB |
| NSCLC1528 | 4.66106548 | 0 | 9.3 | DNB |
| NSCLC1508 | 5.661635602 | 1 | 2.166666667 | NDB |
| NSCLC1708 | 5.551208374 | 1 | 5.6 | DNB |
| NSCLC1352 | 5.648177796 | 0 | 8.333333333 | DNB |
| NSCLC1066 | 5.364222221 | 1 | 0.766666667 | NDB |
| NSCLC1017 | 5.85124932 | 1 | 1.266666667 | NDB |
| NSCLC1358 | 5.513174885 | 1 | 8.566666667 | DNB |
| NSCLC1510 | 6.093179847 | 0 | 10.8 | DNB |
| NSCLC1164 | 5.101818134 | 1 | 0.366666667 | NDB |
| NSCLC1412 | 5.352264173 | 1 | 0.1 | NDB |
| NSCLC1145 | 5.531381461 | 1 | 2.433333333 | NDB |
| NSCLC1425 | 6.006298024 | 1 | 1.966666667 | NDB |
| NSCLC1554 | 5.198494154 | 1 | 1.233333333 | NDB |
| NSCLC1619 | 5.46662712 | 1 | 2.733333333 | NDB |
| NSCLC1809 | 5.416839742 | 1 | 1.466666667 | NDB |
| NSCLC1873 | 5.357903839 | 1 | 1.233333333 | NDB |
| NSCLC1079 | 4.144862143 | 1 | 5.8 | NDB |
| **GSE126044** | | | | |
| **symbol** | **MAX** | **PFS state** | **PFS time** | **Response** |
| Dis_01 | 10.61930296 | 1 | 0.5 | PD |
| Dis_02 | 11.1382718 | 1 | 13.83333333 | PR |
| Dis_03 | 11.33873638 | 1 | 2.3 | PD |
| Dis_04 | 11.12605912 | 1 | 13.5 | PR |
| Dis_05 | 11.3376219 | 1 | 2.8 | PD |
| Dis_06 | 11.25561875 | 0 | 0.8 | PD |
| Dis_07 | 11.27670602 | 1 | 1.1 | PD |
| Dis_08 | 11.74986943 | 0 | 1 | PD |
| Dis_09 | 11.70520038 | 1 | 0.7 | PD |
| Dis_10 | 11.45738088 | 0 | 14.23333333 | PR |
| Dis_11 | 13.27364992 | 1 | 2.133333333 | PD |
| Dis_12 | 10.34096276 | 1 | 3.433333333 | PD |
| Dis_15 | 10.86418614 | 1 | 17.73333333 | PR |
| Dis_16 | 10.73893668 | 1 | 3.566666667 | PD |
| Dis_17 | 10.68123841 | 1 | 7.333333333 | SD |
| Dis_18 | 9.705632387 | 1 | 0.533333333 | PD |

*Abbreviation: mRNA: messenger RNA; GEO: Gene Expression Omnibus; NSCLC: non small-cell lung cancer; PFS: progression-free survival; DNB: durable clinical benefit; NDB: no durable benefit; PR: partial remission; SD: steady disease; PD: progression disease.*
